# Remote functionalization of hydrocarbons with reversibility enhanced stereocontrol†
†Electronic supplementary information (ESI) available: Experimental procedures, computational details, list of coordinates and energies (E, H and G) of optimized structures, spectroscopic data and copies of ^1^H and ^13^C NMR spectra. See DOI: 10.1039/c5sc00445d



**DOI:** 10.1039/c5sc00445d

**Published:** 2015-03-03

**Authors:** Alexandre Vasseur, Lionel Perrin, Odile Eisenstein, Ilan Marek

**Affiliations:** a The Mallat Family Laboratory of Organic Chemistry , Schulich Faculty of Chemistry and Lise Meitner-Minerva Center for Computational Quantum Chemistry , Technion-Israel Institute of Technology , Technion City , Haifa 32000 , Israel . Email: chilanm@tx.technion.ac.il ; Fax: +972-4-829-37-09 ; Tel: +972-4-829-37-09; b ICBMS UMR 5246 , Université de Lyon , Bat Curien, 43 Bd du 11 Novembre 1918 , 69622 Villeurbanne cedex , France . Email: lionel.perrin@univ-lyon1.fr; c Institut Charles Gerhardt , CNRS UMR 5253 , Université de Montpellier , cc 1501, Place E. Bataillon , 34095 Montpellier , France

## Abstract

Remote functionalization of hydrocarbons could be achieved through successive zirconocene-mediated allylic C–H bond activations followed by a selective C–C bond cleavage. Determination of the reaction mechanism by density functional theory (DFT) calculations shows that the high stereocontrol observed in this process results from a large number of energetically accessible equilibria feeding a preferred reactive channel that leads to the major product. A distinctive consequence of this pattern is that stereoselectivity is enhanced upon heating.

## Abstract 2

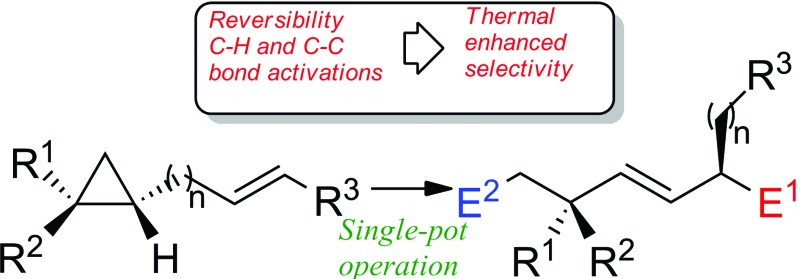
Remote functionalization of hydrocarbons could be achieved through successive zirconocene-mediated allylic C–H bond activations followed by a selective C–C bond cleavage.

## Introduction

Manipulation of functionality at a specific position of a hydrocarbon that would generate a reaction at a different location represents a major challenge in synthetic organic chemistry. The difficulty of such remote functionalization is even more pronounced for acyclic systems where flexible alkyl chains are present between the initiating and the final reactive centers. Since the pioneering work on remote functionalization of Breslow's in the 70's,^1^ numerous studies have appeared for the relay or transmission of stereochemical information along alkyl chains.^2^

**Scheme 1:**
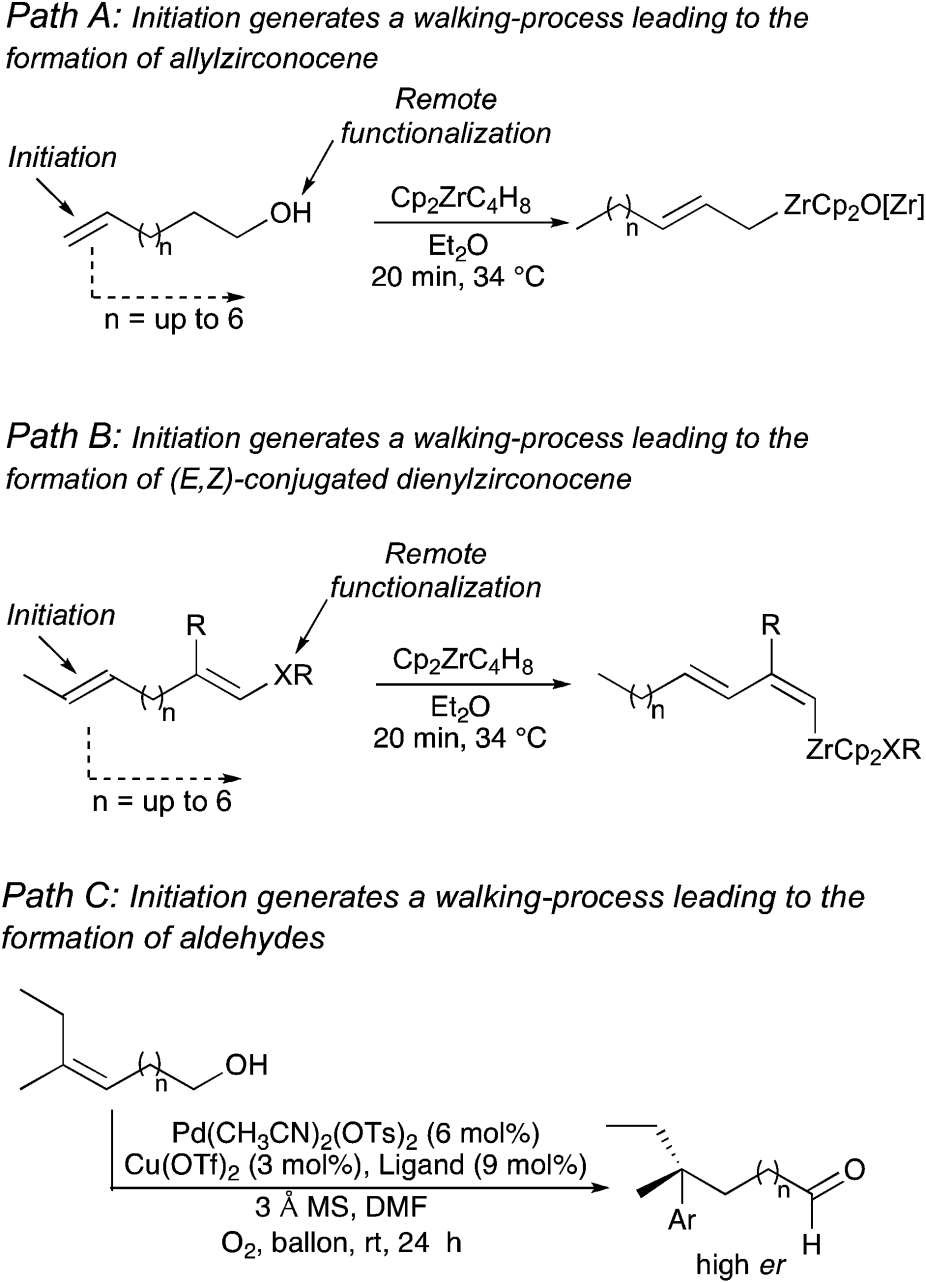
General approach for remote functionalization.

In this context, a particularly impressive example in the field of asymmetric induction is the foldamer-mediated 1,61-asymmetric remote induction reported by Clayden.^3^ On the other hand, as transition-metal complexes have the ability to isomerize double bonds along a carbon-skeleton,^4–6^ one could design a system where the isomerization would generate a selective remote transformation. However, due to the natural propensity for statistical isomerization, the selective migration of a metal complex along a hydrocarbon chain can only be directed if associated with a strongly thermodynamically favoured termination step. In this context, we have reported the transformation of unsaturated fatty alcohol derivatives as a source of substituted allylmetal species (Path A, Scheme 1)^7^ and the stereoselective preparation of conjugated dienyl metal complexes from non-conjugated enol ethers (Path B, Scheme 1)^8^ where the irreversible termination step is an elimination reaction. Initiating reversible allylic C–H bond activations triggered these reactions and up to 6 carbon atoms separated the initial double bond from the terminating center. In contrast, a very appealing unidirectional palladium-chain walking for the enantioselective redox-relay Heck-type arylation of alkenyl alcohols was reported where an oxidative deprotonation of the final α-alkoxy palladium–alkyl intermediate closes the catalytic cycle (Path C, Scheme 1).^9^

**Scheme 2:**
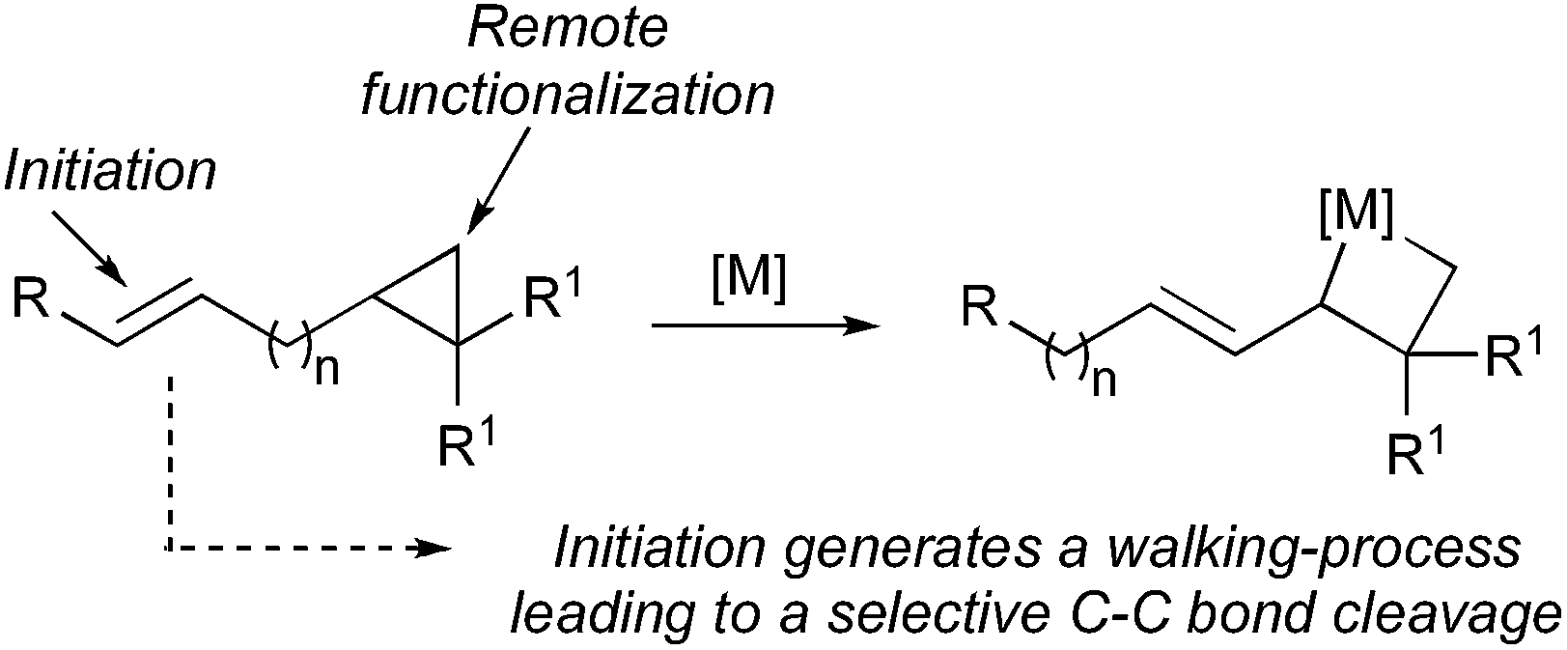
Proposed approach for remote functionalization of hydrocarbons.

However, reaction of a metal complex with a functionality that would generate a final unidirectional “walking-process” over an alkyl chain of an hydrocarbon (only composed of H and C atoms)^4^ producing a chemical reaction at a defined terminus position is a very promising, but still in its infancy, approach to functionalize molecules.^5,6^ To answer this challenging remote functionalization of hydrocarbons, we were interested to investigate the case of ω-ene cyclopropanes. If the trigger promotes the walking-process of a double bond to finally lead to a selective C–C bond carbon cleavage (Scheme 2), two cutting-edge methods of activation (C–H and C–C bond cleavage) would be unified into a single method through the use of a unique organometallic species.^10^

## Results and discussion

**Scheme 3:**
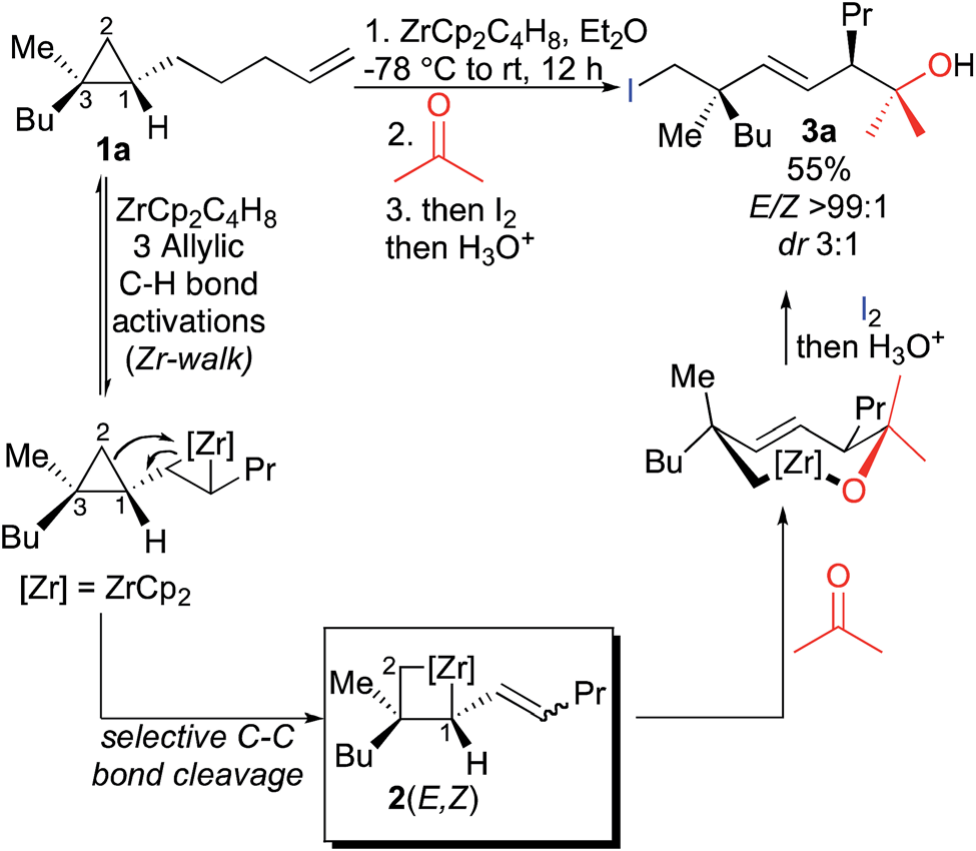
1,4-Diastereoselectivity in the zirconocene-promoted allylic C–H bond activation and C–C bond cleavage.

We have shown that ω-ene cyclopropane **1a** (Scheme 3) as well as alkylidenecyclopropanes (not described) can easily undergo a zirconocene-mediated allylic C–H bond activation followed by highly selective C–C bond cleavage.^10^ It should be noted that under the same experimental conditions, saturated cyclopropane (not possessing the remote double bond) does not lead to the carbon–carbon bond cleavage of the three-membered ring, confirming our hypothesis that the initiating step on the alkenyl moiety is required for the reaction to proceed. The allylic C–H bond activation most probably proceeds through the formation of an η^3^-allyl intermediate^7,8^ and the unique selectivity of the ring-opening results from the kinetically and thermodynamically preferred formation of primary organometallic species **2** over tertiary organometallic species in the ring cleavage (C_1_–C_2_ cleavage preferred over C_1_–C_3_, Scheme 3).^11^ Moreover, the reactivity of the bismetalated species (also called metallacycle) **2** can be fully controlled as the reactivity of the allylzirconocene moiety (C_1_–Zr) is higher than the reactivity of the alkylzirconocene (C_2_–Zr) moieties towards electrophiles.^12^

**Fig. 1:**
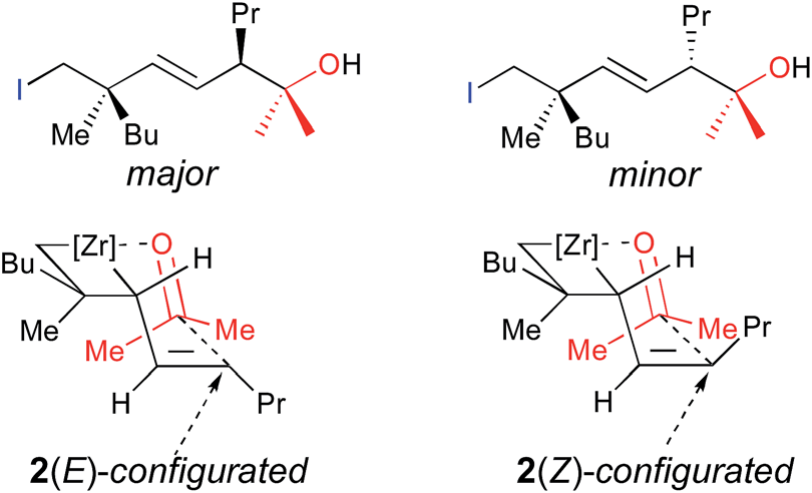
Proposed Zimmermann–Traxler transition state.

Therefore, addition of a carbonyl group to the first electrophile such as acetone leads to the unique formation of functionalized adducts at the allylic position, that could be also eventually represented as the cyclic mono-metallated cyclooctenolate derivative, and the second electrophile (*i.e.* I_2_) gives the final *E*-adduct **3a** in 55% yield (Scheme 3).^10^ Although this strategy holds potential for 1,4-induction of diastereoselectivity with formation of the highly valued quaternary carbon and tertiary stereocenters in 1,4-relationship in an acyclic system,^13,14^ we could never reach a decent diastereoselectivity from ω-ene cyclopropanes (dr 3 : 1, Scheme 3). This low 1,4-diastereoselectivity in the formation of the linear product was rationalized by the presence of a mixture of two geometrical *E*- and *Z*-isomers of the substituted allylzirconocene **2**,^10^ both reacting with acetone through a chair-like transition state^15^ as described in Fig. 1 leading to the *E*-isomer **3a** as a mixture of two sp^3^ centered diastereoisomers. In this communication, we report how we could improve the diastereoselectivity of the reaction and rationalize the results.

Following our previous work on the zirconocene-mediated ring-opening of alkylidenecyclopropanes,^10^ we hypothesized that the diastereoselectivity could be improved if one could isomerize the (*Z*)-configured substituted allylzirconocene **2** into the thermodynamically more stable **2**(*E*)-isomer. When the diastereomerically pure ω-ene cyclopropanes **1b,e** were treated with the Negishi reagent^16^ at room temperature overnight in Et_2_O, the intermediate metallacycles **2b,e** were initially obtained as two geometrical (*E*,*Z*)-isomers. We were delighted to observe that the isomerization of the substituted allylzirconocene into the single *E*-allylzirconocene species **2** could be promoted by addition of THF as a co-solvent and heating to 55 °C for 3 h. Then, the addition of the carbonyl groups followed by the second electrophile gave diastereoisomerically enriched **3b–m** as described in Table 1. It should be noted that neither the addition of THF alone without heating nor heating (in Et_2_O) without addition of the cosolvent THF was sufficient to fully isomerize the (*Z*)-allylzirconocenes **2** into the (*E*)-isomer. We hypothesized that the addition of this co-solvent is needed to reach a temperature high enough to promote the isomerization of the *Z*- into *E*-allylzirconocene. In all cases, the combined C–H allylic bond activation followed by the selective C–C bond cleavage leads, after isomerization of the substituted allylzirconocene and reaction with two different electrophiles, to acyclic *E*-alkenes possessing two stereogenic centers in a 1,4-relationship, including the quaternary carbon stereocenter with very high diastereoselectivity. It should be stressed that the selectivity of the ring-cleavage is complete as no trace of activation along the C_1_–C_3_ bond was detected in the crude reaction mixture. This tandem reaction is not limited to a one-carbon tether (Table 1, entries 1–5) as it could be extended similarly to longer alkyl chains (Table 1, entry 6, *n* = 2, entries 7–11, *n* = 3 and entry 12, *n* = 4) with similar selectivities. When the migrating double bond is 1,2-disubstituted such as in **1c** (Table 1, entry 6), our tandem sequence of allylic C–H isomerization/carbon–carbon cleavage still proceeds very efficiently as **3g** is obtained in 62% yield with a 1,4-diastereoisomeric ratio of 98 : 2. It should be noted that although **1c** was present as two geometrical (*E*)/(*Z*)-isomers in a 1 : 1 ratio, only the (*E*)-isomer of **3g** is obtained.

**Table 1:** 1,4-Diastereoselectivity in the zirconocene-promoted allylic C–H bond activation and C–C bond cleavage

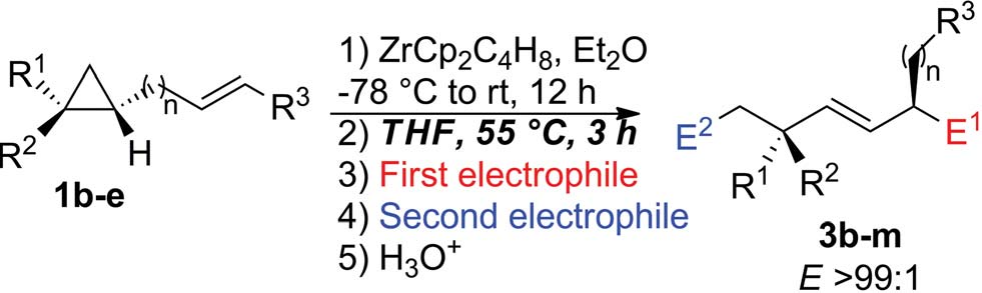								
Entry	R^1^	R^2^	R^3^	*n*	E^1^	E^2^	dr^*a*^	Yield^*b*^ (%)
1	Pr	Bu	H	1(**1b**)	MeCOMe	H_3_O^+^	98 : 2	83 (**3b**)
2	Pr	Bu	H	1(**1b**)	[CH_2_]_4_CO	H_3_O^+^	98 : 2	62 (**3c**)
3	Pr	Bu	H	1(**1b**)	[CH_2_]_5_CO	H_3_O^+^	98 : 2	52 (**3d**)
4	Pr	Bu	H	1(**1b**)	EtCOEt	I_2_	98 : 2	56 (**3e**)
5	Pr	Bu	H	1(**1b**)	MeCOMe	I_2_	98 : 2	51 (**3f**)
6	Et	Bu	Me	2(**1c**)	MeCOMe	H_3_O^+^	98 : 2	62 (**3g**)
7	Et	Bu	H	3(**1d**)	MeCOMe	H_3_O^+^	98 : 2	61 (**3h**)
8	Et	Bu	H	3(**1d**)	[CH_2_]_4_CO	H_3_O^+^	97 : 3	61 (**3i**)
9	Et	Bu	H	3(**1d**)	[CH_2_]_5_CO	H_3_O^+^	98 : 2	62 (**3j**)
10	Et	Bu	H	3(**1d**)	MeCOMe	I_2_	96 : 4	55 (**3k**)
11	Et	Bu	H	3(**1d**)	EtCOEt	H_3_O^+^	98 : 2	59 (**3l**)
12	Et	Bu	H	4(**1e**)	MeCOMe	H_3_O^+^	98 : 2	50 (**3m**)

^*a*^Determined by ^1^H NMR analysis of crude reaction mixture.

^*b*^Determined after purification by column chromatography on silica gel.

**Scheme 4:**
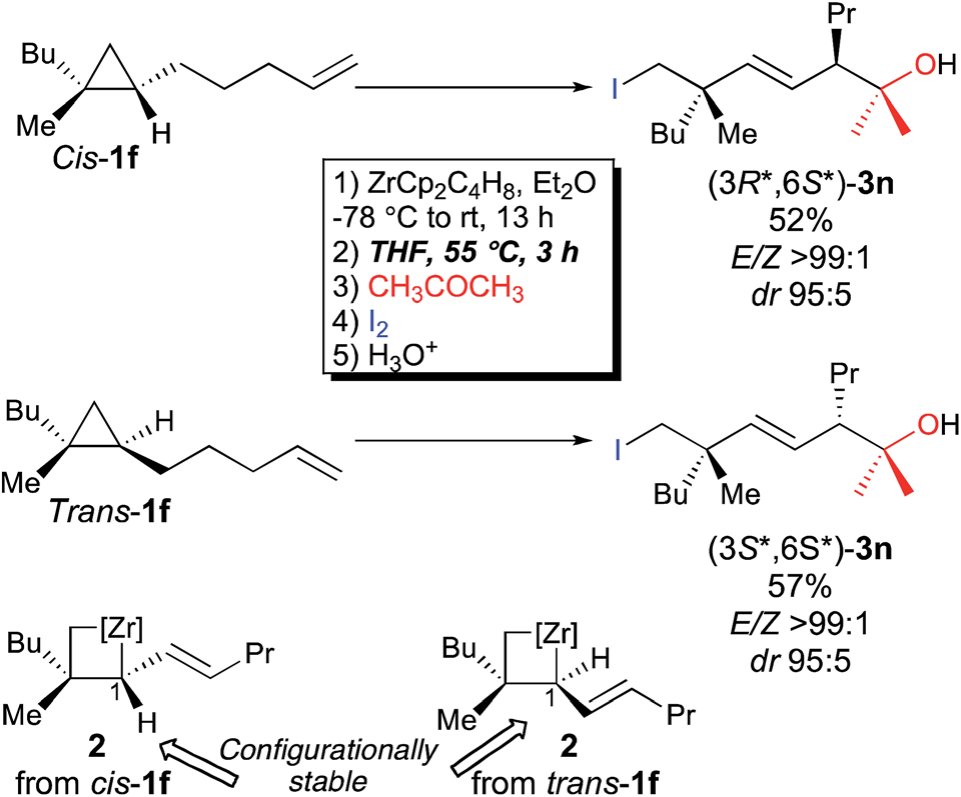
Tandem reactions on the two opposite diastereoisomers of **1f**.

**Fig. 2:**
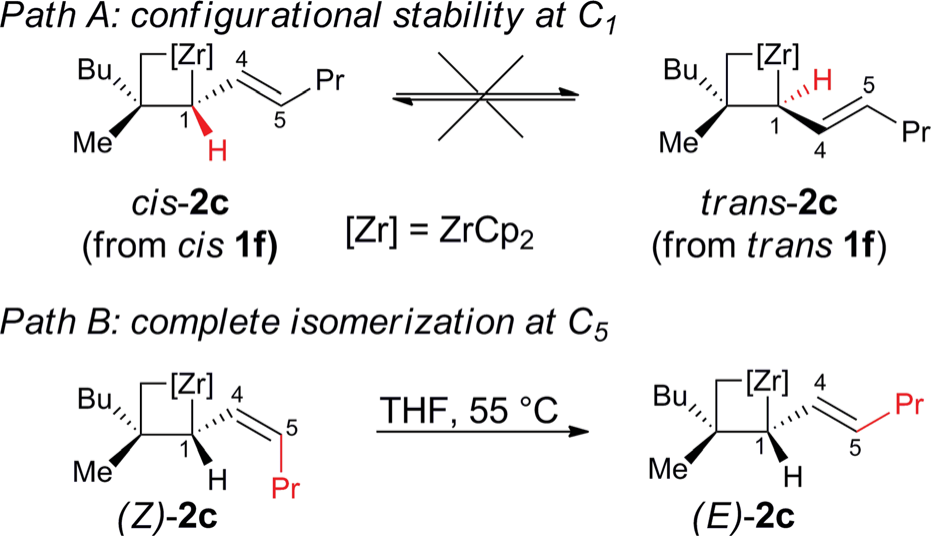
Configurational stability at C_1_ and isomerization at C_5_.

Importantly, when the two diastereoisomers of ω-ene cyclopropanes **1f** are subjected to our experimental conditions, the two opposite diastereoisomers of **3n** are obtained with comparable diastereomeric ratios and yields (Scheme 4). In addition to the synthetic importance of this transformation, this result clearly shows that the C_1_–[Zr] bond of zirconacyclobutane intermediates **2** is configurationally stable. In other words, the two independently formed intermediate alkyl–allyl zirconacyclobutanes *cis*-**2c** and *trans*-**2c** respectively do not interconvert at the metalated center C_1_ (Path A, Fig. 2) despite the isomerization of the (*Z*)-**2c** into (*E*)-**2c** allylzirconocene fragment at C_5_ by heating at 55 °C for 3 h (Path B, Fig. 2).

To gain a better understanding of the mechanism of this surprising *Z*- to *E*-isomerization of the allylzirconocene fragment on C_5_ without eroding the configurational stability at C_1_ of the zirconacyclobutane **2c**, this reaction was studied with density functional theory (DFT) calculations using the M06 functional that includes dispersion contributions.^17^ An implicit solvent model was used for modeling bulk solvation by Et_2_O according to the SMD method. Gibbs free energies calculated at *T* = 298.15 K and *P* = 1 atm are used to discuss the reaction pathways. More details are given in the ESI.†

**Fig. 3:**
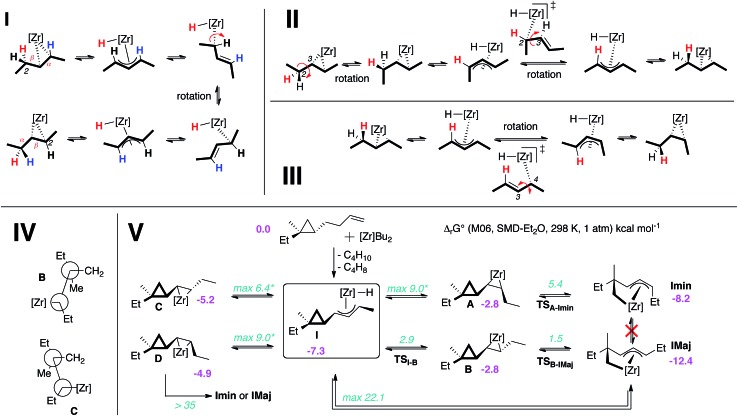
Reaction pathways showing the key extrema for zirconocene (I) chain walking; (II) face switching; (III) *E* to *Z*-olefin isomerization; (IV) Newman projection for complexes **B** and **C** to illustrate the *anti*- and *syn*-periplanar configurations between the 3-membered rings; (V) reaction pathways involved in the formation of *cis*-**2c** and *trans*-**2c**. Extrema are labeled in bold black. Gibbs free energies are in kcal mol^–1^ relative to separated [Zr]Bu_2_ and substrate taken as energy reference with values for minima and transition states in pink and teal, respectively. In multistep sequences, max refers to the Gibbs free energy of the highest transition state. The sign * refers to values obtained for pent-2-ene as substrate.

Experimentally, the chain walking mechanism has been established by deuterium labeling. It was shown that successive migration of the double bond is promoted by allylic C–H bond activations to yield Zr–hydride–allyl intermediate complexes as depicted in Fig. 3I.^7a,8a,10a^ Calculations show that this mechanism is energetically preferred for the chain walking along the alkenyl chain of a cyclopropane substrate. When moving the double bond of one carbon unit, the rate-determining step involves the rotation along the Zr–C bond of the σ-allyl ligand. The highest transition state is 6 kcal mol^–1^ above the initial reactants taken as energy reference hereafter. Calculations also reveal the existence of a complex set of equilibria built on rotations within σ-allyl ligands at the transient zirconocene–hydride–allyl complexes (Fig. 3I–III) that was characterized for a related Zr–allyl complex.^18^ These equilibria are kinetically and thermodynamically comparable leading to *kinetically and thermodynamically unselective chain walking* (zirconocene can switch between the alkene faces (Fig. 3II) and isomerize the *Z*- into the *E*-olefin, Fig. 3III). A fully detailed computational mechanistic of these isomerizations and others will be given in a forthcoming report.

The ability to transform the olefin complex into a hydride allylic complex where many isomerizations can be achieved at a low energy cost is thus an important feature of this system. Indeed, the Zr-promoted walking process potentially leads to a mixture of four cyclopropyl diastereoisomeric zircona–cyclopropane complexes (**A** to **D**, Fig. 3V).^17^ These four intermediates are in equilibrium *via* the isomerization aforementioned and share **I** as the most stable common intermediate, which is 7 kcal mol^–1^ below Cp_2_ZrBu_2_ and free substrate and more stable than **A** to **D**. Therefore, species **I** is the most abundant intermediate before the C–C bond cleavage. Importantly, the relative ratios of these four complexes have little to no consequence on the stereochemical outcome of the reaction as described below.

The four complexes **A** to **D** differ strongly in their ability to undergo the ring opening of the cyclopropyl ring since they are determined by the position of the Zr relative to the ring. An *anti*-periplanar relationship between the zirconacyclopropane and the cyclopropyl ring in **C** and **D** prevents the C_1_–C_2_ ring opening, (activation barrier of *ca.* 40 kcal mol^–1^, Fig. 3IV) whereas a *syn*-periplanar relationship between the reactive function in **A** and **B** leads to activation barriers for the C–C bond cleavage of around 5 kcal mol^–1^. Furthermore, the ring opening of the cyclopropyl ring always occurs at the least substituted carbon–carbon bond as shown in Scheme 3 and Fig. 3V. No transition state for C_1_–C_3_ bond cleavage could be located presumably due to the steric hindrance.

**Fig. 4:**
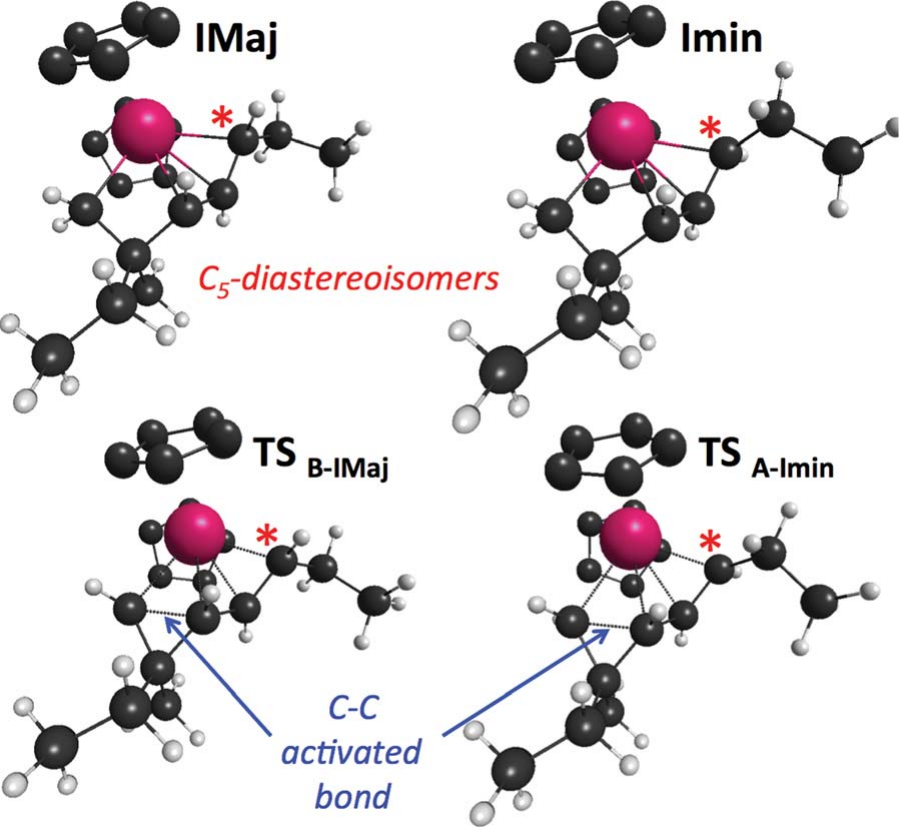
Structures of **Imin**, **IMaj** and their associated transition states of formation.

Cyclopropane ring opening of **A** and **B** respectively yields **Imin** and **IMaj** that are the calculated structures for (*Z*)-**2c** and (*E*)-**2c**, respectively (Fig. 2 and 3). Whereas, isomers **A** and **B** are isoenergetic, **IMaj** is energetically preferred over **Imin** by 4 kcal mol^–1^, an energy difference that is also present in the corresponding transition states. The transition state for the cyclopropane ring opening occurs by approaching the methylene C_2_ to the Zr while maintaining the Zr–β-ene interaction. In both **TS_B–IMaj_** and **TS_A–Imin_**, the Zr–C_2_ bond distance is 2.66 Å and the C_2_–C_1_ is 1.72 Å. Thus, the difference in energy between the two transition states **TS_B–IMaj_** and **TS_A–Imin_** appears to be due to nonbonded interactions between the ethyl chain and the Cp_2_Zr fragment. As illustrated in Fig. 4, these interactions are present in **IMaj** and **Imin** and account for similarity in the kinetic and thermodynamic preferences. Thus, the kinetic preference for going *via* the transition state that forms the thermodynamically preferred **IMaj**, accounts for the experimental 3 : 1 diastereoisomeric ratio of **3** (Scheme 3) when the reaction is performed at room temperature. Importantly, intermediates **C** and **D**, which cannot perform the cyclopropane ring opening, isomerize to either **A** or **B***via* the Zr–allyl hydride intermediate **I** and thus also contribute to product formation.

Calculations indicate that direct isomerization between (*Z*)- and (*E*)-**2c**, (*i.e.* between **Imin** to **IMaj**) is not allowed. This originates from the restricted rotation around C_4_–C_5_ caused by either the presence of a π-bond or the constraint of the 6-membered ring in the two σ-allyl complexes. Alternatively, a mechanism that connects **Imin** to **I** and thus to **IMaj***via* allylic C–H activation and formation of a zirconium–hydride–diene complex has been computed; the highest transition state of this pathway is located at 22 kcal mol^–1^ above energy reference.^19^ Consequently, the most energetically preferred pathway from **Imin** to **IMaj** is *via***A**, **I** and **B** with the highest transition state being 10 kcal mol^–1^ above the energy reference. This isomerization between **A**, **B**, **C** and **D***via***I** controls the stereoselectivity of the reaction and highlights the importance of the reversibility of the allylic C–H bond activation and C–C bond cleavage by heating the reaction mixture at 55 °C for 3 h.

These computations show that the high diastereoselectivity of this reaction is not due to the existence of a single preferred path but to a large number of energetically accessible equilibria that enables the isomerization of all intermediates into a single one, responsible for the formation of the major product. This mechanism of dynamic thermodynamic resolution has already been reported in the literature mainly for the equilibration of sp^3^ alkyllithium species,^20^ but also for the formation of substituted allyllithium species with sparteine.^21^ Having now a good understanding of the reaction mechanism, we turned our attention to the last synthetic challenge, namely the functionalization of the second Csp^3^–[Zr] bond (second electrophile) through the creation of a new C–C bond.

**Scheme 5:**
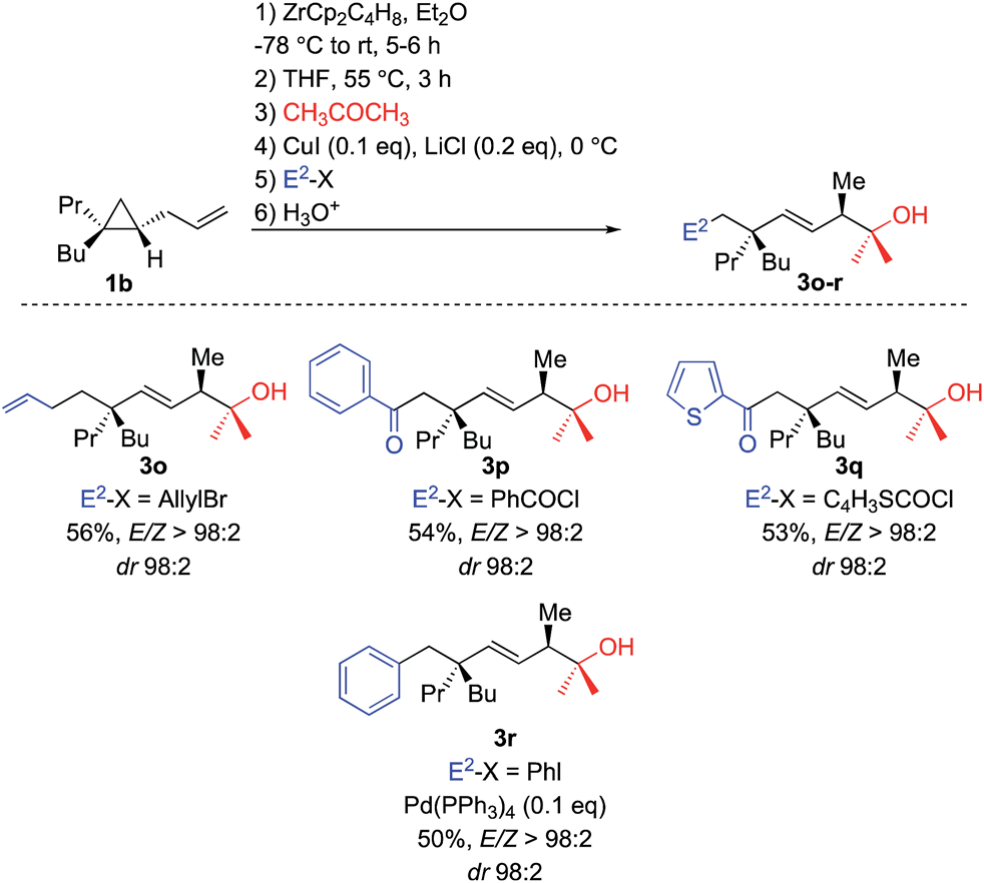
Transmetalation of Csp^3^–[Zr] bond for further C–C bond forming processes.

To perform such C–C bond forming events, transmetalation reactions usually provide a unique and powerful means of expanding the synthetic scope of organozirconium chemistry.^22^ Once the tandem allylic C–H and selective C–C activations is performed on **1b**, THF is added and the solution is heated at 55 °C for 3 h. Then acetone was first added and the resulting alkyl zirconium species was transmetalated into alkyl copper species by addition of catalytic amount of copper salt.^23^ The *in situ* generated alkyl copper can then react with classical electrophiles of copper chemistry such as allyl bromide and aromatic as well as heteroaromatic acyl chloride to give functionalized adducts (Scheme 5, formation of **3o–q**, respectively). The corresponding alkyl copper species could also be transmetalated into palladium by addition of 10 mol% of Pd(PPh_3_)_4_ and coupled with aromatic iodide (formation of **3r**, Scheme 5).

## Conclusions

In conclusion, a diastereoselective remote functionalization of ω-ene cyclopropane species is reported that could lead, in addition, to the formation of functionalized adducts with high 1,4-diastereocontrol. As highly enantiomerically enriched cyclopropane derivatives are easily accessible,^24^ this reaction could represent an interesting entry to the formation of enantiomerically enriched quaternary and tertiary carbon stereocenters in acyclic systems. Computational studies reveal an original way to achieve high stereocontrol by having a plethora of equilibria feeding a preferred reactive channel leading to the major isomer through thermodynamic control. This accounts for the counter-intuitive result that stereocontrol is enhanced by thermal treatment.

## Supplementary Material

Supplementary information
