# Supplementary material for: Remote functionalization of hydrocarbons with reversibility enhanced stereocontrol
Source: Chem Sci. 2015 Mar 3;6(5):2770–6. doi: 10.1039/c5sc00445d (PMC5654413; doi:10.1039/c5sc00445d)
Supplement: Supplementary file 1 [file SC-006-C5SC00445D-s001.pdf]

Electronic Supplementary Material (ESI) for Chemical Science.
This journal is © The Royal Society of Chemistry 2015

# Remote Functionalization of Hydrocarbons with Reversibility Enhanced Stereocontrol**

*Alexandre Vasseur, Lionel Perrin*, Odile Eisentein and Ilan Marek**

**Table of Contents**

General information ........ S2

General procedure for the synthesis of **3b-d**, **3g-j** and **3l-m** ........ S3

General procedure for the synthesis of **3e-f**, **3k** and **3n** ........ S3

Typical procedure for the synthesis **3o** ........ S3

General procedure for the synthesis of **3p-q** ........ S4

Typical procedure for the synthesis **3r** ........ S4

Characterization data of the products ........ S5

Pathways considered for the computational study ........ S10

Computational details ........ S11

Cartesian coordinates ........ S11

References ........ S26

NMR spectra ........ S27

**General information.**

All reactions were carried out under an atmosphere of argon, with flame-dried flasks under positive pressure of argon and with dry, oxygen-free solvents using standard Schlenk techniques. All reagents were purchased from commercial suppliers and used without further purification. All organometallic compounds, dry solvents and reagents were transferred using plastic single-use graduated syringes and oven-dried stainless steel needles. *n*-BuLi (1.6M in hexanes), is commercially obtained from Aldrich and regularly titrated under argon atmosphere by 1M solution of 2-isobutanol in toluene, using 1,10-phenantroline as indicator.

Ketones, allyl bromide, benzoyl chloride and iodobenzene were purchased from commercial suppliers and distilled prior to use under an atmosphere of argon as follows:

- Acetone was distilled from $CaSO_4$,
- Diethyl Ketone was distilled from $P_2O_5$,
- Cyclohexanone and cyclopentanone were distilled from $MgSO_4$,
- Allyl bromide, benzoyl chloride and iodobenzene were distilled from $CaCl_2$.

Progress of the reactions was monitored by analytical TLC using glass plates precoated with silica gel with F254 indicator (Merck). Visualization of spots was done using phosphomolybdic acid followed by heating. Frontal ratios ($R_f$) of each synthesized compounds were determined from the reaction crude by analytical TLC using glass plates precoated with silica gel with F254 indicator (Merck). Purification of crude mixtures was accomplished by column chromatography on silica gel 60 Å (GraceResolv). *E*/*Z* and diastereoisomeric ratios were measured by $^{1}H$ and $^{13}C$ NMR analysis.

$^{1}H$ and $^{13}C$ NMR spectra were measured on Brucker Avance AV300 (300 MHz) spectrometer in $CDCl_3$ and referenced to TMS. Chemical shifts values (δ) are reported in ppm (calibration of spectra to the residual peak of $CDCl_3$: $\delta$ = 7.26 ppm (s) for $^{1}H$ NMR; $\delta$ = 77.16 ± 0.06 ppm for $^{13}C$ NMR). All the proton spectra reported as following: δ value (multiplicity, J coupling constant in Hz, number of nuclei). Multiplicity contractions used: (s) – singlet, (d) – doublet, (dd) – doublet of doublet, (t) – triplet, (q) – quartet, (p) – quintuplet, (m) – multiplet, (bs) – broad singlet. $\omega$-ene cyclopropanes were easily prepared by a standard procedure sequence reported in the literature:[1] Simmons-Smith reaction from tribsubstituted homoallylic alcohols[2], followed by Swern and Wittig reaction respectively.[3]

**General procedure for the synthesis of 3b-d, 3g-j and 3l-m.**

Into a flame-dried, 100-mL three-neck flask, containing a solution of bis(cyclopentadienyl)zirconium dichloride (496.9 mg, 1.7 mmol) in dry $Et_2O$ (10 ml) and equipped with a magnetic stirrer, a low temperature thermometer, a rubber septum and an inert gas inlet, was added dropwise a solution of *n*-butyllithium (1.6 M in hexanes, 2.13 ml, 3.4 mmol) at -78 °C under inert atmosphere. After stirring for 45 min at -78 °C, substrate **1** (1 mmol) diluted in 2 mL of dry $Et_2O$ was added dropwise to the solution at -78 °C. The resulting mixture was allowed to slowly warm-up to room temperature (over 5,5 hours if n = 1 or 12 hours if n = 2 or 3) at which time the reaction mixture is a yellow-orange suspension. Dry THF (40 mL) is then added to the reaction mixture and the resulting solution was heated at 55 °C for 3 hours. The freshly distilled first electrophile (3.0 mmol) is added dropwise after cooling the reaction mixture at 0 °C and the resulting solution was allowed to stir for 2 hours at that temperature. Finally, the reaction mixture is hydrolyzed with an aqueous solution of 1M HCl. The layers were separated and the aqueous phase was extracted with $Et_2O$ (3 times). The combined organic fractions were washed with brine, dried over $MgSO_4$, filtered and concentrated under reduced pressure. The products **3b-d, 3g-j** and **3l-m** were isolated by silica-gel chromatography ($SiO_2$, *n*-pentane / $Et_2O$, 80:20 – 90:10).

**General procedure for the synthesis of 3e-f, 3k and 3n.**

Into a flame-dried, 100-mL three-neck flask, containing a solution of bis(cyclopentadienyl)zirconium dichloride (496.9 mg, 1.7 mmol) in dry $Et_2O$ (10 ml) and equipped with a magnetic stirrer, a low temperature thermometer, a rubber septum and an inert gas inlet, was added dropwise a solution of *n*-butyllithium (1.6 M in hexanes, 2.13 ml, 3.4 mmol) at -78 °C under inert atmosphere. After stirring for 45 min at -78 °C, substrate **1** (1 mmol) diluted in 2 mL of dry $Et_2O$ was added dropwise to the solution at -78 °C. The resulting mixture was allowed to slowly warm-up to room temperature (over 5,5 hours if n = 1 and 12 hours if n = 3) at which time the reaction mixture is a yellow-orange suspension. Dry THF (40 mL) is then added to the reaction mixture and the resulting solution was heated at 55 °C for 3 hours. The freshly distilled first electrophile (3.0 mmol) is added dropwise after cooling the reaction mixture at 0 °C and the resulting solution was allowed to stir for 2 hours at that temperature. The second electrophile, iodine (1.269 g, 5.0 mmol) solubilized in dry THF (5 mL) is then added at 0 °C and stirred for additional 2 hours at that temperature. Finally, the reaction mixture is hydrolyzed with an aqueous solution of 1M HCl. The layers were separated and the aqueous phase was extracted with $Et_2O$ (3 times). The combined organic fractions were washed with a saturated aqueous solution of $Na_2S_2O_3$, brine, dried over $MgSO_4$, filtered and concentrated under reduced pressure. The products **3e-f**, **3k** and **3n** were isolated by silica-gel chromatography ($SiO_2$, *n*-pentane / $Et_2O$, 80:20 – 90:10).

**Typical procedure for the synthesis of 3o.**

Into a flame-dried, 100-mL three-neck flask, containing a solution of bis(cyclopentadienyl)zirconium dichloride (496.9 mg, 1.7 mmol) in dry $Et_2O$ (10 mL) and equipped with a magnetic stirrer, a low temperature thermometer, a rubber septum and an inert gas inlet, was added dropwise a solution of

*n*-butyllithium (1.6 M in hexanes, 2.13 ml, 3.4 mmol) at -78 °C under inert atmosphere. After stirring for 45 min at -78 °C, **1b** (180.3 mg, 1 mmol) diluted in 2 mL of dry $Et_2O$ was added dropwise to the solution at -78 °C. The resulting mixture was allowed to slowly warm-up to room temperature over 5,5 hours at which time the reaction mixture is a yellow-orange suspension. Dry THF (40 mL) is then added to the reaction mixture and the resulting solution was heated at 55 °C for 3 hours. Freshly distilled acetone (220 µL, 3.0 mmol) is added dropwise after cooling the reaction mixture at 0 °C and the resulting solution was allowed to stir for 2 hours at that temperature. The second electrophile, freshly distilled allyl bromide (5.0 mmol, 433 µL), is added to the solution at 0 °C, followed by the addition at that temperature of CuI (190,5 mg, 0.1 mmol) and flame-dried LiCl (8.5 mg, 0.2 mmol) brought into solution in dry THF (3 mL). The resulting mixture was allowed to stir at 55 °C for 2 hours at which time it was quenched with an aqueous solution of 1M HCl. The layers were separated and the aqueous phase was extracted with $Et_2O$ (3 times). The combined organic fractions were washed with brine, dried over $MgSO_4$, filtered and concentrated under reduced pressure. The crude was purified by silica-gel chromatography ($SiO_2$, *n*-pentane / $Et_2O$, 80:20) to afford **3o** as a unique isomer in 56% yield.

**General procedure for the synthesis of 3p-q.**

Into a flame-dried, 100-mL three-neck flask, containing a solution of bis(cyclopentadienyl)zirconium dichloride (496.9 mg, 1.7 mmol) in dry $Et_2O$ (10 mL) and equipped with a magnetic stirrer, a low temperature thermometer, a rubber septum and an inert gas inlet, was added dropwise a solution of *n*-butyllithium (1.6 M in hexane, 2.13 ml, 3.4 mmol) at -78 °C under inert atmosphere. After stirring for 45 min at -78 °C, **1b** (180.3 mg, 1 mmol) diluted in 2 mL of dry $Et_2O$ was added dropwise to the solution at -78 °C. The resulting mixture was allowed to slowly warm-up to room temperature over 5,5 hours at which time the reaction mixture is a yellow-orange suspension. Dry THF (40 mL) is then added to the reaction mixture and the resulting solution was heated at 55 °C for 3 hours. Freshly distilled acetone (220 µL, 3.0 mmol) is added dropwise after cooling the reaction mixture at 0 °C and the resulting solution was allowed to stir for 2 hours at that temperature. CuI (190,5 mg, 0.1 mmol) and flame-dried LiCl (8.5 mg, 0.2 mmol) brought into solution in dry THF (3 mL) were added at 0 °C, followed by the addition of acid chloride (2.0 mmol) at that temperature. The resulting mixture was allowed to stir at 55 °C for 3 hours at which time it was quenched with an aqueous solution of 1M HCl. The layers were separated and the aqueous phase was extracted with $Et_2O$ (3 times). The combined organic fractions were washed with brine, dried over $MgSO_4$, filtered and concentrated under reduced pressure. The crude was purified by column chromatography by silica-gel chromatography ($SiO_2$, *n*-pentane / $Et_2O$, 80:20-60:40).

**Typical procedure for the synthesis of 3r.**

Into a flame-dried, 100-mL three-neck flask, containing a solution of bis(cyclopentadienyl)zirconium dichloride (496.9 mg, 1.7 mmol) in dry $Et_2O$ (10 mL) and equipped with a magnetic stirrer, a low temperature thermometer, a rubber septum and an inert gas inlet, was added dropwise a solution of *n*-butyllithium (1.6 M in hexane, 2.13 ml, 3.4 mmol) at -78 °C under inert atmosphere. After stirring for 45 min at -78 °C, **1b** (180.3 mg, 1 mmol) diluted in 2 mL of dry $Et_2O$ was added dropwise to the solution at -78 °C. The resulting mixture was allowed to slowly warm-up to room temperature over 5,5 hours at which time the reaction mixture is a yellow-orange suspension. Dry THF (40 mL) is then added to the reaction mixture and the resulting solution was heated at 55 °C for 3 hours. Freshly distilled acetone (220 µL, 3.0 mmol) is added dropwise after cooling the reaction mixture at 0 °C and the resulting solution was allowed to stir for 2 hours at that temperature. CuI (190,5 mg, 0.1 mmol) and flame-dried LiCl (8.5 mg, 0.2 mmol) brought into solution in dry THF (3 mL) were added at 0 °C,

followed by the addition of iodobenzene (1.1 mmol, 123 µL) and $Pd(PPh_3)_4$ (115.5 mg, 0.1 mmol) solubilzed in dry THF respectively at that temperature. The resulting mixture was allowed to stir at 58 °C for 3.5 hours at which time it was quenched with an aqueous solution of 1M HCl. The layers were separated and the aqueous phase was extracted with $Et_2O$ (3 times). The combined organic fractions were washed with brine, dried over $MgSO_4$, filtered and concentrated under reduced pressure. The crude was purified by column chromatography by silica-gel chromatography ($SiO_2$, *n*-pentane / $Et_2O$, 80:20) to afford **3r** as a unique isomer in 50% yield.

**Characterization data of the products.**

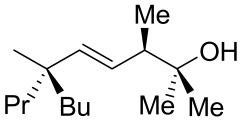


**(3*R*,6*S*,*E*)-2,3,6-trimethyl-6-propyldec-4-en-2-ol (3b).** Light yellow color oil; 83%; *E*/*Z* > 99.9; dr = 98:2; $R_f$ = 0.57 (*n*-pentane / $Et_2O$, 80:20); $^1$H NMR (300 MHz, $CDCl_3$) $\delta$0.82-0.87 (m, 6H), 0.90 (s, 3H), 0.98 (d, *J* = 6.90 Hz, 3H), 1.10 (s, 3H), 1.15 (s, 3H), 1.17-1.26 (m, 10H), 1.66 (s, 1H), 2.13 (qd, *J* = 8.50, 6.91, 6.87 Hz, 1H), 5.16 (dd, *J* = 15.76, 8.79 Hz, 1H), 5.35 (d, *J* = 15.77 Hz, 1H). $^{13}$C NMR (75 MHz, $CDCl_3$): $\delta$14.01, 14.80, 15.91, 17.22, 23.14, 23.38, 26.24, 26.39, 26.74, 38.66, 41.06, 43.70, 48.61, 72.09, 128.22, 141.82. ESHRMS for $C_{16}H_{32}NaO^+$: calcd: 263.2351. Found: 263.2349.

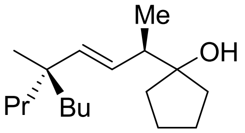


**1-((2*R*,5*S*,*E*)-5-methyl-5-propylnon-3-en-2-yl)cyclopentanol (3c).** Colorless oil; 62%; *E*/*Z* > 99.9; dr = 98:2; $R_f$ = 0.39 (*n*-pentane / $Et_2O$, 90:10); $^1$H NMR (300 MHz, $CDCl_3$) $\delta$0.83-0.89 (m, 6H), 0.91 (s, 3H), 1.03 (d, *J* = 6.88 Hz, 3H), 1.10-1.28 (m, 10H), 1.35 (s, 1H), 1.49-1.65 (m, 6H), 1.76-1.81 (m, 2H), 2.22 ( p, *J* = 6.90, 6.87 Hz, 1H), 5.25 (dd, *J* = 15.83, 7.81 Hz, 1H), 5.36 (d, *J* = 15.85 Hz, 1H). $^{13}$C NMR (75 MHz, $CDCl_3$): $\delta$14.06, 14.84, 15.72, 17.28, 23.20, 23.41, 23.99, 26.36, 37.35, 38.22, 38.63, 41.08, 43.78, 46.35, 84.04, 128.45, 141.04. ESHRMS for $C_{18}H_{35}O^+$: calcd: 267.2688. Found: 267.2692.

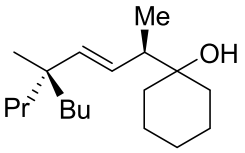


**1-((2*R*,5*S*,*E*)-5-methyl-5-propylnon-3-en-2-yl)cyclohexanol (3d).** Light yellow oil; 52%; *E*/*Z* > 99.9; dr = 98:2; $R_f$ = 0.51 (*n*-pentane / $Et_2O$, 90:10); $^1$H NMR (300 MHz, $CDCl_3$) $\delta$0.83-0.89 (m, 6H), 0.91 (s, 3H), 0.99 (d, *J* = 6.93 Hz, 3H), 1.10-1.28 (m, 11H), 1.39-1.61 (m, 10H), 2.11 (qd, *J* = 13.88, 6.91 Hz, 1H), 5.20 (dd, *J* = 15.77, 8.63 Hz, 1H), 5.33 (d, *J* = 15.79 Hz, 1H). $^{13}$C NMR (75 MHz, $CDCl_3$): $\delta$14.07, 14.84, 15.00, 17.26, 21.77, 21.86, 23.18, 23.42, 25.86, 26.42, 34.13, 35.04, 38.71, 41.11, 43.46, 47.53, 72.42, 127.97, 141.51. ESHRMS for $C_{19}H_{37}O^+$: calcd: 281.2844. Found: 281.2847.

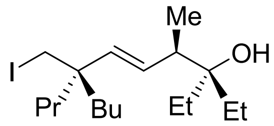


**(4*R*,7*R*,*E*)-3-ethyl-7-(iodomethyl)-4-methyl-7-propylundec-5-en-3-ol (3e).** Yellow oil; 56%; *E*/*Z* > 99.9; dr = 98:2; $R_f$ = 0.79 (*n*-pentane / $Et_2O$, 80:20); $^1$H NMR (300 MHz, $CDCl_3$) $\delta$0.83-0.92(m, 12H), 0.99 (d, *J* = 6.94 Hz, 3H), 1.09-1.25 (m, 5H), 1.26-1.30 (m, 2H), 1.32-1.42 (m, 5H), 1.44-1.55 (m, 4H),

2.31 (td, *J* = 15.79, 7.26, Hz, 1H), 3.19 (s, 1H), 5.22 (d, *J* = 16.03 Hz, 1H), 5.33 (dd, *J* = 16.01, 8.33 Hz, 1H). $^{13}$C NMR (75 MHz, $CDCl_3$): $\delta$7.46, 7.59, 14.00, 14.59, 15.06, 16.91, 20.51, 23.14, 26.10, 27.96, 28.47, 36.61, 38.86, 41.40, 43.79, 75.41, 131.58, 136.94. ESHRMS for $C_{18}H_{36}IO^+$: calcd: 395.1811. Found: 395.1815.

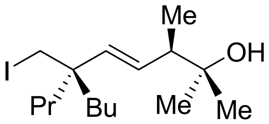


**(3*R*,6*R*,*E*)-6-(iodomethyl)-2,3-dimethyl-6-propyldec-4-en-2-ol (3f).** Yellow oil; 51%; *E/Z* > 99.9; dr = 98:2; $R_f$ = 0.44 (*n*-pentane / $Et_2O$, 80:20); $^{1}$H NMR (300 MHz, $CDCl_3$) $\delta$0.84-0.90 (m, 6H), 0.99 (d, *J* = 6.92 Hz, 3H), 1.06-1.11 (m, 2H), 1.13 (s, 3H), 1.16 (s, 3H), 1.17-1.43 (m, 8H), 1.75 (bs, 1H), 2.15 (tq, *J* = 6.87, 4.05 Hz, 1H), 3.14 (d, *J* = 9.87 Hz, 1H), 3.18 (d, *J* = 9.88 Hz, 1H), 5.22-5.24 (m, 2H*)*. $^{13}$C NMR (75 MHz, $CDCl_3$): $\delta$13.98, 14.59, 15.91, 16.82, 20.58, 23.12, 26.15, 26.19, 27.08, 36.54, 38.47, 41.42, 48.69, 72.22, 131.54, 137.80. ESHRMS for $C_{16}H_{32}IO^+$: calcd: 367.1498. Found: 367.1495.

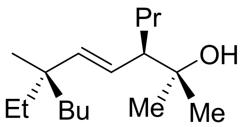


**(3*R*,6*S*,*E*)-6-ethyl-2,6-dimethyl-3-propyldec-4-en-2-ol (3g).** Yellow oil; 62%; *E/Z* > 99.9; dr = 98:2; $R_f$ = 0.37 (*n*-pentane / $Et_2O$, 90:10); $^{1}$H NMR (300 MHz, $CDCl_3$) $\delta$ $^{1}$H NMR (300 MHz, $CDCl_3$) δ 0.76 (t, *J* = 7.5 Hz, 3H), 0.86 (td, *J* = 7.1, 2.8 Hz, 6H), 1.10 (s, 3H), 0.91 (s, 3H), 1.12 – 1.16 (m, 2H), 1.17 (s, 3H), 1.19 – 1.53 (m, 10H), 1.72 (s, 1H), 1.84 – 2.01 (m, 1H), 5.00 (dd, *J* = 15.7, 9.6 Hz, 1H), 5.35 (d, *J* = 15.7 Hz, 1H). ). $^{13}$C NMR (75 MHz, $CDCl_3$): 8.49, 13.90, 14.02, 21.10, 22.48, 23.38, 26.51, 26.55, 26.85, 31.71, 33.51, 39.13, 40.83, 55.02, 71.86, 127.16, 143.82. ESHRMS for $C_{17}H_{34}NaO^+$: calcd: 277.2507.

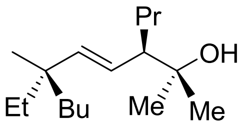


**(3*R*,6*S*,*E*)-6-ethyl-2,6-dimethyl-3-propyldec-4-en-2-ol (3h).** Yellow oil; 61%; *E/Z* > 99.9; dr = 98:2; $R_f$ = 0.60 (*n*-pentane / $Et_2O$, 90:10); $^{1}$H NMR (300 MHz, $CDCl_3$) $\delta$0.75 (t, *J* = 7.46 Hz, 3H), 0.83-0.88 (m, 6H), 0.90 (s, 3H), 1.09 (s, 3H), 1.15-1.10 (m, 2H), 1.16 (s, 3H), 1.17-1.52 (m, 10H), 1.75 (bs, 1H), 1.86-1.96 (m, 1H), 4.99 (dd, *J* = 15.74, 9.63 Hz, 1H), 5.34 (d, *J* = 15.74 Hz, 1H). $^{13}$C NMR (75 MHz, $CDCl_3$): $\delta$8.48, 13.89, 14.01, 21.09, 22.50, 23.38, 26.51, 26.63, 26.81, 31.68, 33.51, 39.12, 40.83, 55.01, 71.87, 127.19, 143.73. ESHRMS for $C_{17}H_{34}NaO^+$: calcd: 277.2507. Found: 277.2508.

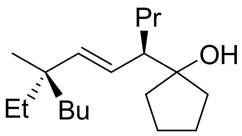


**1-((4*R*,7*S*,*E*)-7-ethyl-7-methylundec-5-en-4-yl)cyclopentanol (3i)**. Yellow oil; 61%; *E/Z* > 99.9; dr = 97:3; $R_f$ = 0.80 (*n*-pentane / $Et_2O$, 90:10); $^{1}$H NMR (300 MHz, $CDCl_3$) $\delta$0.76 (t, *J* = 7.47 Hz, 3H), 0.83-0.98 (m, 6H), 0.90 (s, 3H), 1.02-1.34 (m, 10H), 1.39-1.62 (m, 9H), 1.71-1.80 (m, 2H), 2.00 (ddd, *J* = 11.94, 9.53, 2.52 Hz, 1H), 5.06 (dd, *J* = 15.76, 9.41 Hz, 1H), 5.30 (d, *J* = 15.77 Hz, 1H). ). $^{13}$C NMR (75 MHz, $CDCl_3$): $\delta$8.51, 13.94, 14.04, 20.88, 22.57, 23.42, 23.94, 23.99, 26.45, 31.77, 33.58, 37.65, 38.05, 39.02, 40.82, 52.74, 84.14, 127.49, 142.49. ESHRMS for $C_{19}H_{37}O^+$: calcd: 281.2844. Found: 281.2840.

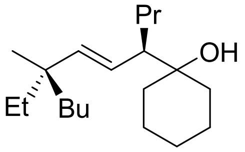


**1-((4*R*,7*S*,E)-7-ethyl-7-methylundec-5-en-4-yl)cyclohexanol (3j).** Yellow oil; 62%; *E*/*Z* > 99.9; dr = 98:2; $R_f$ = 0.80 (*n*-pentane / $Et_2O$, 90:10); $^1$H NMR (300 MHz, $CDCl_3$) $\delta$ 0.76 (t, *J* = 7.45, 3H), 0.86 (t, *J* = 6.95 Hz, 6H), 0.90 (s, 3H), 1.02-1.33 (m, 12H), 1.36-1.61 (m, 1H), 1.71-1.95 (m, 11H), 5.02 (dd, *J* = 15.76, 9.63 Hz, 1H), 5.29 (d, *J* = 15.76 Hz, 1H). ). $^{13}$C NMR (75 MHz, $CDCl_3$): $\delta$ 8.52, 13.94, 14.05, 21.08, 21.74, 21.85, 22.60, 23.42, 25.93, 26.52, 30.63, 33.55, 34.47, 34.93, 39.12, 40.83, 54.21, 72.44, 126.89, 143.22. ESHRMS for $C_{20}H_{39}O^+$: calcd: 295.3001. Found: 295.3002.

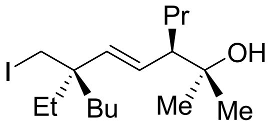


**(3*R*,6*R*,*E*)-6-ethyl-6-(iodomethyl)-2-methyl-3-propyldec-4-en-2-ol (3k).** Yellow oil; 55%; *E*/*Z* > 99.9; dr = 96:4; $R_f$ = 0.60 (*n*-pentane / $Et_2O$, 80:20); $^1$H NMR (300 MHz, $CDCl_3$) $\delta$ 0.77 (t, *J* = 7.44 Hz, 1H), 0.84-0.90 (m, 6H), 1.05-1.12 (m, 1H), 1.13 (s, 3H), 1.14-1.17 (m, 1H), 1.18 (s, 3H), 1.19-1.55 (m, 10H), 1.87-2.02 (m, 2H), 3.15 (d, *J* = 9.90 Hz, 1H), 3.22 (d, *J* = 9.91 Hz, 1H), 5.07 (dd, *J* = 15.92, 9.50 Hz, 1H), 5.26 (d, *J* = 15.94 Hz, 1H). $^{13}$C NMR (75 MHz, $CDCl_3$): $\delta$ 7.88, 13.89, 13.96, 19.89, 21.18, 23.12, 26.40, 26.49, 27.07, 28.35, 31.70, 36.15, 41.89, 55.05, 72.06, 130.46, 139.78. ESHRMS for $C_{17}H_{34}IO^+$: calcd: 381.1654. Found: 381.1659.

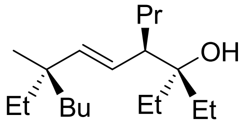


**(4*R*,7*R*,*E*)-3,7-diethyl-7-(iodomethyl)-4-propylundec-5-en-3-ol (3l).** Yellow oil; 59%; *E*/*Z* > 99.9; dr = 98:2; $R_f$ = 0.73 (*n*-pentane / $Et_2O$, 90:10); $^1$H NMR (300 MHz, $CDCl_3$) $\delta$ 0.76 (t, *J* = 7.46 Hz, 3H), 0.89-0.81 (m, 12H), 0.91 (s, 3H), 1.05-1.35 (m, 11H), 1.37-1.63 (m, 6H), 2.00-2.12 (m, 1H), 5.05 (dd, *J* = 15.76, 9.59 Hz, 1H), 5.30 (d, *J* = 15.77 Hz, 1H). $^{13}$C NMR (75 MHz, $CDCl_3$): $\delta$ 7.34, 7.48, 8.51, 13.92, 14.05, 21.10, 22.50, 23.43, 26.52, 28.26, 28.66, 30.75, 33.62, 39.14, 40.85, 50.11, 75.14, 127.08, 142.95. ESHRMS for $C_{19}H_{39}O^+$: calcd: 283.3001. Found: 283.2997.

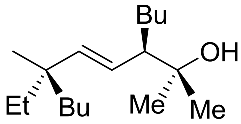


**(3*R*,6*S*,*E*)-3-butyl-6-ethyl-2,6-dimethyldec-4-en-2-ol (3m).** Yellow oil; 50%; *E*/*Z* > 99.9; dr = 98:2; $R_f$ = 0.43 (*n*-pentane / $Et_2O$, 90:10); $^1$H NMR (300 MHz, $CDCl_3$) $\delta$ 0.77 (t, *J* = 7.5 Hz, 3H), 0.87 (t, *J* = 7.0 Hz, 6H), 0.92 (s, 3H), 1.11 (s, 3H), 1.18 (s, 3H), 1.19-1.35 (m, 13H), 1.71 (bs, 1H), 1.85– 1.96 (m, 13H), 5.01 (dd, *J* = 15.8, 9.6 Hz, 1H), 5.35 (d, *J* = 15.7 Hz, 1H). $^{13}$C NMR (75 MHz, $CDCl_3$) $\delta$ 8.48, 13.98, 14.04, 22.43, 22.54, 23.39, 26.54, 26.87, 29.13, 30.29, 33.45, 39.14, 40.87, 55.21, 71.92, 127.20, 143.87.

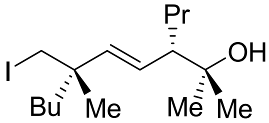


**(3*S*,6*S*,*E*)-6-(iodomethyl)-2,6-dimethyl-3-propyldec-4-en-2-ol ((3*S**,6*S**)-3n).** Orange oil; 57%; *E*/*Z* > 99.9; dr = 95:5; $R_f$ = 0.61 (*n*-pentane / $Et_2O$, 80:20); $^1$H NMR (300 MHz, $CDCl_3$) $\delta$ 0.83-0.89 (m, 6H), 1.09 (s, 3H), 1.12 (s, 3H), 1.16 (s, 3H), 1.17-1.52 (m, 10H), 1.79 (bs, 1H), 1.89-1.97 (m, 1H), 3.12-3.19 (m, 2H), 5.11 (dd, *J* = 15.76, 9.56 Hz, 1H), 5.33 (d, *J* = 15.76 Hz, 1H). $^{13}$C NMR (75 MHz, $CDCl_3$) $\delta$ 13.96,

13.97, 21.13, 22.62, 23.13, 24.34, 26.81, 26.84, 26.96, 31.54, 39.18, 39.62, 54.75, 72.05, 129.69, 139.90. ESHRMS for $C_{16}H_{31}INaO^+$: calcd: 389.1317. Found: 389.1316.

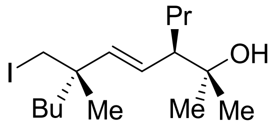

**(3*R*,6*S*,*E*)-6-(iodomethyl)-2,6-dimethyl-3-propyldec-4-en-2-ol ((3*R**,6*S**)-3n).** Yellow oil; 52%; *E*/*Z* > 99.9; dr = 95:5; $R_f$ = 0.60 (*n*-pentane / $Et_2O$, 80:20); $^1$H NMR (300 MHz, $CDCl_3$) $\delta$ 0.81-0.84 (m, 6H), 1.04 (s, 3H), 1.09 (s, 3H), 1.14 (s, 3H), 1.15-1.49 (m, 10H), 1.97-1.85 (m, 1H), 2.00 (bs, 1H), 3.14 (s, 2H), 5.05 (dd, *J* = 15.68, 9.63 Hz, 1H), 5.26 (d, *J* = 15.69 Hz, 1H). $^{13}$C NMR (75 MHz, $CDCl_3$): $\delta$ 13.88, 13.94, 21.16, 23.15, 23.31, 23.74, 26.33, 27.10, 27.37, 31.72, 39.80, 39.83, 54.76, 72.06, 130.03, 140.45. ESHRMS for $C_{16}H_{31}INaO^+$: calcd: 389.1317. Found: 389.1312.

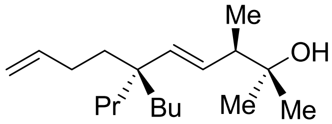

**(3*R*,6*S*,*E*)-6-butyl-2-methyl-3,6-dipropyldeca-4,9-dien-2-ol (3o).** Light yellow oil; 56%; *E*/*Z* > 99.9; dr = 98:2; $R_f$ = 0.545 (*n*-pentane / $Et_2O$, 80:20); $^1$H NMR (300 MHz, $CDCl_3$) $\delta$ 0.84-0.90 (m, 6H), 1.01 (d, *J* = 6.92 Hz, 3H), 1.03-1.12 (m, 2H), 1.13 (s, 3H), 1.17 (s, 3H), 1.17-1.41 (m, 10H), 1.59 (s, 9H), 1.83-1.93 (m, 2H), 2.16 (qd, *J* = 8.24, 6.92, 6.92, 6.87 Hz, 1H), 4.86-5.02 (m, 2H), 5.18 (dd, *J* = 15.89, 8.37 Hz, 1H), 5.29 (d, *J* = 15.90 Hz, 1H), 5.72-5.86 (m, 1H). $^{13}$C NMR (75 MHz, $CDCl_3$) $\delta$ 14.06, 14.81, 16.04, 16.65, 23.70, 25.62, 26.41, 26.80, 28.05, 36.07, 36.26, 39.23, 41.05, 48.87, 72.17, 113.73, 129.30, 139.42, 141.18. ESHRMS for $C_{19}H_{37}O^+$: calcd: 281.2844. Found: 281.2844.

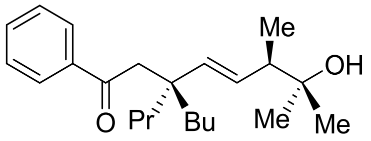

**(3*S*,6*R*,*E*)-3-butyl-7-hydroxy-6,7-dimethyl-1-phenyl-3-propyloct-4-en-1-one (3p).** Yellow oil; 54%; *E*/*Z* > 99.9; dr = 98:2; $R_f$ = 0.545 (*n*-pentane / $Et_2O$, 60:40); $^1$H NMR (300 MHz, $CDCl_3$) ⍰ 0.80-0.89 (m, 6H), 0.93 (d, *J* = 6.92 Hz, 3H), 1.10 (s, 3H), 1.16 (s, 3H), 1.17-1.30 (m, 6H), 1.42-1.52 (m, 1H), 2.06-2.17 (m, 1H), 2.40 (bs, 1H), 2.84 (d, *J* = 15.37 Hz, 1H), 3.09 (d, *J* = 15.37 Hz, 1H), 5.19 (dd, *J* = 15.87, 8.94 Hz, 1H), 5.46 (d, *J* = 15.88 Hz, 1H), 7.37-7.48 (m, 2H), 7.49-7.55 (m, 1H), 7.87-7.91 (m, 2H). $^{13}$C NMR (75 MHz, $CDCl_3$) $\delta$ 14.02, 14.62, 15.90, 16.96, 23.20, 25.44, 25.71, 27.23, 37.22, 38.25, 41.79, 44.80, 48.68, 72.18, 127.95, 128.35, 129.20, 132.66, 138.38, 140.21, 199.85. ESHRMS for $C_{23}H_{36}NaO_2^+$: calcd: 367.2613. Found: 367.2615.

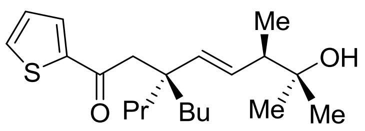

**(3*S*,6*R*,*E*)-3-butyl-7-hydroxy-6,7-dimethyl-3-propyl-1-(thiophen-2-yl)oct-4-en-1-one (3q).** Yellow oil; 53%; *E*/*Z* > 99.9; dr = 98:2; $R_f$ = 0.40 (*n*-pentane / $Et_2O$, 80:20); $^1$H NMR (300 MHz, $CDCl_3$) $\delta$ 0.83-0.94 (m, 6H), 1.09 (s, 3H), 1.15 (s, 3H), 1.18-1.31 (m, 6H), 1.38-1.55 (m, 4H), 2.07-2.17 (m, 1H), 2.32 (bs, 1H), 2.77 (d, *J* = 14.73 Hz, 1H), 2.97 (d, *J* = 14.72 Hz, 1H), 5.21 (dd, *J* = 15.86, 8.93 Hz, 1H), 5.47 (d, *J* = 15.91 Hz, 1H), 7.08-7.11 (m, 1H), 7.60 (dd, *J* = 4.94, 0.98 Hz, 1H), 7.66 (dd, *J* = 3.76, 1.05 Hz, 1H). $^{13}$C NMR (75 MHz, $CDCl_3$) $\delta$ 14.04, 14.64, 15.90, 16.96, 23.20, 25.46, 25.71, 27.20, 37.10, 38.28, 42.04, 46.16, 48.70, 72.18, 127.89, 129.46, 131.73, 133.60, 139.91, 146.01, 192.33. ESHRMS for $C_{21}H_{35}O_2S^+$: calcd: 351.2358. Found: 351.2362.

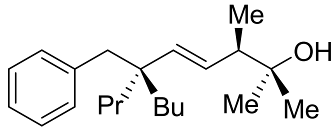


**(3*R*,6*S*,*E*)-6-benzyl-2,3-dimethyl-6-propyldec-4-en-2-ol (3r).** Orange oil; 50%; *E*/*Z* > 99.9; dr = 98:2; $R_f$ = 0.52 (*n*-pentane / $Et_2O$, 80:20); $^1H$ NMR (300 MHz, $CDCl_3$) $\delta$ 0.87-0.92 (m, 6H), 0.95 (d, *J* = 6.92 Hz, 3H), 1.01 (s, 3H), 1.11 (s, 3H), 1.19-1.31 (m, 10H), 1.46 (s, 1H), 2.08-2.20 (m, 1H), 2.56 (d, *J* = 13.24 Hz, 1H), 2.61 (d, *J* = 13.23 Hz, 1H), 4.99 (dd, *J* = 15.87, 9.00 Hz, 1H), 5.35 (d, *J* = 15.88 Hz, 1H), 7.08-7.01 (m, 2H), 7.12-7.25 (m, 3H). $^{13}C$ NMR (75 MHz, $CDCl_3$) $\delta$ 14.15, 14.71, 16.02, 16.95, 23.32, 25.86, 25.94, 26.95, 35.73, 38.00, 42.82, 44.34, 48.98, 72.18, 125.74, 127.50, 129.29, 130.37, 138.71, 140.65. ESHRMS for $C_{22}H_{36}NaO^+$: calcd: 339.2664. Found: 339.2665.

**Pathways considered for the computational study**

Gibbs Free energy in Kcal.mol$^{-1}$

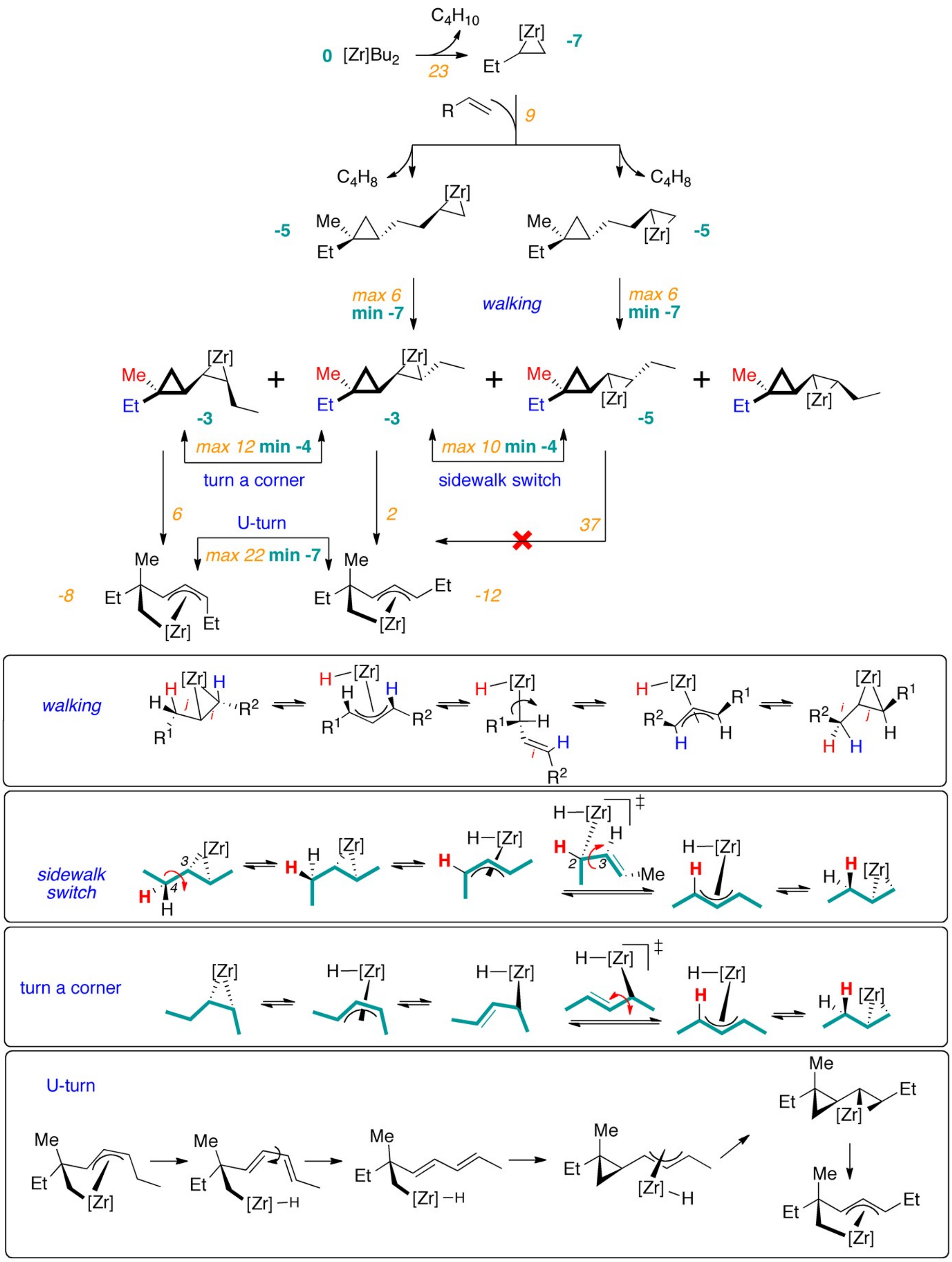

**Computational details**

The electronic structure calculations required for the depiction of minima and transition states molecular structures were performed at the density functional theory (DFT) level using the M06[4] hybrid functional as implemented in the Gaussian program code.[5] This density functional was shown to perform well for computing activation energies of reaction that involve zirconocene.[6] Zirconium atom was described by the corresponding scalar relativistic pseudopotentials of the Stuttgart-Dresden-Koln type[7] in association with its valence extended basis set.[8] For the rest atoms the polarized all-electron triple zeta basis set 6-311G(d,p) was used.[9] Optimization were carried without any geometry constraint. Enthalpy energies were obtained at T = 298.15K based on the harmonic approximation. Solvation by diethylether has been taken into account in all calculations, including optimizations and frequency calculation, by the SMD implicit solvent model.[10] Intrinsic Reaction Paths (IRPs) were traced from the various transition structures to verify the reactant to product linkage.[11]

**Cartesian Coordinates**

**$Cp_2ZrBu_2$**

47

E: -749.479981746 ; H: -749.050430 ; G: -749.123732

| | | | |
|---|---|---|---|
| Zr | 0.11628007 | 0.16524502 | 0.06203976 |
| C | 0.89993457 | 2.25053996 | 1.35087888 |
| C | -0.29850926 | 1.80193542 | 1.94191290 |
| C | 1.89337420 | 1.27836304 | 1.58637768 |
| H | 1.03012923 | 3.16890527 | 0.79245948 |
| C | -0.04318212 | 0.54513580 | 2.54237095 |
| H | -1.24461382 | 2.32812002 | 1.94354949 |
| C | 1.31433200 | 0.22923341 | 2.32612946 |
| H | 2.91467786 | 1.31233130 | 1.22643656 |
| H | -0.75753592 | -0.05808333 | 3.08860008 |
| H | 1.81801308 | -0.66990866 | 2.65552098 |
| C | -1.43239956 | -0.96677707 | -1.65693018 |
| C | -1.69959986 | -1.56061537 | -0.40457861 |
| C | -1.82835539 | 0.38387510 | -1.59568319 |
| H | -0.97287324 | -1.45374724 | -2.50856859 |
| C | -2.25928955 | -0.57430142 | 0.43371988 |
| H | -1.49733168 | -2.58828519 | -0.13091816 |
| C | -2.33811666 | 0.63048987 | -0.30412881 |
| H | -1.74146055 | 1.10970905 | -2.39399064 |
| H | -2.58157499 | -0.71566592 | 1.45757819 |
| H | -2.73137798 | 1.57347677 | 0.05384329 |
| C | 1.09197195 | 1.45578794 | -1.61477995 |
| C | 1.26952723 | 0.83136929 | -2.99291707 |
| H | 2.08906983 | 1.71426695 | -1.21828911 |
| H | 0.55645306 | 2.41657130 | -1.70247373 |

| | | | |
|---|---|---|---|
| C | 2.12189477 | 1.65978587 | -3.94956646 |
| H | 1.73426117 | -0.16578070 | -2.90516040 |
| H | 0.29163198 | 0.65452840 | -3.46876059 |
| C | 2.28852521 | 1.01223491 | -5.31161819 |
| H | 1.66785738 | 2.65586726 | -4.05856876 |
| H | 3.10833389 | 1.82790865 | -3.49307047 |
| H | 2.90992281 | 1.61596294 | -5.98186921 |
| H | 2.76063787 | 0.02574542 | -5.22448815 |
| H | 1.31847203 | 0.86712541 | -5.80311979 |
| C | 1.39774505 | -1.80025531 | 0.08250769 |
| C | 1.41545232 | -2.75429382 | -1.10363242 |
| H | 1.15189648 | -2.36218162 | 1.00016717 |
| H | 2.42299829 | -1.41747237 | 0.24460105 |
| C | 2.43817979 | -3.88027964 | -0.98565557 |
| H | 0.42345962 | -3.21139414 | -1.24839690 |
| H | 1.62142717 | -2.20784758 | -2.03953908 |
| C | 2.43989991 | -4.80575297 | -2.18791795 |
| H | 3.43797420 | -3.44276865 | -0.84847348 |
| H | 2.23280064 | -4.45413401 | -0.06957106 |
| H | 3.17738830 | -5.60975918 | -2.08996076 |
| H | 1.45772775 | -5.27443669 | -2.32793747 |
| H | 2.67277487 | -4.25615047 | -3.10839576 |

**butane**

14

E: -158.361764616 ; H: -158.224513 ; G: -158.259004

| | | | |
|---|---|---|---|
| C | -2.21343556 | 1.96738637 | 0.01743523 |
| H | -1.87842415 | 2.45191446 | -0.91134515 |
| H | -3.31181259 | 1.94577608 | -0.03399793 |
| C | -1.76941178 | 2.78467180 | 1.21459581 |
| H | -0.67557696 | 2.84449539 | 1.26787949 |
| H | -2.15437534 | 3.80902971 | 1.17858442 |
| H | -2.11754815 | 2.33374644 | 2.15185916 |
| C | -1.68898254 | 0.54168919 | 0.05155598 |
| H | -2.02395494 | 0.05719975 | 0.98037149 |
| H | -0.59060351 | 0.56332207 | 0.10296486 |
| C | -2.13301253 | -0.27569034 | -1.14554218 |
| H | -1.74800872 | -1.30003112 | -1.10946636 |
| H | -3.22684596 | -0.33556800 | -1.19879118 |
| H | -1.78491827 | 0.17517420 | -2.08284961 |

**but-1-ene**

12

E: -157.127678195 ; H: -157.014278 ; G: -157.047991

| | | | |
|---|---|---|---|
| C | -2.50685551 | 1.98069181 | 0.15561964 |

| | | | |
|---|---|---|---|
| H | -2.48796825 | 2.41721250 | -0.85133822 |
| H | -3.54220023 | 2.06639162 | 0.51562440 |
| C | -2.12701590 | 0.53949562 | 0.08146342 |
| H | -2.14153110 | -0.00403528 | 1.02922619 |
| C | -1.76126850 | -0.09617598 | -1.02273940 |
| H | -1.72980215 | 0.41334062 | -1.98461188 |
| H | -1.48219073 | -1.14600173 | -1.01610760 |
| C | -1.59167925 | 2.76213669 | 1.08818791 |
| H | -0.55400146 | 2.72637710 | 0.73832455 |
| H | -1.88918070 | 3.81328965 | 1.15507708 |
| H | -1.61238722 | 2.34515338 | 2.10180891 |

**Substrate**

28

E: -391.648071616 ; H: -391.381740 ; G: -391.432473

| | | | |
|---|---|---|---|
| C | -1.999285 | 0.568473 | -0.035726 |
| C | -0.497892 | 0.498087 | -0.027494 |
| C | -1.190909 | 1.833678 | 0.012478 |
| H | -0.070425 | 0.171543 | 0.922883 |
| H | -1.177680 | 2.404649 | 0.937210 |
| H | -1.144207 | 2.434528 | -0.893946 |
| C | -2.757435 | 0.236346 | -1.295711 |
| H | -2.764764 | -0.843008 | -1.494568 |
| H | -3.802387 | 0.562114 | -1.209955 |
| H | -2.336732 | 0.729789 | -2.176406 |
| C | -2.688439 | 0.144233 | 1.242659 |
| H | -3.664130 | 0.648890 | 1.313002 |
| H | -2.096838 | 0.499704 | 2.097528 |
| C | -2.877894 | -1.358996 | 1.348028 |
| H | -3.305073 | -1.643425 | 2.315079 |
| H | -3.550244 | -1.738501 | 0.570617 |
| H | -1.919152 | -1.881873 | 1.240522 |
| C | 0.323023 | 0.101102 | -1.223356 |
| H | 0.373316 | -0.995106 | -1.304257 |
| H | -0.152433 | 0.454479 | -2.148311 |
| C | 1.741437 | 0.663525 | -1.150980 |
| H | 2.235035 | 0.312016 | -0.234136 |
| H | 1.673124 | 1.759612 | -1.071848 |
| C | 2.563861 | 0.294168 | -2.338689 |
| H | 2.181129 | 0.628936 | -3.305681 |
| C | 3.685104 | -0.412181 | -2.297601 |
| H | 4.239667 | -0.663884 | -3.197256 |
| H | 4.095705 | -0.765779 | -1.353081 |

**I**

49
E: -825.627343725 ; H: -825.187319 ; G: -825.260798

| | | | |
|---|---|---|---|
| C | -2.592489 | 0.872411 | 0.156363 |
| C | -1.451276 | 1.796102 | -0.221183 |
| C | -2.861719 | 2.108657 | -0.649829 |
| H | -1.090528 | 2.395875 | 0.615473 |
| H | -3.390395 | 2.930293 | -0.172173 |
| H | -3.085960 | 1.982202 | -1.707674 |
| C | -2.690851 | -0.470411 | -0.513587 |
| H | -2.118360 | -1.225474 | 0.043028 |
| H | -3.729074 | -0.818454 | -0.571783 |
| H | -2.295803 | -0.445746 | -1.533198 |
| C | -3.044482 | 0.905650 | 1.599421 |
| H | -2.844924 | 1.906753 | 2.006582 |
| H | -2.430702 | 0.206195 | 2.187324 |
| C | -4.516357 | 0.573377 | 1.780241 |
| H | -4.832462 | 0.702929 | 2.820570 |
| H | -5.144354 | 1.223928 | 1.158601 |
| H | -4.736732 | -0.462564 | 1.500080 |
| C | -0.439519 | 1.451900 | -1.258439 |
| H | -0.868059 | 1.273001 | -2.247362 |
| C | 0.713890 | 0.669945 | -0.986352 |
| H | 1.014590 | 0.553714 | 0.059908 |
| C | 2.881321 | -0.469514 | -1.602807 |
| H | 2.761188 | -1.550514 | -1.759333 |
| H | 3.727456 | -0.148480 | -2.218680 |
| H | 3.159429 | -0.324815 | -0.552355 |
| C | 1.629308 | 0.275537 | -1.935465 |
| H | 2.960826 | 2.280897 | -2.736512 |
| H | 1.310946 | 0.253209 | -2.980539 |
| Zr | 1.358372 | 2.921345 | -2.008314 |
| C | 1.418770 | 4.570339 | -3.931790 |
| C | 1.381941 | 3.301999 | -4.548063 |
| C | 0.182604 | 4.772968 | -3.272496 |
| H | 2.251773 | 5.261934 | -3.956331 |
| C | 0.135571 | 2.715568 | -4.256358 |
| H | 2.176601 | 2.855989 | -5.130033 |
| C | -0.608178 | 3.625507 | -3.466901 |
| H | -0.103533 | 5.653251 | -2.710284 |
| H | -0.193465 | 1.733664 | -4.574875 |
| H | -1.608844 | 3.470996 | -3.083662 |
| C | 1.555030 | 3.278393 | 0.526801 |
| C | 2.854368 | 2.962740 | 0.079803 |
| C | 1.176089 | 4.506212 | -0.050339 |
| H | 0.949941 | 2.679033 | 1.193695 |

| | | | |
|---|---|---|---|
| C | 3.292105 | 4.006728 | -0.765109 |
| H | 3.417197 | 2.071324 | 0.327315 |
| C | 2.256409 | 4.959342 | -0.846005 |
| H | 0.227347 | 5.011237 | 0.090182 |
| H | 4.247991 | 4.057161 | -1.268405 |
| H | 2.283661 | 5.879601 | -1.415548 |

**A**

49

E: -825.623315064 ; H: -825.179701 ; G: -825.253689

| | | | |
|---|---|---|---|
| C | -2.14066920 | 0.11652997 | -0.02664652 |
| C | -0.95228189 | 1.05872794 | -0.06395357 |
| C | -2.35568139 | 1.60309342 | -0.11843091 |
| H | -0.47693454 | 1.15724923 | 0.91123834 |
| H | -2.76223151 | 2.12063355 | 0.74890778 |
| H | -2.68302159 | 2.00462590 | -1.07717140 |
| C | -2.45340699 | -0.74159915 | -1.22246914 |
| H | -1.82443967 | -1.64326093 | -1.23300709 |
| H | -3.50049098 | -1.06789482 | -1.21744485 |
| H | -2.28967353 | -0.21628759 | -2.16770058 |
| C | -2.46058122 | -0.52725844 | 1.30561974 |
| H | -2.05737836 | 0.10487778 | 2.10948851 |
| H | -1.92950603 | -1.48933808 | 1.37785005 |
| C | -3.94652884 | -0.74565085 | 1.54310211 |
| H | -4.13786286 | -1.13839876 | 2.54732194 |
| H | -4.50136032 | 0.19549492 | 1.44338916 |
| H | -4.37612948 | -1.45797185 | 0.83011869 |
| C | -0.03486722 | 1.18538076 | -1.23659526 |
| H | -0.55109680 | 0.89471900 | -2.15358640 |
| C | 1.37826479 | 0.75094119 | -1.20223170 |
| Zr | 1.28512014 | 2.98108623 | -1.82053727 |
| C | 1.80728352 | 4.06471223 | -4.06966688 |
| C | 2.00045008 | 2.68538207 | -4.30379860 |
| C | 0.43137380 | 4.26703374 | -3.80272884 |
| H | 2.57207542 | 4.83201556 | -4.09330237 |
| C | 0.76052149 | 2.03837296 | -4.16916063 |
| H | 2.94730032 | 2.20135256 | -4.51427915 |
| C | -0.21703821 | 3.01303045 | -3.86731302 |
| H | -0.04319378 | 5.21882932 | -3.59623768 |
| H | 0.58915088 | 0.97373819 | -4.26711843 |
| H | -1.27364270 | 2.82948419 | -3.71731831 |
| C | 1.07743672 | 3.55110329 | 0.68761718 |
| C | 2.45010590 | 3.55349485 | 0.35426471 |
| C | 0.45210005 | 4.61678807 | 0.01279324 |
| H | 0.58709499 | 2.85909873 | 1.35752907 |

| | | | |
|---|---|---|---|
| C | 2.67270223 | 4.63170583 | -0.53911202 |
| H | 3.19888430 | 2.86852131 | 0.73189559 |
| C | 1.43424354 | 5.28101410 | -0.75526808 |
| H | -0.60406908 | 4.85855967 | 0.05118645 |
| H | 3.62335689 | 4.91715119 | -0.97449569 |
| H | 1.27060173 | 6.13916306 | -1.39572611 |
| C | 1.98085146 | 0.20753196 | 0.07783414 |
| H | 3.07938015 | 0.21617775 | 0.00828012 |
| H | 1.74206518 | 0.85514244 | 0.93281423 |
| C | 1.52626769 | -1.21399694 | 0.39256633 |
| H | 1.78664912 | -1.89584115 | -0.42687397 |
| H | 1.98873128 | -1.60372344 | 1.30838553 |
| H | 0.43776060 | -1.26048742 | 0.52072796 |
| H | 1.66255862 | 0.11363235 | -2.05088831 |

**B**

49

E: -825.623650641 ; H: -825.180197 ; G: -825.253665

| | | | |
|---|---|---|---|
| C | -2.179063 | 0.144138 | -0.073397 |
| C | -0.848457 | 0.864412 | -0.086494 |
| C | -2.128343 | 1.630545 | 0.141603 |
| H | -0.271128 | 0.694098 | 0.824145 |
| H | -2.343775 | 2.022115 | 1.134898 |
| H | -2.462008 | 2.266083 | -0.679143 |
| C | -2.762131 | -0.364667 | -1.363926 |
| H | -2.344191 | -1.345516 | -1.629350 |
| H | -3.850957 | -0.476094 | -1.291698 |
| H | -2.568957 | 0.315007 | -2.200176 |
| C | -2.479213 | -0.710102 | 1.139894 |
| H | -1.924473 | -0.304884 | 1.997709 |
| H | -2.082837 | -1.723430 | 0.972944 |
| C | -3.954726 | -0.790357 | 1.495368 |
| H | -4.108490 | -1.330539 | 2.435616 |
| H | -4.385125 | 0.211981 | 1.613988 |
| H | -4.535340 | -1.310344 | 0.725754 |
| C | 0.003448 | 1.038234 | -1.308449 |
| H | -0.512022 | 0.696434 | -2.210389 |
| C | 1.425320 | 0.653719 | -1.299152 |
| H | 1.829322 | 0.577052 | -0.279033 |
| C | 3.456376 | -0.485332 | -2.295921 |
| H | 3.942327 | -0.521412 | -1.312406 |
| H | 3.821967 | -1.341842 | -2.874136 |
| H | 3.806259 | 0.428579 | -2.795496 |
| C | 1.943893 | -0.485656 | -2.153629 |
| H | 1.484189 | -0.455450 | -3.151243 |

| | | | |
|---|---|---|---|
| H | 1.632641 | -1.455335 | -1.724983 |
| Zr | 1.266987 | 2.881395 | -1.883890 |
| C | 1.801725 | 3.960968 | -4.118700 |
| C | 1.930073 | 2.576351 | -4.371313 |
| C | 0.438108 | 4.223585 | -3.838898 |
| H | 2.598829 | 4.694579 | -4.141719 |
| C | 0.658880 | 1.990089 | -4.240841 |
| H | 2.847942 | 2.049617 | -4.606358 |
| C | -0.269167 | 3.001560 | -3.915668 |
| H | 0.009832 | 5.193862 | -3.617865 |
| H | 0.431923 | 0.939710 | -4.363846 |
| H | -1.330982 | 2.860660 | -3.750308 |
| C | 1.107146 | 3.306362 | 0.649593 |
| C | 2.469537 | 3.399188 | 0.297621 |
| C | 0.418857 | 4.378005 | 0.045850 |
| H | 0.663021 | 2.546701 | 1.277313 |
| C | 2.624478 | 4.529858 | -0.543830 |
| H | 3.254301 | 2.722037 | 0.611647 |
| C | 1.354556 | 5.136011 | -0.688554 |
| H | -0.644435 | 4.573468 | 0.121072 |
| H | 3.551625 | 4.884400 | -0.978795 |
| H | 1.138287 | 6.021885 | -1.273403 |

**C**

49

E: -825.627208637 ; H: -825.183922 ; G: -825.257526

| | | | |
|---|---|---|---|
| C | -2.159196 | 0.549540 | -0.169236 |
| C | -0.669100 | 0.774502 | -0.229147 |
| C | -1.614657 | 1.748912 | -0.894706 |
| H | -0.239294 | 1.129106 | 0.710140 |
| H | -1.771969 | 2.729049 | -0.448224 |
| H | -1.636779 | 1.738599 | -1.982685 |
| C | -2.740491 | -0.587841 | -0.966271 |
| H | -2.648311 | -1.535641 | -0.412532 |
| H | -3.806108 | -0.436205 | -1.177430 |
| H | -2.235757 | -0.718456 | -1.928557 |
| C | -2.860453 | 0.821320 | 1.144342 |
| H | -2.271520 | 1.556072 | 1.711620 |
| H | -2.867951 | -0.098141 | 1.751580 |
| C | -4.284113 | 1.330914 | 0.989967 |
| H | -4.723264 | 1.589703 | 1.959193 |
| H | -4.315117 | 2.229389 | 0.361412 |
| H | -4.938288 | 0.582888 | 0.528643 |
| C | 0.258690 | -0.081184 | -1.039874 |
| H | -0.035485 | -0.085434 | -2.094681 |

| | | | |
|---|---|---|---|
| C | 1.724504 | 0.037640 | -0.872099 |
| H | 1.999550 | 0.581087 | 0.040366 |
| C | 4.061091 | 0.429085 | -1.774007 |
| H | 4.654567 | 0.798448 | -2.618353 |
| H | 4.344974 | 1.017489 | -0.892188 |
| H | 4.364693 | -0.607994 | -1.578192 |
| C | 2.568136 | 0.529215 | -2.032439 |
| H | 2.323480 | 1.584396 | -2.250654 |
| H | 2.315049 | -0.015798 | -2.953250 |
| Zr | 1.084566 | -2.125669 | -0.425256 |
| C | 1.291883 | -3.530290 | 1.728745 |
| C | 2.183897 | -2.449483 | 1.895057 |
| C | -0.020233 | -3.012101 | 1.676075 |
| H | 1.570160 | -4.572732 | 1.626953 |
| C | 1.424712 | -1.262753 | 1.960194 |
| H | 3.264262 | -2.517310 | 1.951469 |
| C | 0.060064 | -1.604698 | 1.821608 |
| H | -0.928482 | -3.590832 | 1.551510 |
| H | 1.817970 | -0.264882 | 2.098192 |
| H | -0.774607 | -0.916168 | 1.842669 |
| C | 0.950840 | -2.470136 | -2.974693 |
| C | 2.283090 | -2.733520 | -2.586763 |
| C | 0.125410 | -3.467720 | -2.421395 |
| H | 0.620432 | -1.648797 | -3.596628 |
| C | 2.276341 | -3.894995 | -1.779053 |
| H | 3.154758 | -2.157793 | -2.869771 |
| C | 0.940431 | -4.351512 | -1.684530 |
| H | -0.951563 | -3.532721 | -2.526942 |
| H | 3.141243 | -4.362058 | -1.321816 |
| H | 0.601466 | -5.216303 | -1.126352 |

**D**

49

E: -825.624522773 ; H: -825.181375 ; G: -825.257027

| | | | |
|---|---|---|---|
| C | -2.05472425 | 0.59735918 | -0.19734689 |
| C | -0.55201379 | 0.71958521 | -0.20694359 |
| C | -1.40817965 | 1.78160510 | -0.86281527 |
| H | -0.13890521 | 1.01501377 | 0.75757248 |
| H | -1.51139679 | 2.75360044 | -0.38315195 |
| H | -1.39451659 | 1.81227826 | -1.95068399 |
| C | -2.70182738 | -0.46295577 | -1.04869853 |
| H | -2.70056783 | -1.43443756 | -0.52999128 |
| H | -3.74644625 | -0.21811215 | -1.27655462 |
| H | -2.18816081 | -0.59850702 | -2.00477701 |
| C | -2.77355022 | 0.87838776 | 1.10554582 |

| | | | |
|---|---|---|---|
| H | -2.14506900 | 1.53987664 | 1.71873156 |
| H | -2.87901448 | -0.05853296 | 1.67570975 |
| C | -4.14199664 | 1.51504585 | 0.92655775 |
| H | -4.58609119 | 1.78642670 | 1.89000148 |
| H | -4.07340070 | 2.42895324 | 0.32391027 |
| H | -4.84536469 | 0.84139499 | 0.42541199 |
| C | 0.32391153 | -0.17187738 | -1.03090043 |
| H | -0.04480950 | -0.21748147 | -2.05942987 |
| C | 1.80621366 | -0.10041324 | -0.93549100 |
| H | 2.28163449 | -0.16025139 | -1.92309066 |
| C | 2.56934444 | 2.30522503 | -0.91044271 |
| H | 3.05094675 | 3.10133603 | -0.32902532 |
| H | 3.14181952 | 2.17902416 | -1.83794407 |
| H | 1.56742554 | 2.65265729 | -1.19133734 |
| C | 2.48879520 | 0.99360335 | -0.13811917 |
| H | 3.50641534 | 0.68310411 | 0.14581200 |
| H | 1.96704817 | 1.18172906 | 0.80841548 |
| Zr | 1.13618465 | -2.22586714 | -0.38984667 |
| C | 1.29437200 | -3.63625906 | 1.76916652 |
| C | 2.22360498 | -2.58663193 | 1.92268149 |
| C | 0.00086977 | -3.07366556 | 1.71598425 |
| H | 1.53553079 | -4.68839777 | 1.67154517 |
| C | 1.50664409 | -1.37216643 | 1.98253340 |
| H | 3.30079518 | -2.69324312 | 1.97962573 |
| C | 0.12870100 | -1.66924339 | 1.85260490 |
| H | -0.92720485 | -3.62160887 | 1.59763843 |
| H | 1.93784712 | -0.39136422 | 2.12595091 |
| H | -0.68263386 | -0.95440189 | 1.88161387 |
| C | 1.08483171 | -2.53702245 | -2.94405355 |
| C | 2.37854851 | -2.90060300 | -2.51472953 |
| C | 0.16763007 | -3.47613618 | -2.43229059 |
| H | 0.83909115 | -1.67894809 | -3.55610164 |
| C | 2.25991348 | -4.06723708 | -1.72282845 |
| H | 3.29643663 | -2.37504769 | -2.74606897 |
| C | 0.89108339 | -4.42762556 | -1.68214587 |
| H | -0.90653652 | -3.46069308 | -2.57817425 |
| H | 3.07148354 | -4.60089987 | -1.24191141 |
| H | 0.47085173 | -5.27219818 | -1.14872597 |

**TSA-Imin**

49

E: -825.610705200 ; H: -825.170032 ; G: -825.240680

| | | | |
|---|---|---|---|
| C | -0.37276649 | -0.67546230 | 0.11218227 |
| C | 1.04224647 | -0.13939069 | 0.16058788 |
| C | -0.31200370 | 0.64404766 | -0.55290430 |

| | | | |
|---|---|---|---|
| Zr | 1.40163755 | 2.49231906 | -0.02248399 |
| C | 0.68778704 | 2.93046669 | 2.40528222 |
| C | 1.96741628 | 2.34142051 | 2.49382776 |
| C | 0.83172725 | 4.19541614 | 1.78887112 |
| H | -0.23586988 | 2.50510238 | 2.77316983 |
| C | 2.89945485 | 3.23492732 | 1.92537366 |
| H | 2.19631664 | 1.37373109 | 2.92463922 |
| C | 2.20053936 | 4.38090606 | 1.49977167 |
| H | 0.03966108 | 4.90499892 | 1.59205145 |
| H | 3.96537089 | 3.06819676 | 1.84119881 |
| H | 2.63471469 | 5.24996433 | 1.02192805 |
| C | 2.14686203 | 1.84975983 | -2.38721596 |
| C | 2.01998926 | 3.25278536 | -2.40853865 |
| C | 3.24812335 | 1.52384786 | -1.55501712 |
| H | 1.53460928 | 1.14373848 | -2.93488679 |
| C | 3.02244114 | 3.79330124 | -1.58070690 |
| H | 1.29030092 | 3.82126647 | -2.96619051 |
| C | 3.79753441 | 2.72348712 | -1.07658029 |
| H | 3.62024277 | 0.52774544 | -1.34859782 |
| H | 3.19041330 | 4.84624274 | -1.39176028 |
| H | 4.64658661 | 2.81116608 | -0.41073605 |
| H | -0.16347914 | 0.56133988 | -1.63085860 |
| C | -0.81607386 | 1.89753694 | -0.04652630 |
| H | -1.33368866 | 1.86443875 | 0.90767585 |
| C | -0.69144684 | 3.16299338 | -0.77249541 |
| C | -0.59135217 | -1.86621105 | -0.80172591 |
| H | -1.65799711 | -1.92404807 | -1.06401024 |
| H | -0.05500625 | -1.68374080 | -1.74349243 |
| C | -1.09768916 | -0.76251617 | 1.43011438 |
| H | -0.89534185 | 0.10495631 | 2.06584992 |
| H | -2.18308274 | -0.81787765 | 1.27594048 |
| H | 1.76200891 | -0.63155793 | -0.48932147 |
| H | 1.39049175 | 0.06900263 | 1.17257724 |
| C | -0.13514925 | -3.18643881 | -0.20647892 |
| H | -0.75158579 | -3.48428546 | 0.64830612 |
| H | -0.19289738 | -3.99264987 | -0.94441214 |
| H | 0.90435372 | -3.12765882 | 0.13975831 |
| H | -0.78833478 | -1.65184797 | 1.99031286 |
| C | -1.12198485 | 3.23829140 | -2.22820644 |
| H | -0.82821386 | 4.21117170 | -2.64852406 |
| H | -0.59965993 | 2.48865611 | -2.83937953 |
| C | -2.62291541 | 3.05678530 | -2.41010962 |
| H | -3.18170692 | 3.81995010 | -1.85399914 |
| H | -2.92100420 | 3.12813984 | -3.46357220 |
| H | -2.94704586 | 2.07804892 | -2.03634385 |

H -1.15869096 3.97567991 -0.20859434

**TSB-IMaj**

49

E: -825.615411741 ; H: -825.173809 ; G: -825.246764

| | | | |
|---|---|---|---|
| C | -0.404613 | -0.655344 | 0.112666 |
| C | 1.002067 | -0.101100 | 0.101030 |
| C | -0.403891 | 0.697560 | -0.484004 |
| Zr | 1.320101 | 2.541277 | 0.028418 |
| C | 0.702754 | 3.058839 | 2.462080 |
| C | 1.819226 | 2.203362 | 2.557500 |
| C | 1.121074 | 4.261719 | 1.844438 |
| H | -0.293986 | 2.844184 | 2.823172 |
| C | 2.928381 | 2.859563 | 1.988474 |
| H | 1.820114 | 1.207101 | 2.983552 |
| C | 2.501356 | 4.135208 | 1.556925 |
| H | 0.505261 | 5.130865 | 1.653734 |
| H | 3.927602 | 2.452178 | 1.892393 |
| H | 3.118414 | 4.889112 | 1.085201 |
| C | 2.083957 | 1.781773 | -2.326027 |
| C | 1.677433 | 3.112126 | -2.504608 |
| C | 3.229405 | 1.777611 | -1.484831 |
| H | 1.611392 | 0.907019 | -2.755696 |
| C | 2.553740 | 3.943643 | -1.773972 |
| H | 0.828633 | 3.445619 | -3.086617 |
| C | 3.529274 | 3.116283 | -1.173793 |
| H | 3.795187 | 0.906464 | -1.176257 |
| H | 2.500903 | 5.023520 | -1.708447 |
| H | 4.351816 | 3.451410 | -0.554643 |
| H | -0.312343 | 0.681836 | -1.572385 |
| C | -0.905845 | 1.918065 | 0.092672 |
| H | -1.379991 | 1.856882 | 1.070271 |
| C | -0.808327 | 3.219373 | -0.555782 |
| H | -0.845435 | 3.172850 | -1.653020 |
| C | -0.658893 | -1.805543 | -0.842308 |
| H | -1.737652 | -1.875489 | -1.045467 |
| H | -0.181502 | -1.567625 | -1.803522 |
| C | -1.039852 | -0.814483 | 1.470538 |
| H | -0.820798 | 0.035494 | 2.125078 |
| H | -2.131022 | -0.897281 | 1.386661 |
| H | 1.684763 | -0.537849 | -0.624848 |
| H | 1.416616 | 0.041768 | 1.098426 |
| C | -0.141066 | -3.140686 | -0.336518 |
| H | -0.701724 | -3.491575 | 0.536173 |
| H | -0.222200 | -3.914348 | -1.106469 |

| | | | |
|---|---|---|---|
| H | 0.914947 | -3.073210 | -0.045799 |
| H | -0.669084 | -1.713244 | 1.975746 |
| C | -1.697912 | 4.317963 | -0.028585 |
| H | -1.320520 | 5.297772 | -0.356212 |
| H | -1.657881 | 4.334874 | 1.070502 |
| C | -3.148462 | 4.169042 | -0.470863 |
| H | -3.565889 | 3.214794 | -0.127756 |
| H | -3.785209 | 4.973227 | -0.082943 |
| H | -3.225726 | 4.182969 | -1.565136 |

**TSI-C**

49

E: -825.559885794 ; H: -825.118789 ; G: -825.190989

| | | | |
|---|---|---|---|
| C | -0.291481 | -0.555587 | 0.106670 |
| C | 1.093368 | -0.003000 | 0.350215 |
| C | -0.280874 | 0.839880 | -0.410960 |
| Zr | 1.525887 | 2.570473 | 0.048836 |
| C | 0.924818 | 3.987005 | 2.100480 |
| C | 1.078190 | 2.631486 | 2.481941 |
| C | 2.172571 | 4.463228 | 1.667325 |
| H | -0.000162 | 4.550716 | 2.114994 |
| C | 2.445616 | 2.267534 | 2.298294 |
| H | 0.293143 | 1.994525 | 2.870676 |
| C | 3.110480 | 3.411181 | 1.791122 |
| H | 2.373190 | 5.452278 | 1.272584 |
| H | 2.904386 | 1.322906 | 2.562884 |
| H | 4.161957 | 3.468449 | 1.538385 |
| C | 2.311055 | 1.618196 | -2.252850 |
| C | 1.552322 | 2.762304 | -2.541004 |
| C | 3.440620 | 1.996492 | -1.495507 |
| H | 2.056719 | 0.604819 | -2.540100 |
| C | 2.196284 | 3.885429 | -1.963284 |
| H | 0.608771 | 2.781368 | -3.075301 |
| C | 3.370105 | 3.408513 | -1.321093 |
| H | 4.230715 | 1.341120 | -1.150891 |
| H | 1.889999 | 4.919173 | -2.056583 |
| H | 4.110177 | 4.024811 | -0.827122 |
| H | -0.077374 | 0.868726 | -1.481719 |
| C | -0.963589 | 2.015851 | 0.084360 |
| H | -0.591432 | 3.022244 | -0.296909 |
| C | -2.090984 | 2.139272 | 0.820425 |
| H | -2.695493 | 1.255314 | 1.024215 |
| C | -0.393187 | -1.622378 | -0.970116 |
| H | -1.432328 | -1.673882 | -1.328339 |
| H | 0.210184 | -1.303938 | -1.832411 |

| | | | |
|---|---|---|---|
| C | -1.066841 | -0.884998 | 1.360146 |
| H | -1.052564 | -0.061751 | 2.079815 |
| H | -2.114962 | -1.124030 | 1.138975 |
| H | 1.883601 | -0.433595 | -0.264659 |
| H | 1.331400 | 0.083601 | 1.409106 |
| C | -2.581535 | 3.430308 | 1.381172 |
| H | -2.296064 | 3.519041 | 2.443945 |
| H | -2.069906 | 4.263125 | 0.873448 |
| C | -4.089847 | 3.587727 | 1.266873 |
| H | -4.608692 | 2.772957 | 1.785788 |
| H | -4.432517 | 4.530256 | 1.706133 |
| H | -4.410148 | 3.566937 | 0.219192 |
| C | 0.066709 | -2.996541 | -0.517997 |
| H | -0.608154 | -3.430287 | 0.227419 |
| H | 0.109577 | -3.694883 | -1.359795 |
| H | 1.068683 | -2.952771 | -0.072973 |
| H | -0.622941 | -1.756369 | 1.856718 |

**Imin**

49

E: -825.634660050 ; H: -825.190660 ; G: -825.262252

| | | | |
|---|---|---|---|
| C | -0.96568223 | -2.48204056 | 0.04193353 |
| C | 0.51738430 | -2.10814342 | 0.20496363 |
| C | -1.40681553 | -1.24449592 | -0.71338096 |
| Zr | 0.53898354 | 0.32750212 | -0.10353019 |
| C | -0.32428339 | 1.12070189 | 2.21092596 |
| C | 0.56710506 | 0.06247084 | 2.45447959 |
| C | 0.40629655 | 2.19719849 | 1.65518423 |
| H | -1.38540481 | 1.12324187 | 2.42388331 |
| C | 1.85299252 | 0.46923210 | 2.03795082 |
| H | 0.31369493 | -0.90298747 | 2.87084054 |
| C | 1.75365093 | 1.79981244 | 1.57072815 |
| H | 0.00227954 | 3.15590082 | 1.35519271 |
| H | 2.75474141 | -0.12992072 | 2.08043258 |
| H | 2.56837594 | 2.40619352 | 1.19709050 |
| C | 1.31235649 | -0.23064196 | -2.48746437 |
| C | 1.11403097 | 1.16599393 | -2.46886303 |
| C | 2.40589708 | -0.52574923 | -1.64131428 |
| H | 0.74286868 | -0.95117110 | -3.06179653 |
| C | 2.06233818 | 1.73030737 | -1.59497567 |
| H | 0.36725241 | 1.71365936 | -3.02421538 |
| C | 2.86974299 | 0.68170177 | -1.09529497 |
| H | 2.81944422 | -1.50596752 | -1.44929694 |
| H | 2.15728577 | 2.78244488 | -1.35585739 |
| H | 3.69584975 | 0.78497945 | -0.40298165 |

| | | | |
|---|---|---|---|
| H | -1.14662091 | -1.26413806 | -1.77571256 |
| C | -1.89307153 | -0.07935489 | -0.18402442 |
| H | -2.20325552 | -0.11813577 | 0.85944507 |
| C | -1.67797794 | 1.22726339 | -0.74930243 |
| C | -1.19028441 | -3.71870826 | -0.83666107 |
| H | -2.26654373 | -3.80433021 | -1.05888819 |
| H | -0.69440029 | -3.53406290 | -1.80247910 |
| C | -1.67980620 | -2.64891438 | 1.37325481 |
| H | -1.59252127 | -1.76170365 | 2.00822014 |
| H | -2.74976005 | -2.85143050 | 1.22861529 |
| H | 1.10286473 | -2.58130704 | -0.59192158 |
| H | 0.94149106 | -2.42685386 | 1.16689031 |
| C | -0.67804909 | -5.02357879 | -0.25672327 |
| H | -1.25036124 | -5.33467908 | 0.62401483 |
| H | -0.74584657 | -5.83465437 | -0.98933580 |
| H | 0.37334208 | -4.93846699 | 0.04352986 |
| H | -1.24889401 | -3.48250966 | 1.93863286 |
| C | -2.03128719 | 1.46728869 | -2.20378370 |
| C | -3.53914136 | 1.50616815 | -2.42208799 |
| H | -1.60148102 | 0.69222897 | -2.85241348 |
| H | -1.59781456 | 2.41777717 | -2.54357174 |
| H | -3.79532937 | 1.71205562 | -3.46820925 |
| H | -3.99877149 | 0.54954927 | -2.14642313 |
| H | -4.00508453 | 2.28426515 | -1.80539568 |
| H | -2.06620858 | 2.02880280 | -0.11237270 |

**IMaj**

49

E: -825.640176263 ; H: -825.196907 ; G: -825.268908

| | | | |
|---|---|---|---|
| C | -0.984294 | -2.477799 | 0.092464 |
| C | 0.504885 | -2.098119 | 0.143205 |
| C | -1.478187 | -1.229366 | -0.610322 |
| Zr | 0.435587 | 0.336613 | -0.073640 |
| C | -0.271561 | 0.871331 | 2.343545 |
| C | 0.723343 | -0.116925 | 2.436590 |
| C | 0.319394 | 2.042039 | 1.809216 |
| H | -1.303151 | 0.766443 | 2.652027 |
| C | 1.929558 | 0.424388 | 1.944181 |
| H | 0.589148 | -1.122418 | 2.809683 |
| C | 1.680288 | 1.770570 | 1.583179 |
| H | -0.184396 | 2.981635 | 1.620761 |
| H | 2.875761 | -0.097694 | 1.862874 |
| H | 2.402821 | 2.467888 | 1.179195 |
| C | 1.148714 | -0.203121 | -2.508710 |
| C | 0.774883 | 1.155351 | -2.525982 |

| | | | |
|---|---|---|---|
| C | 2.301140 | -0.326617 | -1.697468 |
| H | 0.649530 | -1.012195 | -3.027632 |
| C | 1.671080 | 1.868876 | -1.704146 |
| H | -0.066454 | 1.578269 | -3.058996 |
| C | 2.629710 | 0.951304 | -1.213751 |
| H | 2.837709 | -1.242071 | -1.485736 |
| H | 1.641175 | 2.932922 | -1.501583 |
| H | 3.467846 | 1.186308 | -0.570701 |
| H | -1.234806 | -1.195813 | -1.677016 |
| C | -2.017222 | -0.097413 | -0.049695 |
| H | -2.379700 | -0.161337 | 0.978364 |
| C | -1.798420 | 1.213931 | -0.569032 |
| H | -1.735773 | 1.264259 | -1.660598 |
| C | -1.281969 | -3.709620 | -0.770637 |
| H | -2.372877 | -3.788786 | -0.908072 |
| H | -0.861366 | -3.524455 | -1.771193 |
| C | -1.595967 | -2.647260 | 1.473877 |
| H | -1.469760 | -1.754848 | 2.093717 |
| H | -2.671913 | -2.859398 | 1.409929 |
| H | 1.011733 | -2.507702 | -0.739661 |
| H | 1.029456 | -2.468968 | 1.033216 |
| C | -0.734139 | -5.018875 | -0.233899 |
| H | -1.240200 | -5.327285 | 0.687411 |
| H | -0.862622 | -5.829268 | -0.959162 |
| H | 0.337502 | -4.940664 | -0.013950 |
| H | -1.118745 | -3.474507 | 2.011010 |
| C | -2.528404 | 2.381777 | 0.041281 |
| H | -2.033532 | 3.321082 | -0.244262 |
| H | -2.461419 | 2.329569 | 1.137654 |
| C | -3.996194 | 2.442804 | -0.360777 |
| H | -4.523738 | 1.536267 | -0.039291 |
| H | -4.509482 | 3.304175 | 0.082641 |
| H | -4.104584 | 2.513297 | -1.449685 |

## References


[1]S. Harada, H. Kiyono, R. Nishio, T. Taguchi, Y. Hanzawa, *J. Org. Chem.* **1997**, *62*, 3994.
[2]S. R. Nagarajan, H. –F. Lu, A. F. Gasiecki, I. K. Khanna, M. D. Parikh, B. N. Desai, T. E. Rogers, M. Clare, B. B. Chen, M. A. Russell, J. L. Keene, T. Duffin, V. W. Engleman, M. B. Finn, S. K. Freeman, J. A. Klover, G. A. Nickols, M. A. Nickols, K. E. Shannon, C. A. Steininger, W. F. Westlin, M. M. Westlin, M. L. Williams, *Bioorg. Med. Chem.* **2007**, *15*, 3390.
[3]C. Mandès, S. Renard, M. Rofoo, M. –C. Roux, G. Rousseau, *Eur. J. Org. Chem.* **2003**, 463.
[4]Y. Zhao and D. G. Truhlar, *Theor. Chem. Acc.*, **2008**, *120*, 215**.**
[5]Gaussian 09, Revision D.01, M. J. Frisch, G. W. Trucks, H. B. Schlegel, G. E. Scuseria, M. A. Robb, J. R. Cheeseman, G. Scalmani, V. Barone, B. Mennucci, G. A. Petersson, H. Nakatsuji, M. Caricato, X. Li, H. P. Hratchian, A. F. Izmaylov, J. Bloino, G. Zheng, J. L. Sonnenberg, M. Hada, M. Ehara, K. Toyota, R. Fukuda, J. Hasegawa, M. Ishida, T. Nakajima, Y. Honda, O. Kitao, H. Nakai, T. Vreven, J. A. Montgomery, Jr., J. E. Peralta, F. Ogliaro, M. Bearpark, J. J. Heyd, E. Brothers, K. N. Kudin, V. N. Staroverov, R. Kobayashi, J. Normand, K. Raghavachari, A. Rendell, J. C. Burant, S. S. Iyengar, J. Tomasi, M. Cossi, N. Rega, J. M. Millam, M. Klene, J. E. Knox, J. B. Cross, V. Bakken, C. Adamo, J. Jaramillo, R. Gomperts, R. E. Stratmann, O. Yazyev, A. J. Austin, R. Cammi, C. Pomelli, J. W. Ochterski, R. L. Martin, K. Morokuma, V. G. Zakrzewski, G. A. Voth, P. Salvador, J. J. Dannenberg, S. Dapprich, A. D. Daniels, Ö. Farkas, J. B. Foresman, J. V. Ortiz, J. Cioslowski, and D. J. Fox, Gaussian, Inc., Wallingford CT, 2009.
[6]Y. Sun, H. Chen, *J. Chem. Theory Comput.* **2013**, *9*, 4735.
[7]D. Andrae, U. Haeussermann, M. Dolg, H. Stoll, H. Preuss, *Theor. Chim. Acta*, **1990**, *77*, 123.
[8]J.M.L. Martin, A. Sundermann*, J. Chem. Phys.*, **2001**, *114*, 3408.
[9](a) A. D. McLean, G. S. Chandler, *J. Chem. Phys.* **1980**, *72*, 5639; (b) K. Raghavachari, J. S. Binkley, R. Seeger, and J. A. Pople *J. Chem. Phys.* **1980**, *72*, 650**.**
[10]A. V. Marenich, C. J. Cramer, and D. G. Truhlar, *J. Phys. Chem. B*, **2009**, *113*, 6378**.**
[11](a) C. Gonzalez and H. B. Schlegel, *J. Chem. Phys.* **1989**, *90*, 2154; (b) C. Gonzalez and H. B. Schlegel, *J. Phys. Chem.* **1990**, *94*, 5523.

# NMR Spectra

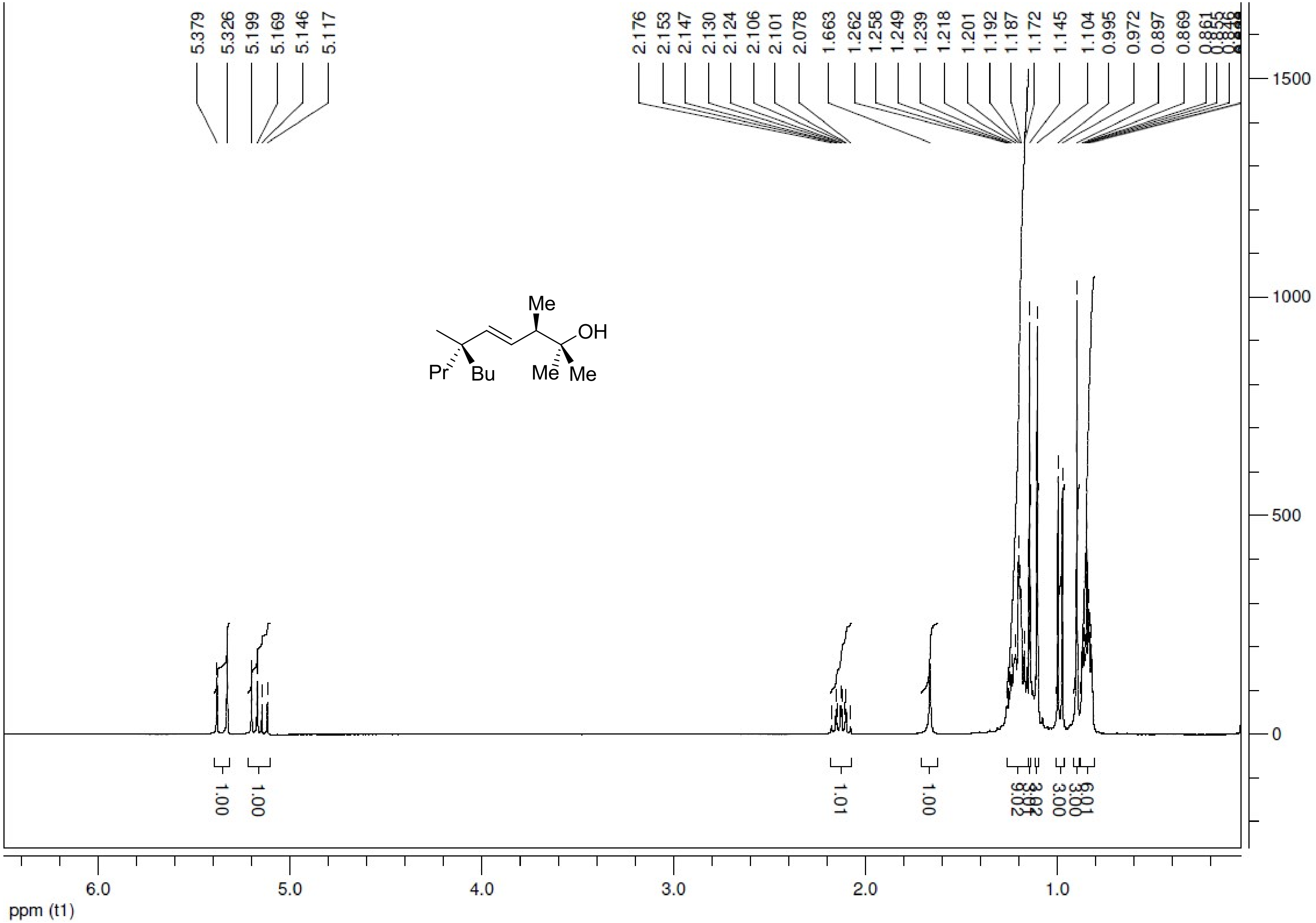

5.379
5.326
5.199
5.169
5.146
5.117
2.176
2.153
2.147
2.130
2.124
2.106
2.101
2.078
1.663
1.262
1.258
1.249
1.239
1.218
1.201
1.192
1.187
1.172
1.145
1.104
0.995
0.972
0.897
0.869
1500
1000
500
0
Me
OH
Pr
Bu
Me
Me
1.00
1.00
1.01
1.00
3.00
6.01
6.0
5.0
4.0
3.0
2.0
1.0
ppm (t1)

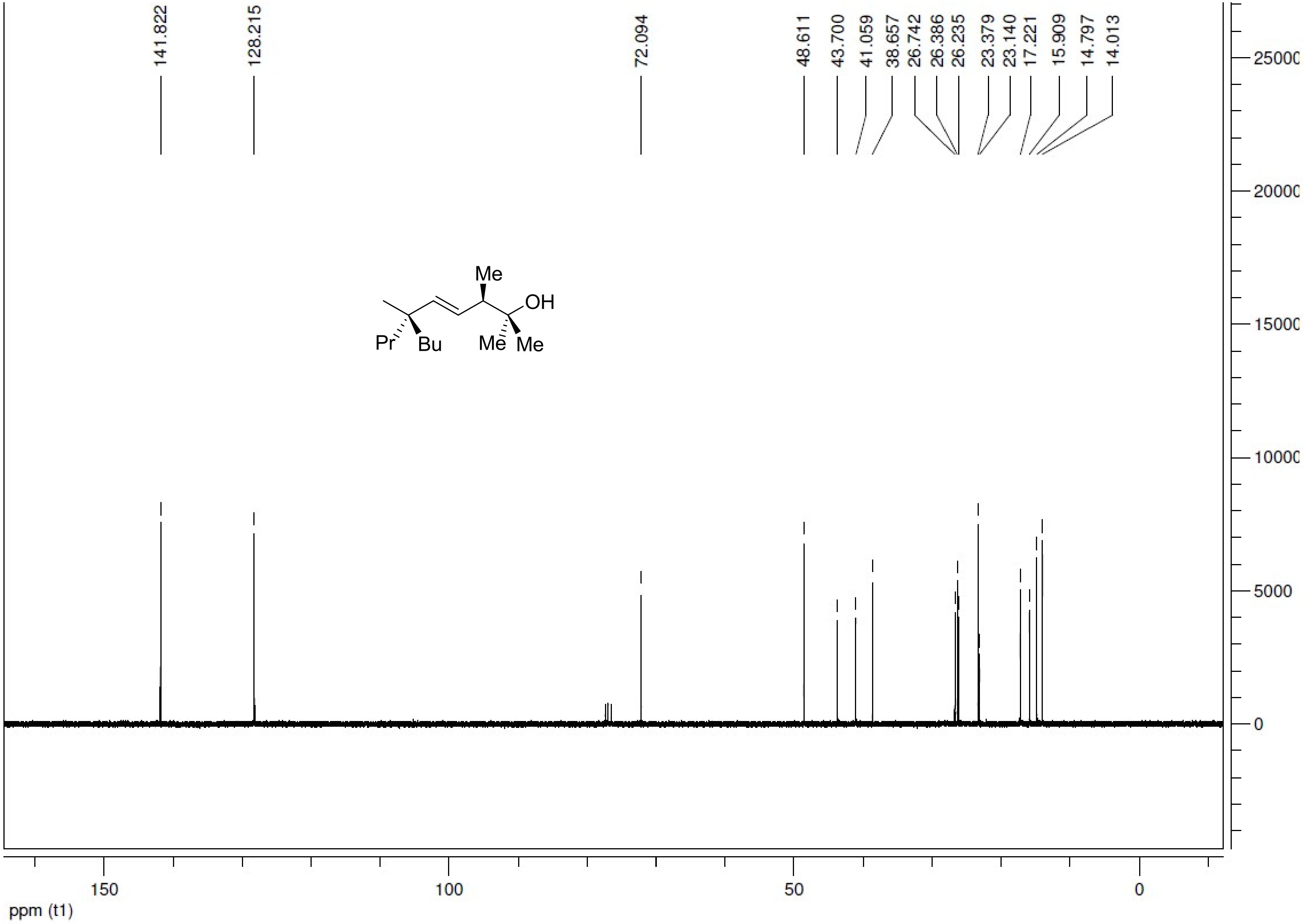
141.822
128.215
72.094
48.611
43.700
41.059
38.657
26.742
26.386
26.235
23.379
23.140
17.221
15.909
14.797
14.013
Me
OH
Pr
Bu
Me
Me
25000
20000
15000
10000
5000
0
150
100
50
0
ppm (t1)

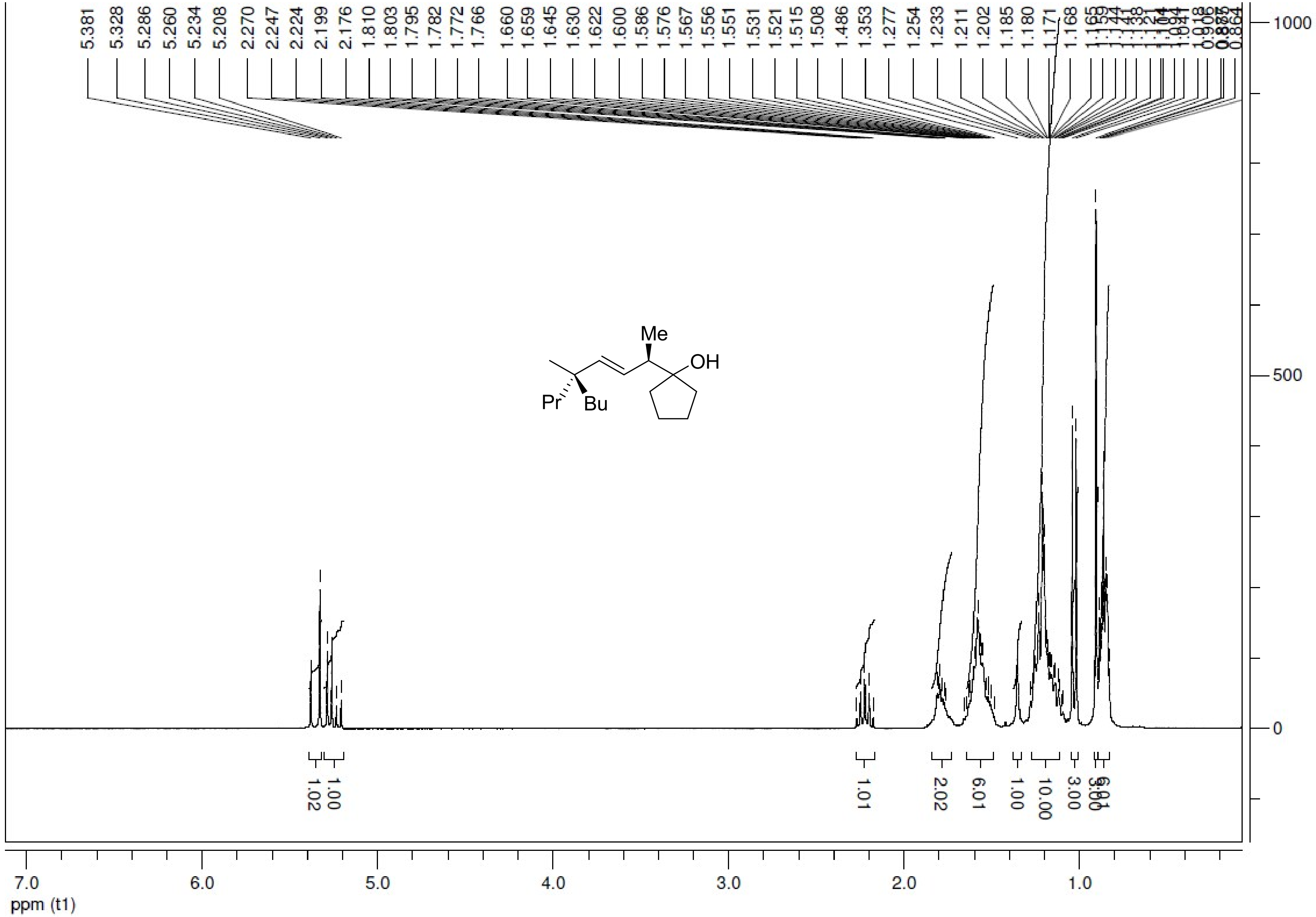

Me
OH
Pr
Bu
ppm (t1)

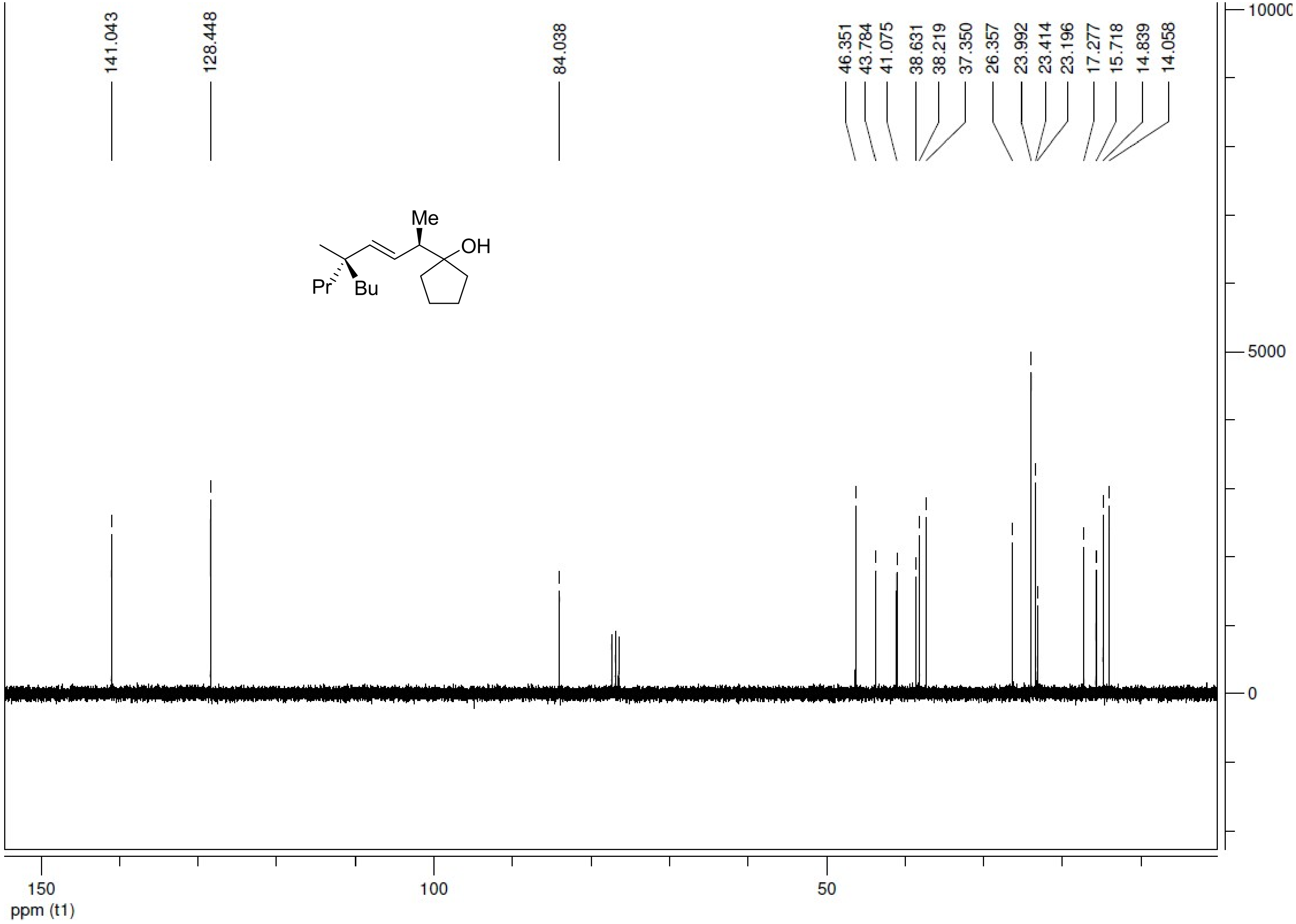
141.043
128.448
84.038
46.351
43.784
41.075
38.631
38.219
37.350
26.357
23.992
23.414
23.196
17.277
15.718
14.839
14.058
Me
OH
Pr
Bu
10000
5000
0
150
100
50
ppm (t1)

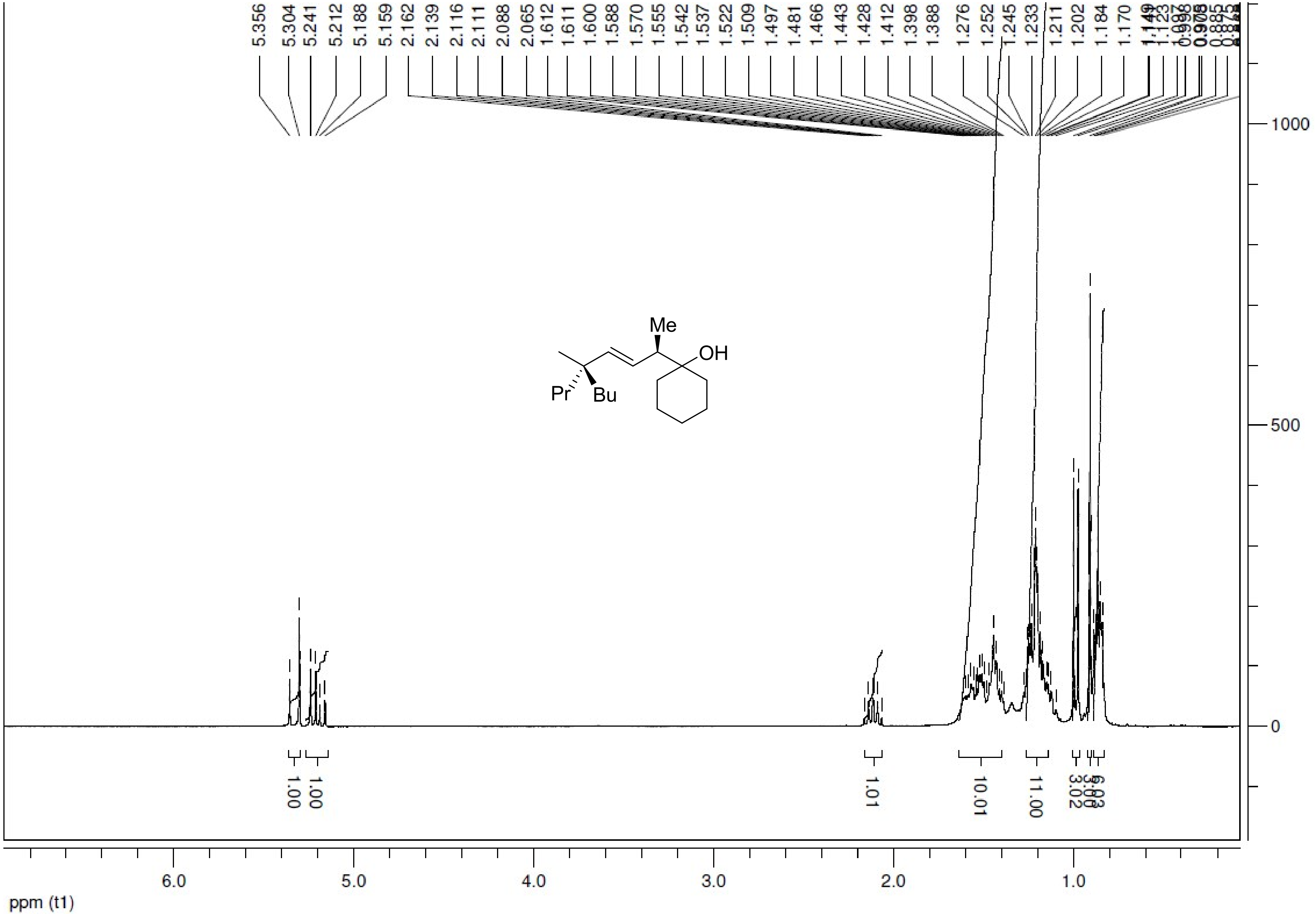
Me
OH
Pr
Bu
ppm (t1)
6.0
5.0
4.0
3.0
2.0
1.0
1000
500
0
1.00
1.00
1.01
10.01
11.00
3.02

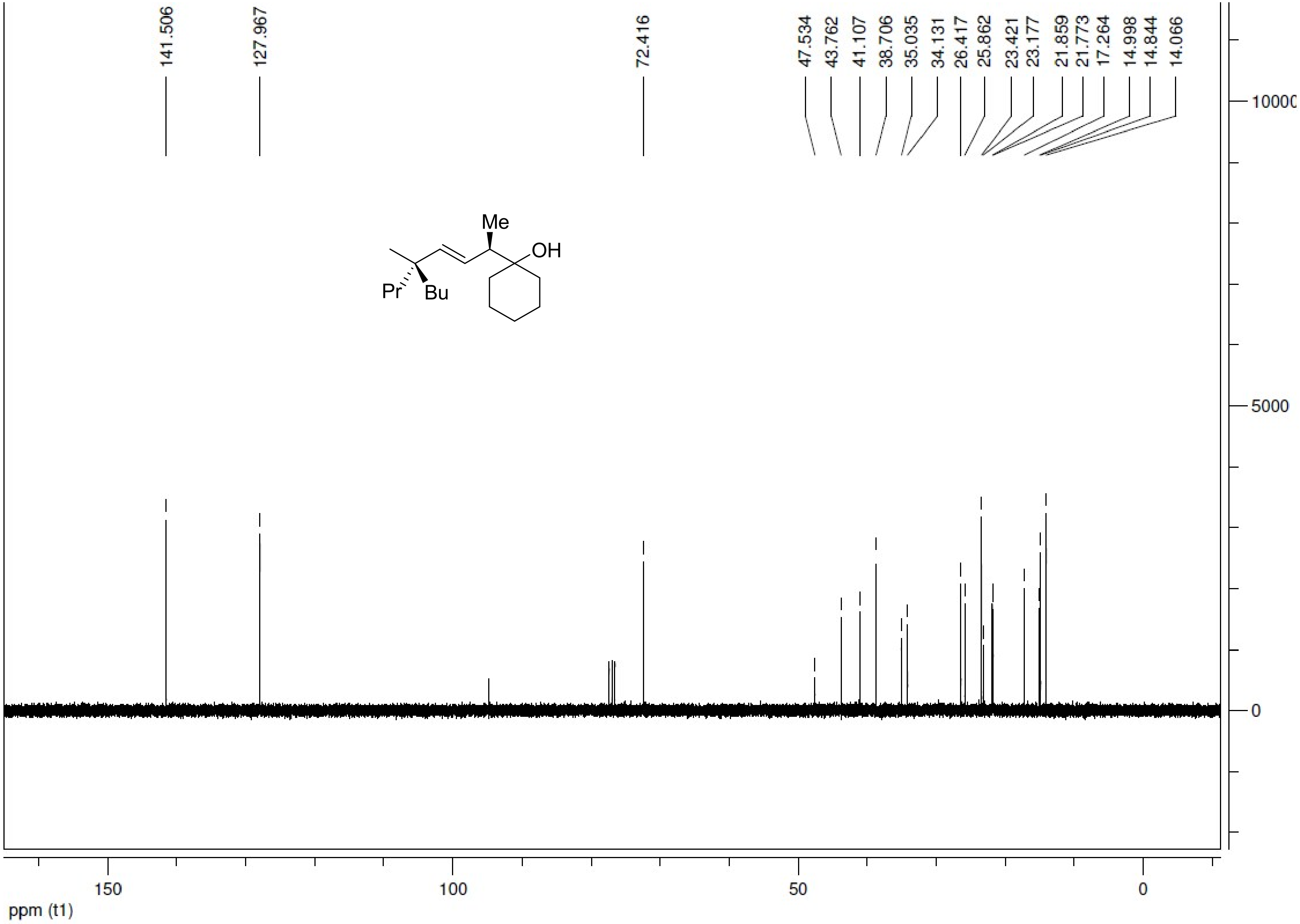
141.506
127.967
72.416
47.534
43.762
41.107
38.706
35.035
34.131
26.417
25.862
23.421
23.177
21.859
21.773
17.264
14.998
14.844
14.066
Me
OH
Pr
Bu
10000
5000
0
150
100
50
0
ppm (t1)

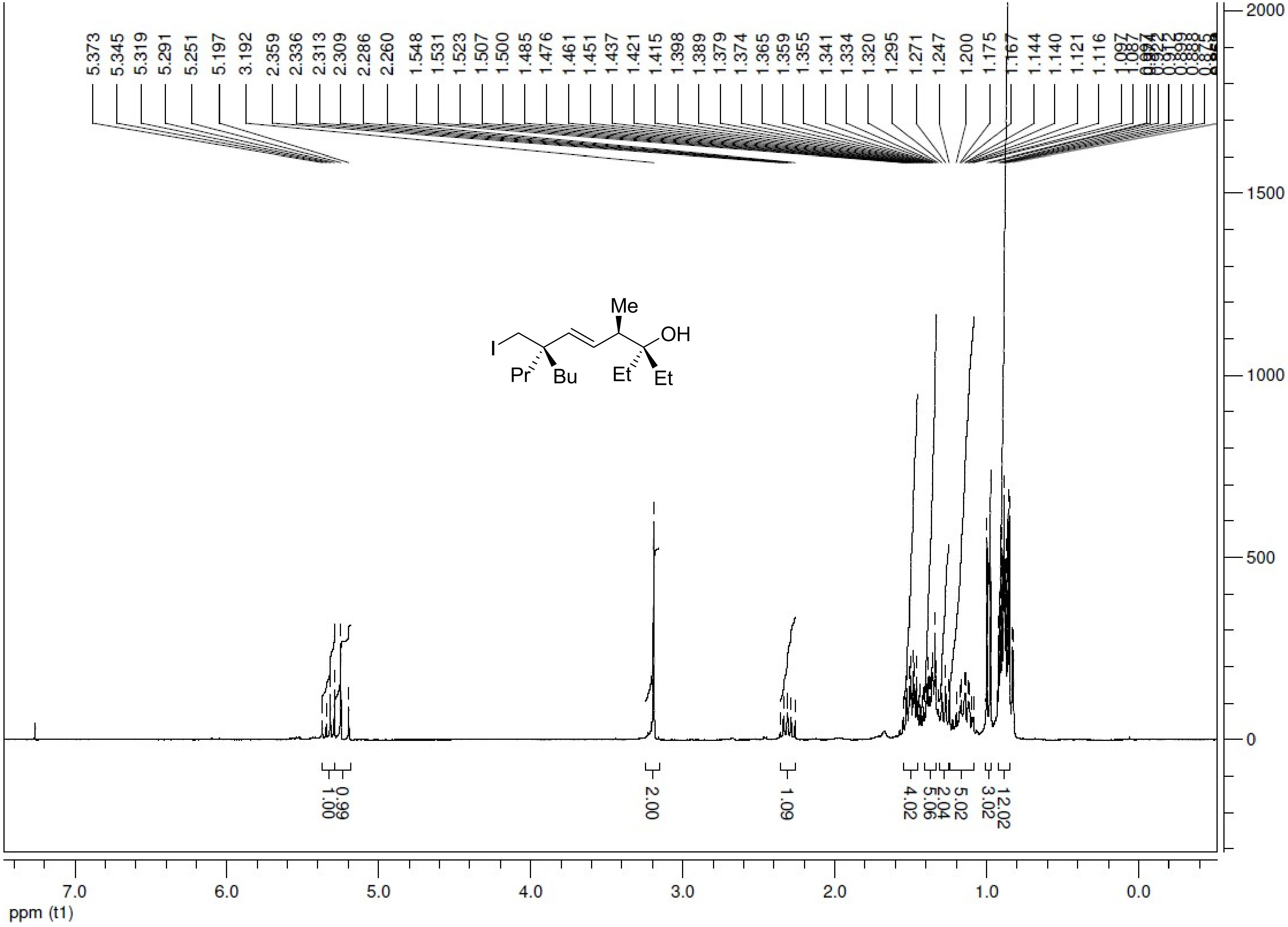

5.373
5.345
5.319
5.291
5.251
5.197
3.192
2.359
2.336
2.313
2.309
2.286
2.260
1.548
1.531
1.523
1.507
1.500
1.485
1.476
1.461
1.451
1.437
1.421
1.415
1.398
1.389
1.379
1.374
1.365
1.359
1.355
1.341
1.334
1.320
1.295
1.271
1.247
1.200
1.175
1.167
1.144
1.140
1.121
1.116
1.097
1.087
Me
OH
I
Pr
Bu
Et
Et
1.00
0.99
2.00
1.09
4.02
5.06
2.04
5.02
3.02
12.02
2000
1500
1000
500
0
7.0
6.0
5.0
4.0
3.0
2.0
1.0
0.0
ppm (t1)

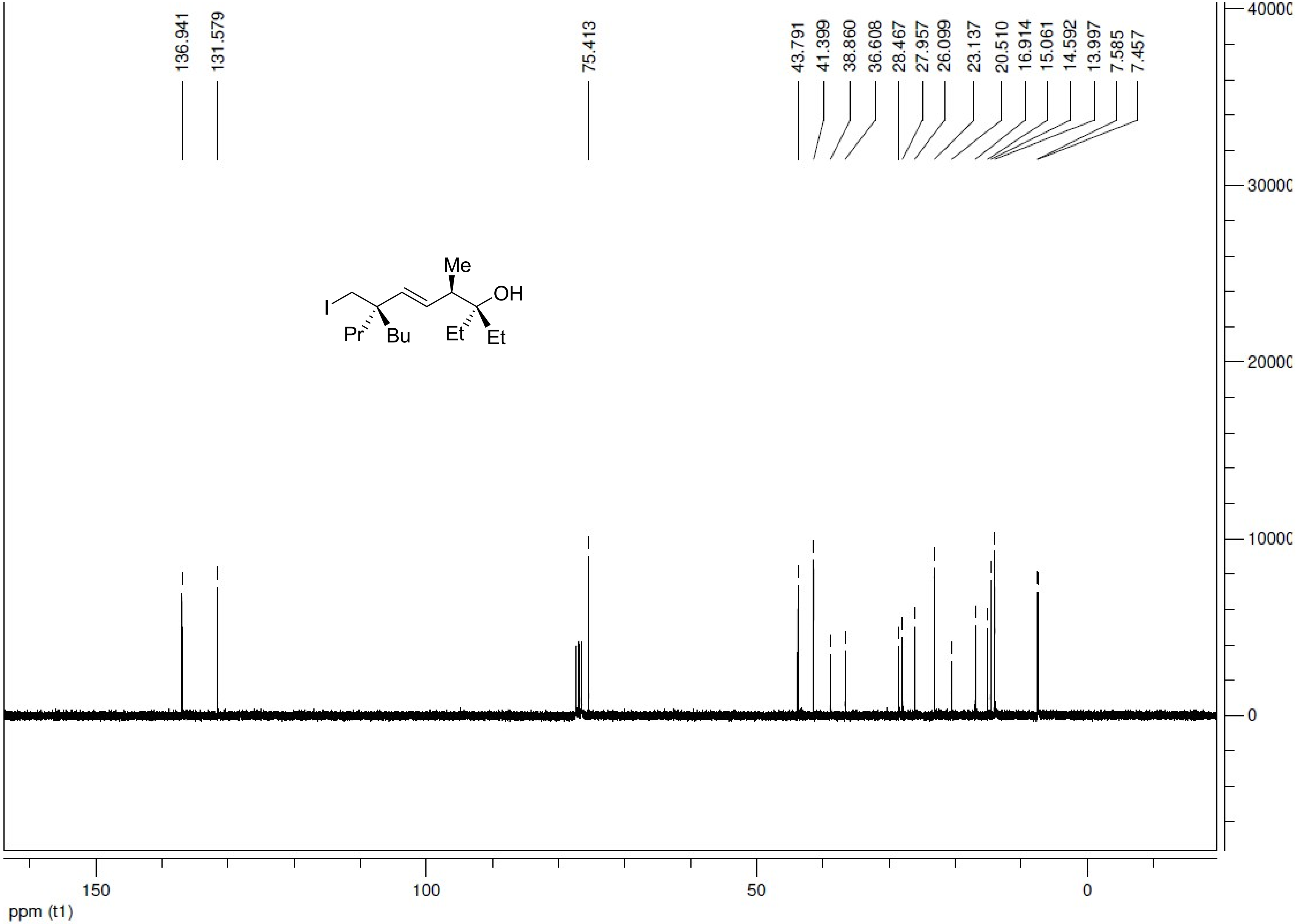
136.941
131.579
75.413
43.791
41.399
38.860
36.608
28.467
27.957
26.099
23.137
20.510
16.914
15.061
14.592
13.997
7.585
7.457
Me
OH
I
Pr
Bu
Et
Et
40000
30000
20000
10000
0
150
100
50
0
ppm (t1)

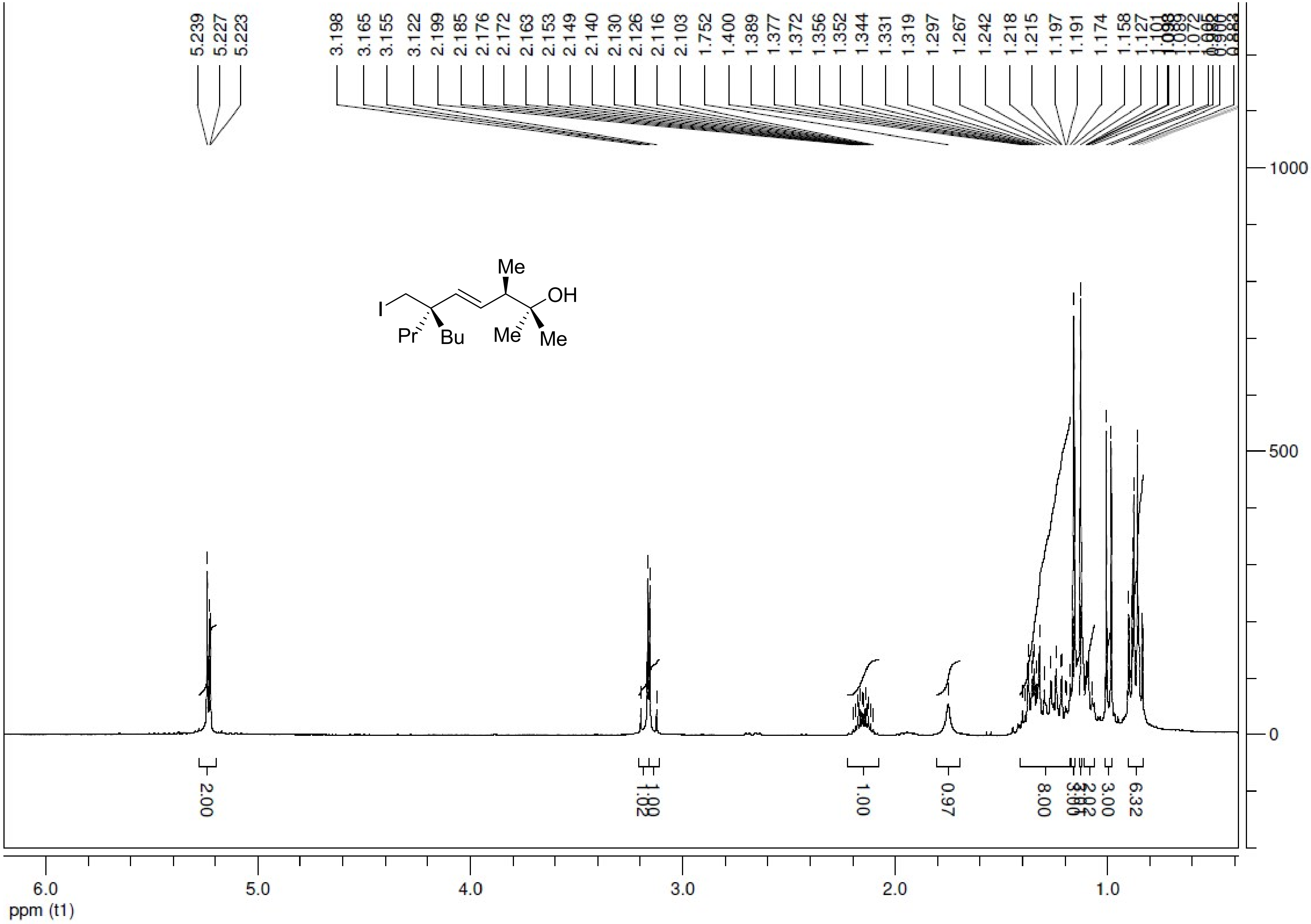
Me
OH
I
Pr
Bu
Me
Me
ppm (t1)

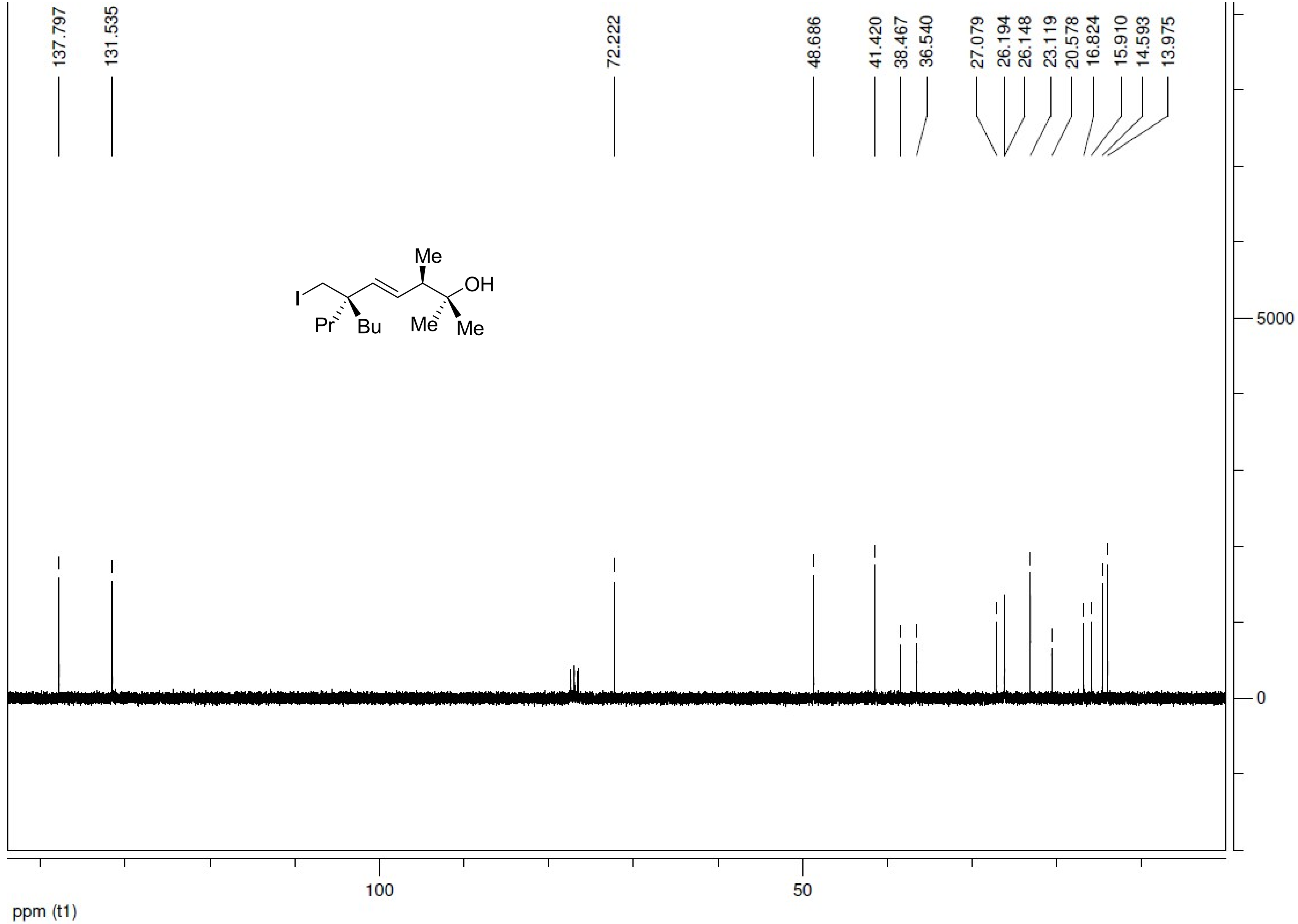
137.797
131.535
72.222
48.686
41.420
38.467
36.540
27.079
26.194
26.148
23.119
20.578
16.824
15.910
14.593
13.975
Me
OH
I
Pr
Bu
Me
Me
5000
0
100
50
ppm (t1)

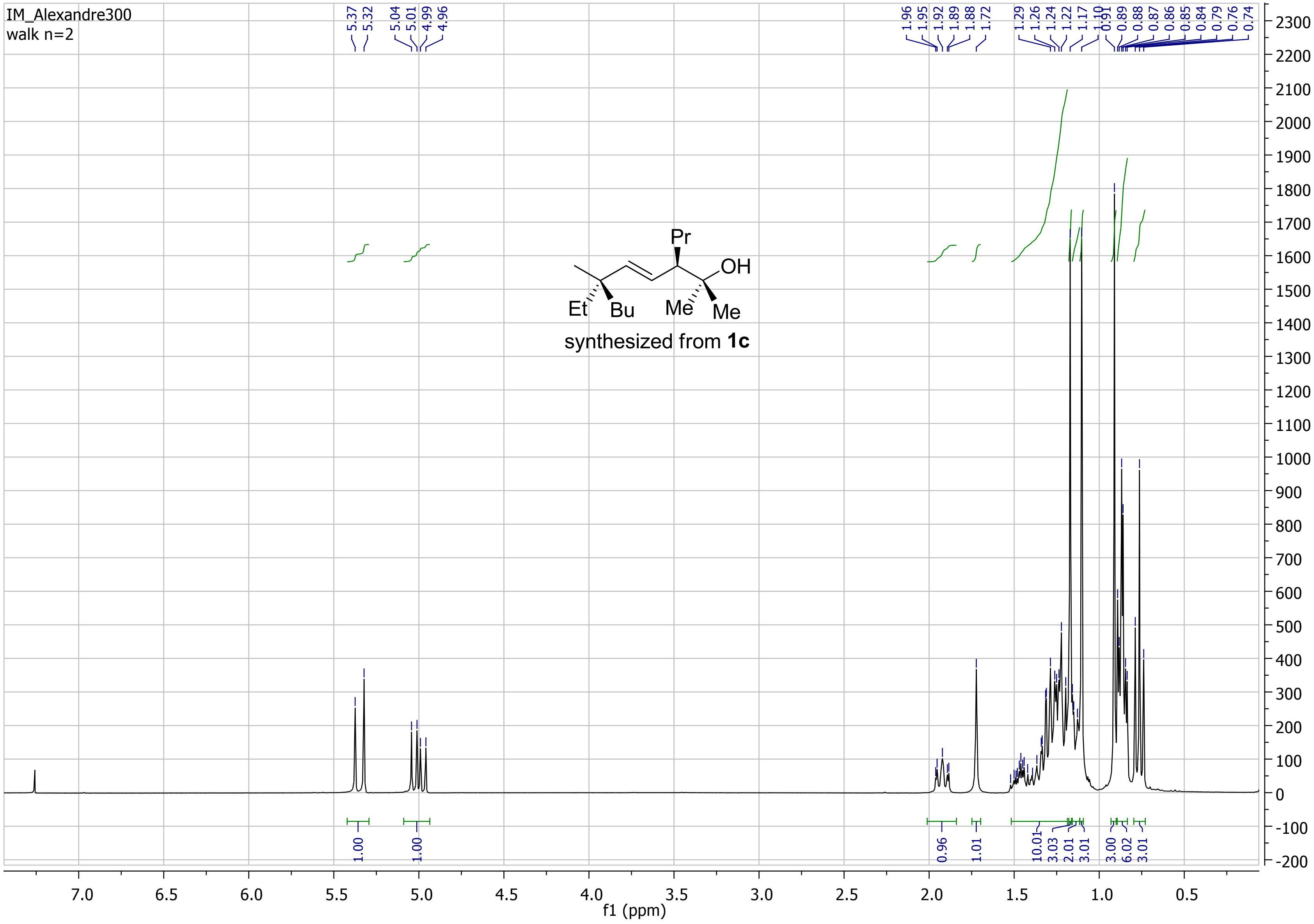
IM_Alexandre300
walk n=2
5.37
5.32
5.04
5.01
4.99
4.96
1.96
1.95
1.92
1.89
1.88
1.72
1.29
1.26
1.24
1.22
1.17
1.10
0.91
0.89
0.88
0.87
0.86
0.85
0.84
0.79
0.76
0.74
Pr
OH
Et
Bu
Me
Me
synthesized from 1c
1.00
1.00
0.96
1.01
10.01
3.03
2.01
3.01
3.00
6.02
3.01
f1 (ppm)

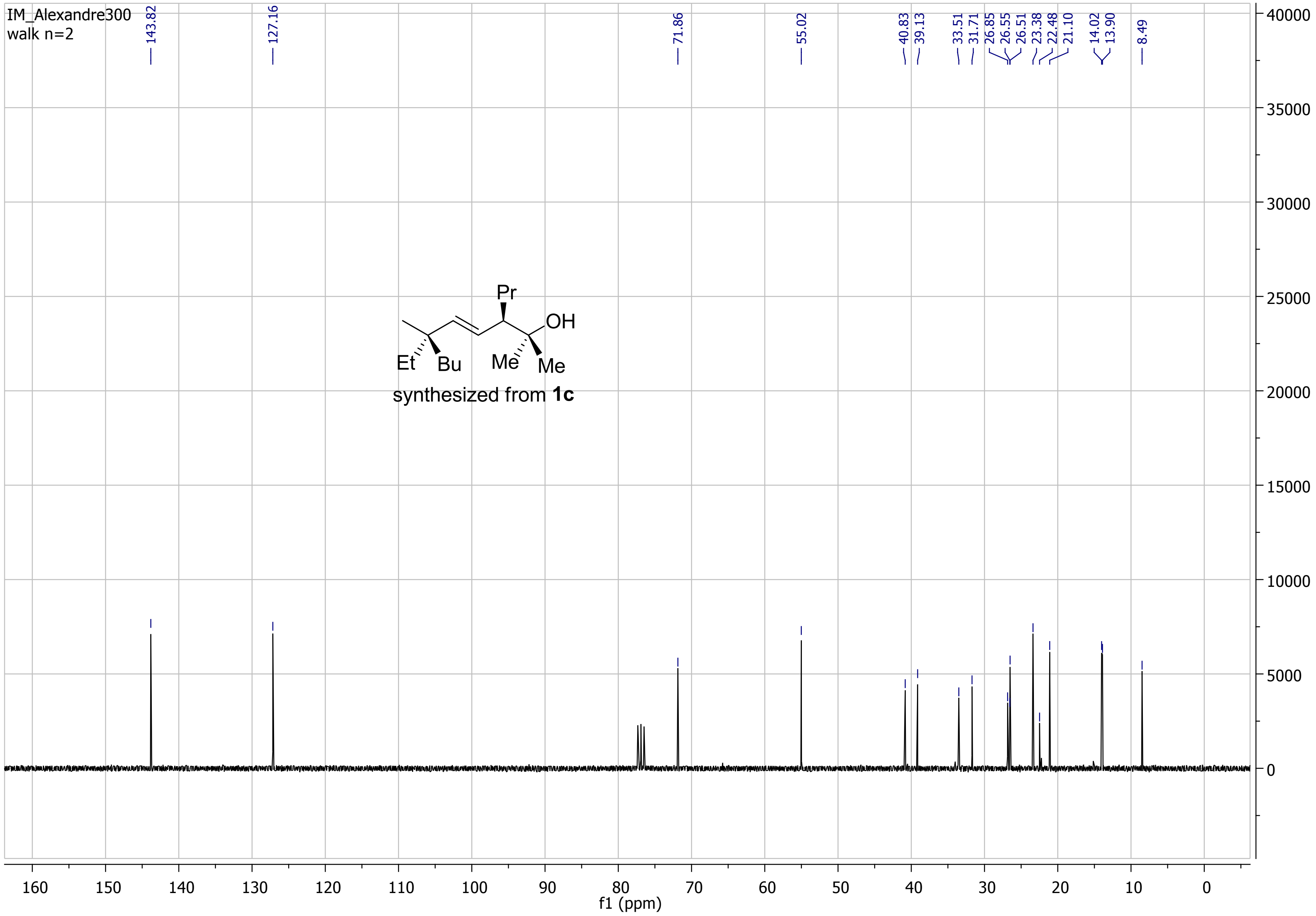
IM_Alexandre300
walk n=2
143.82
127.16
71.86
55.02
40.83
39.13
33.51
31.71
26.85
26.55
26.51
23.38
22.48
21.10
14.02
13.90
8.49
Pr
OH
Et
Bu
Me
Me
synthesized from **1c**
f1 (ppm)

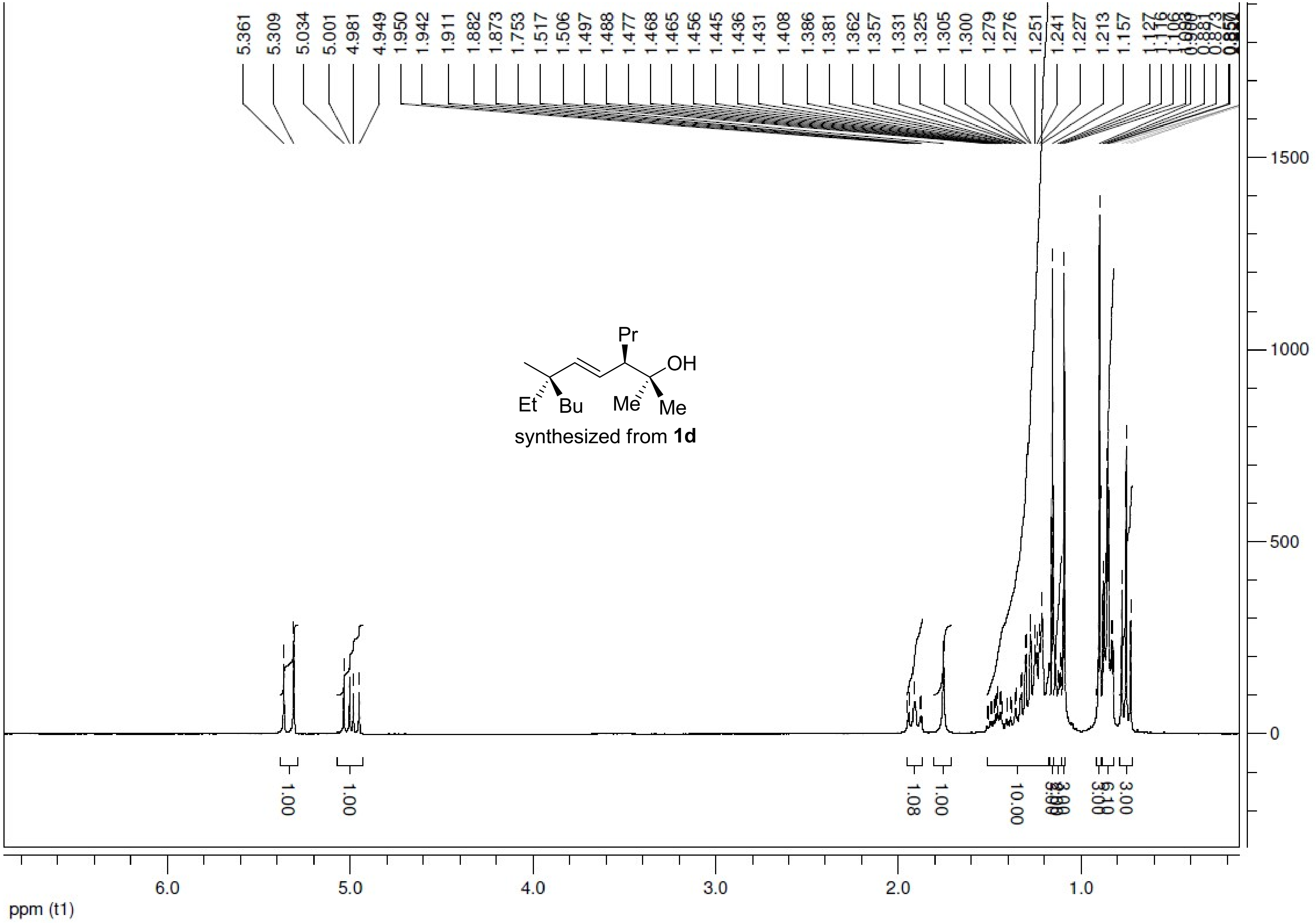
Pr
OH
Et
Bu
Me
Me
synthesized from **1d**
ppm (t1)
6.0
5.0
4.0
3.0
2.0
1.0
1500
1000
500
0

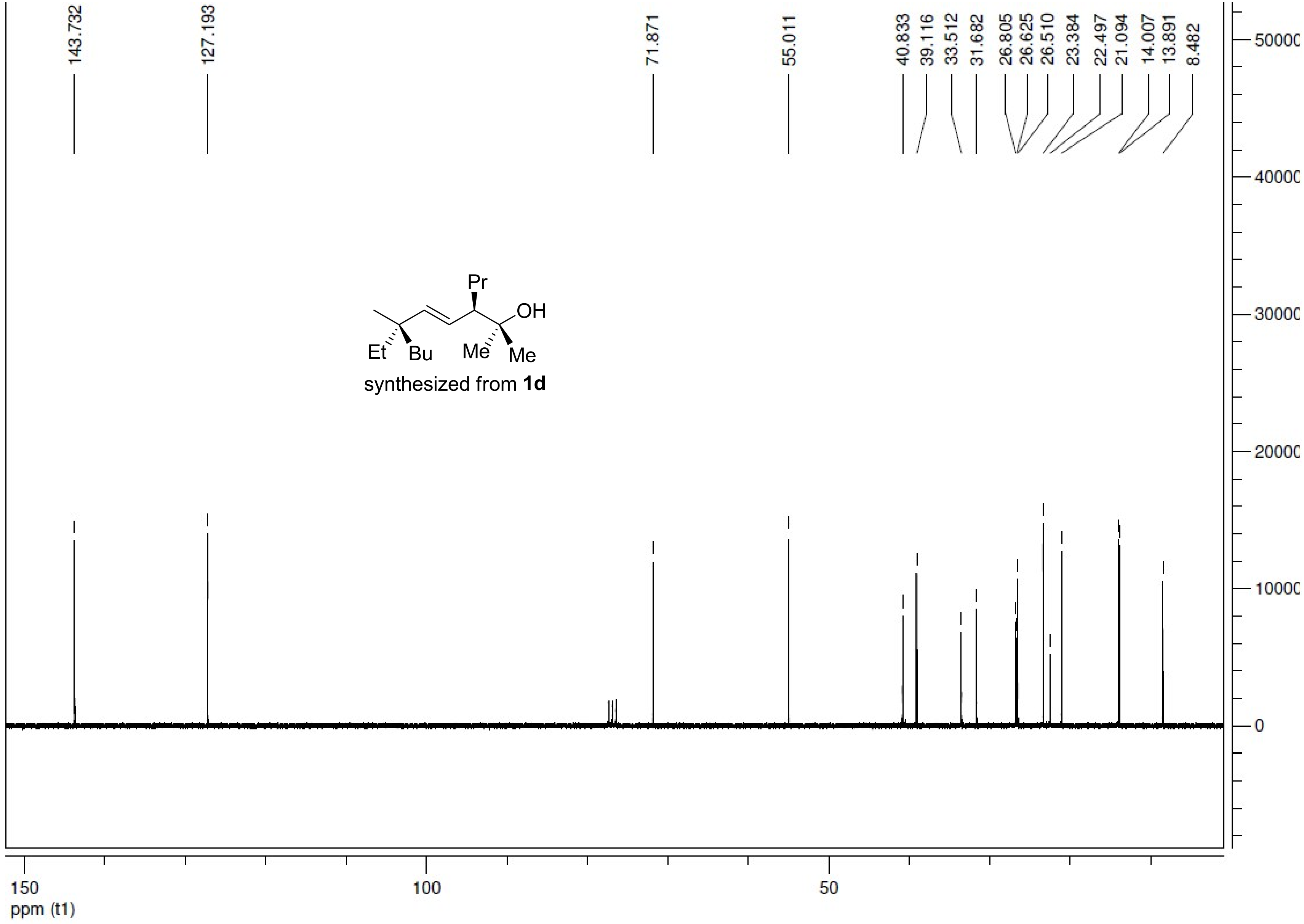
143.732
127.193
71.871
55.011
40.833
39.116
33.512
31.682
26.805
26.625
26.510
23.384
22.497
21.094
14.007
13.891
8.482
Pr
OH
Et
Bu
Me
Me
synthesized from **1d**
50000
40000
30000
20000
10000
0
150
100
50
ppm (t1)

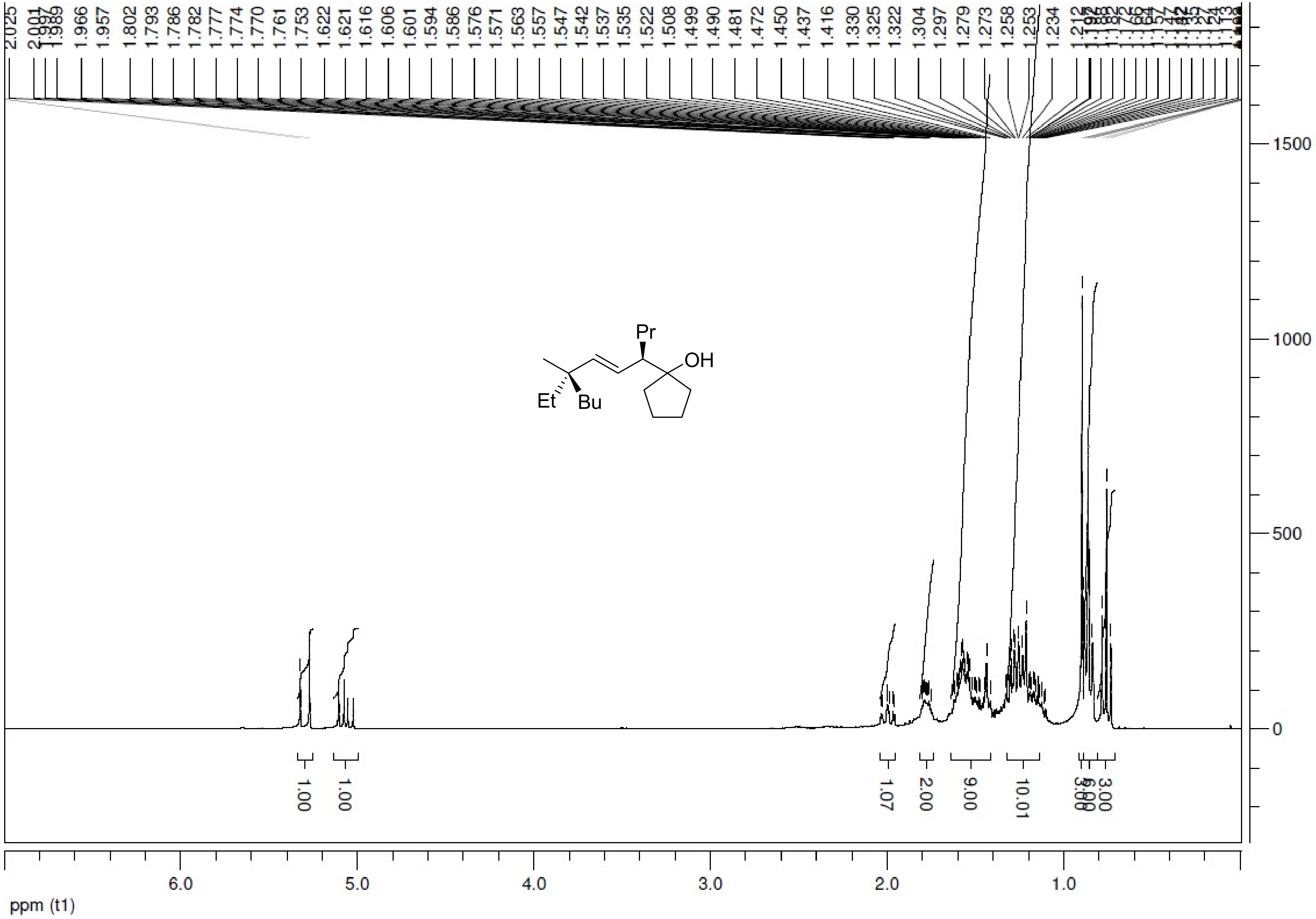
2.025
2.001
1.989
1.966
1.957
1.802
1.793
1.786
1.782
1.777
1.774
1.770
1.761
1.753
1.622
1.621
1.616
1.606
1.601
1.594
1.586
1.576
1.571
1.563
1.557
1.547
1.542
1.537
1.535
1.522
1.508
1.499
1.490
1.481
1.472
1.450
1.437
1.416
1.330
1.325
1.322
1.304
1.297
1.279
1.273
1.258
1.253
1.234
1.212
Pr
OH
Et
Bu
1500
1000
500
0
1.00
1.00
1.07
2.00
9.00
10.01
3.00
6.00
3.00
6.0
5.0
4.0
3.0
2.0
1.0
ppm (t1)

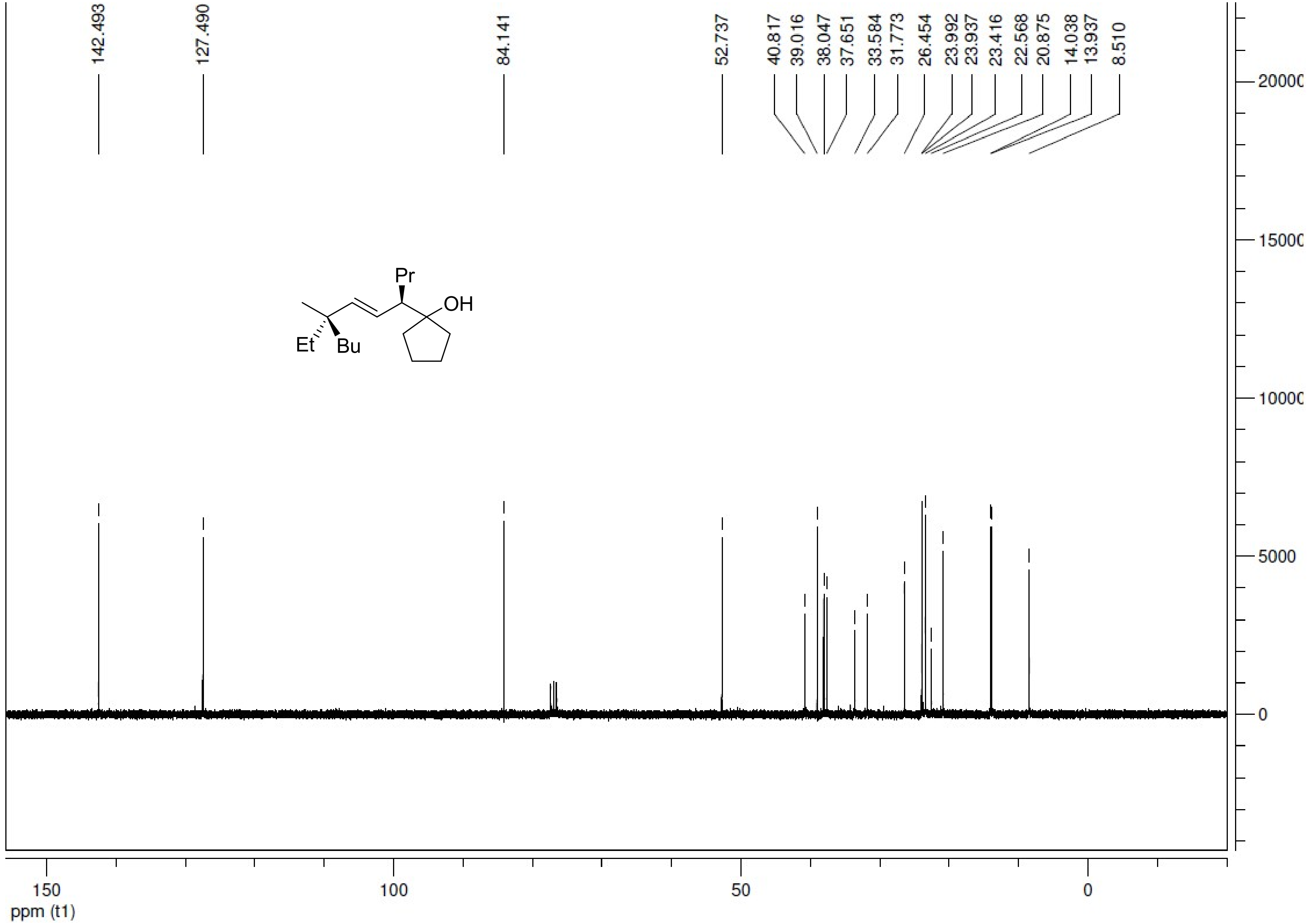

142.493
127.490
84.141
52.737
40.817
39.016
38.047
37.651
33.584
31.773
26.454
23.992
23.937
23.416
22.568
20.875
14.038
13.937
8.510
Pr
OH
Et
Bu
20000
15000
10000
5000
0
150
100
50
0
ppm (t1)

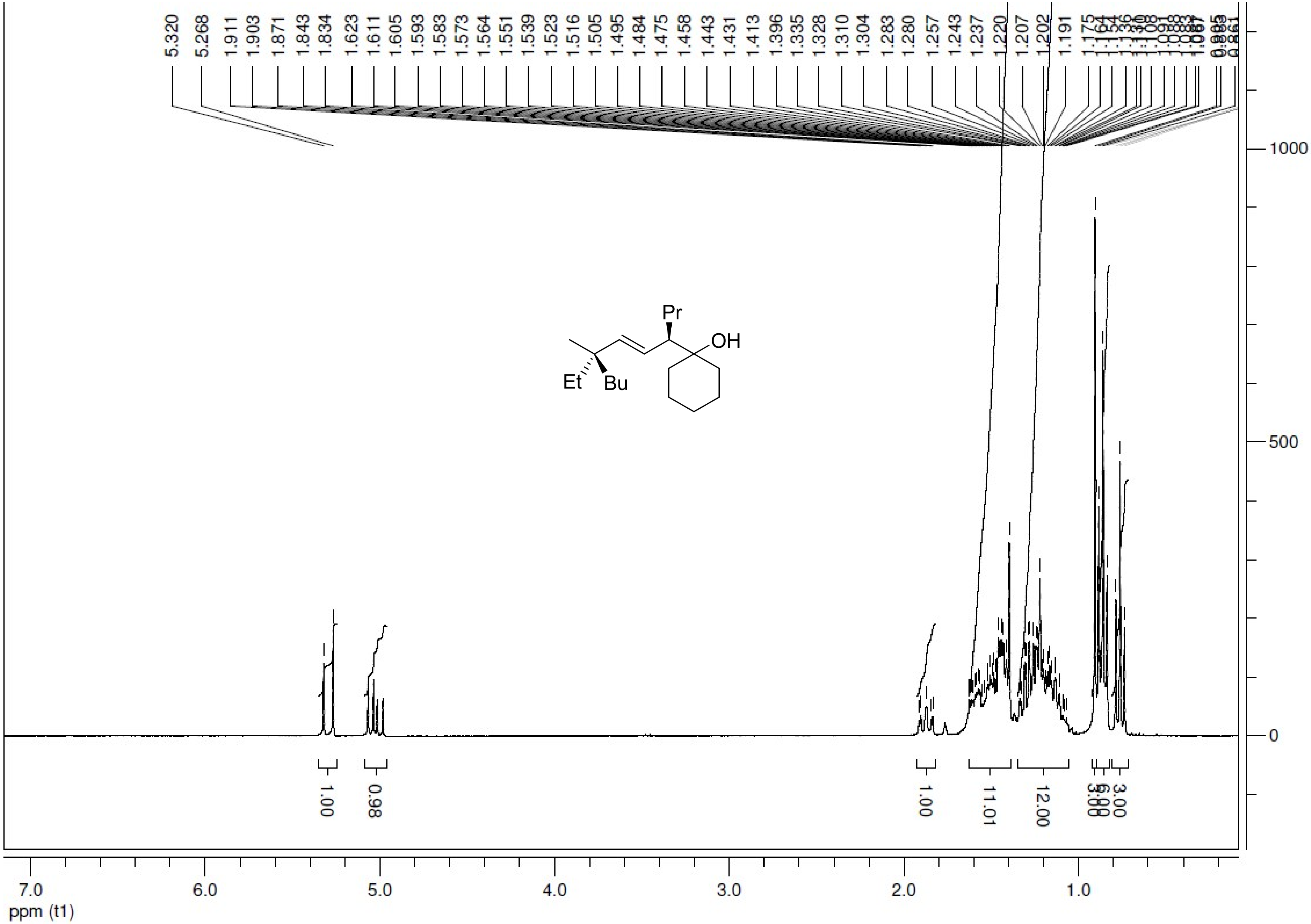

Pr
OH
Et
Bu
1.00
0.98
1.00
11.01
12.00
3.00
7.0
6.0
5.0
4.0
3.0
2.0
1.0
ppm (t1)
1000
500
0

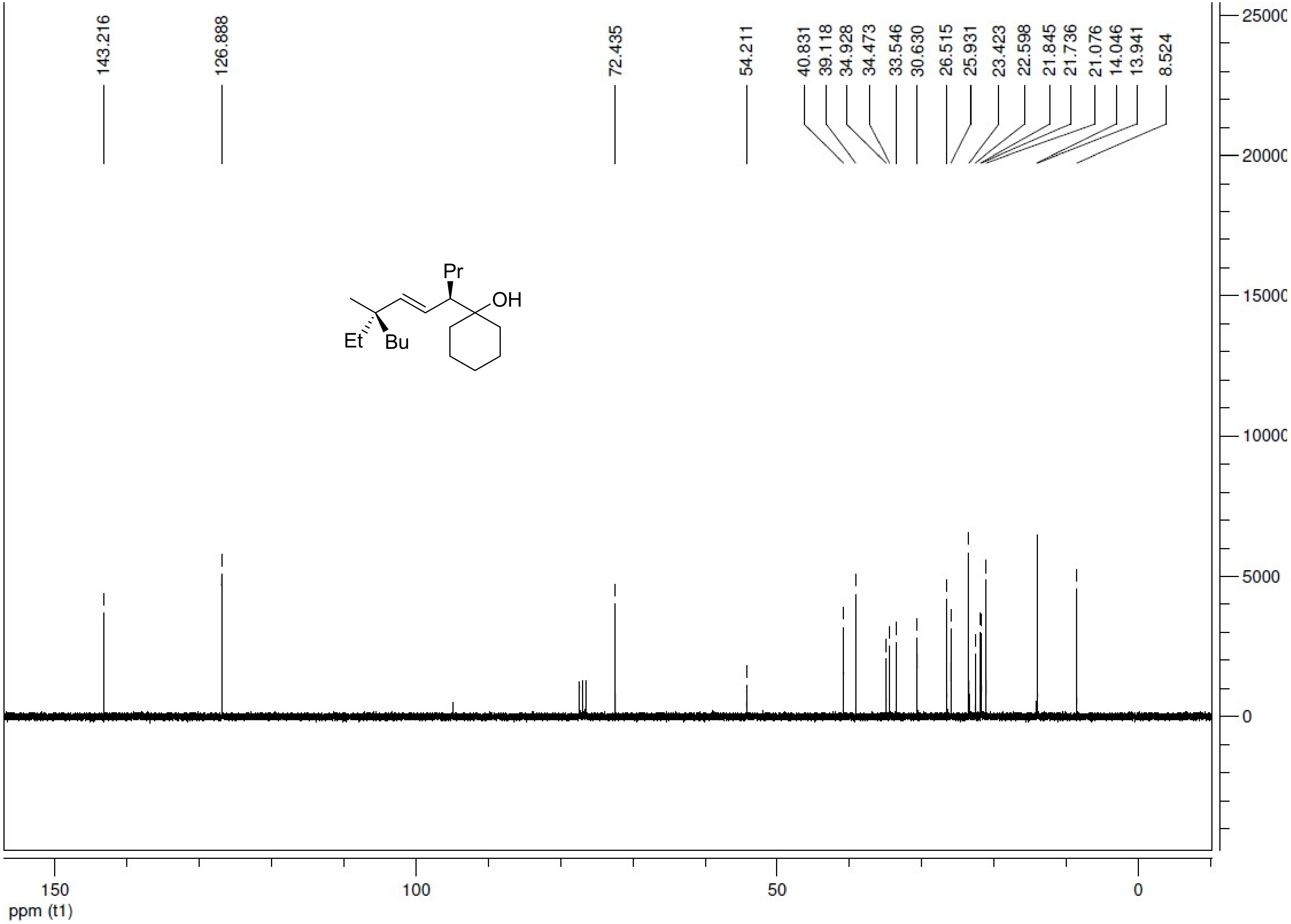
143.216
126.888
72.435
54.211
40.831
39.118
34.928
34.473
33.546
30.630
26.515
25.931
23.423
22.598
21.845
21.736
21.076
14.046
13.941
8.524
Pr
OH
Et
Bu
25000
20000
15000
10000
5000
0
150
100
50
0
ppm (t1)

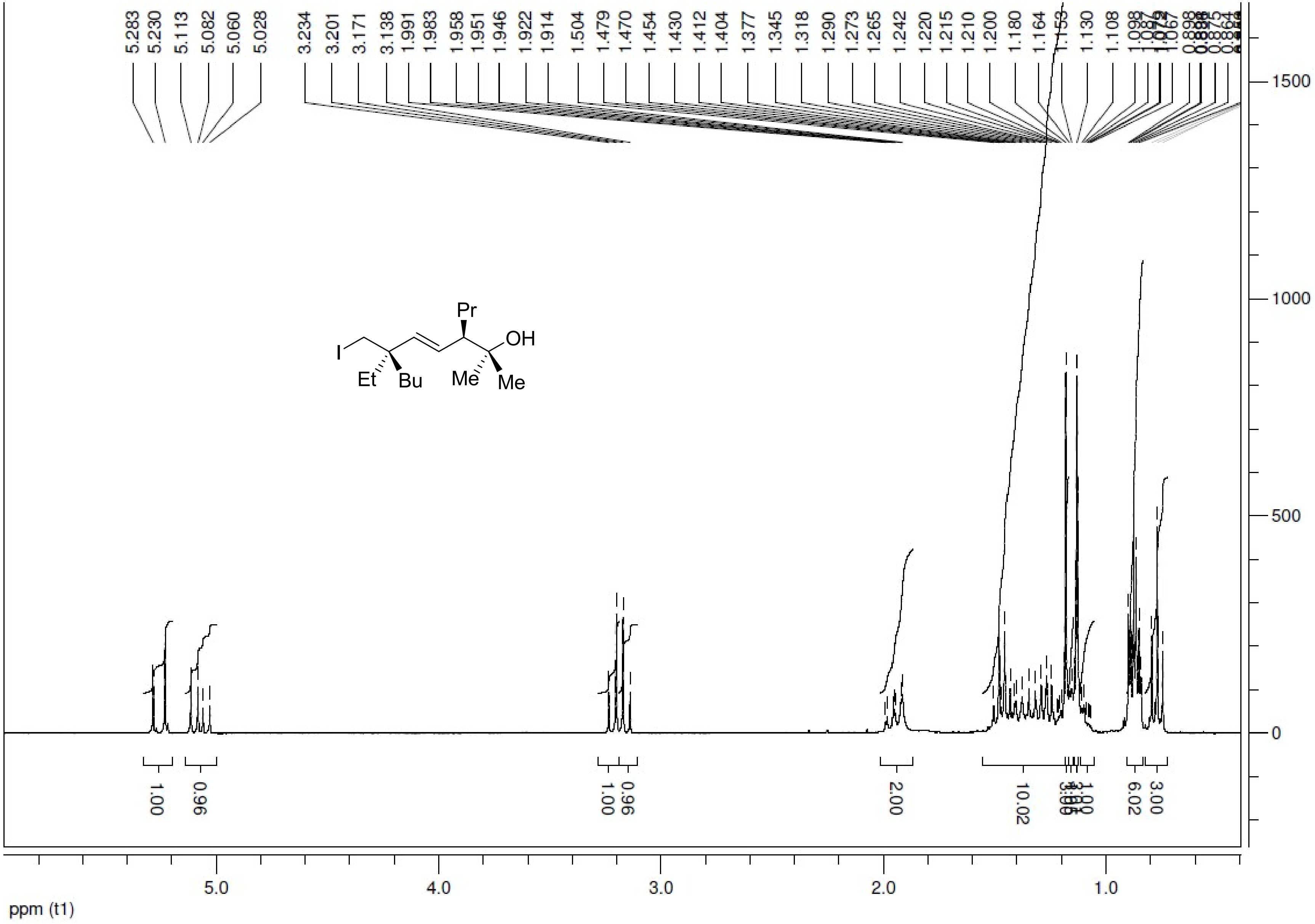

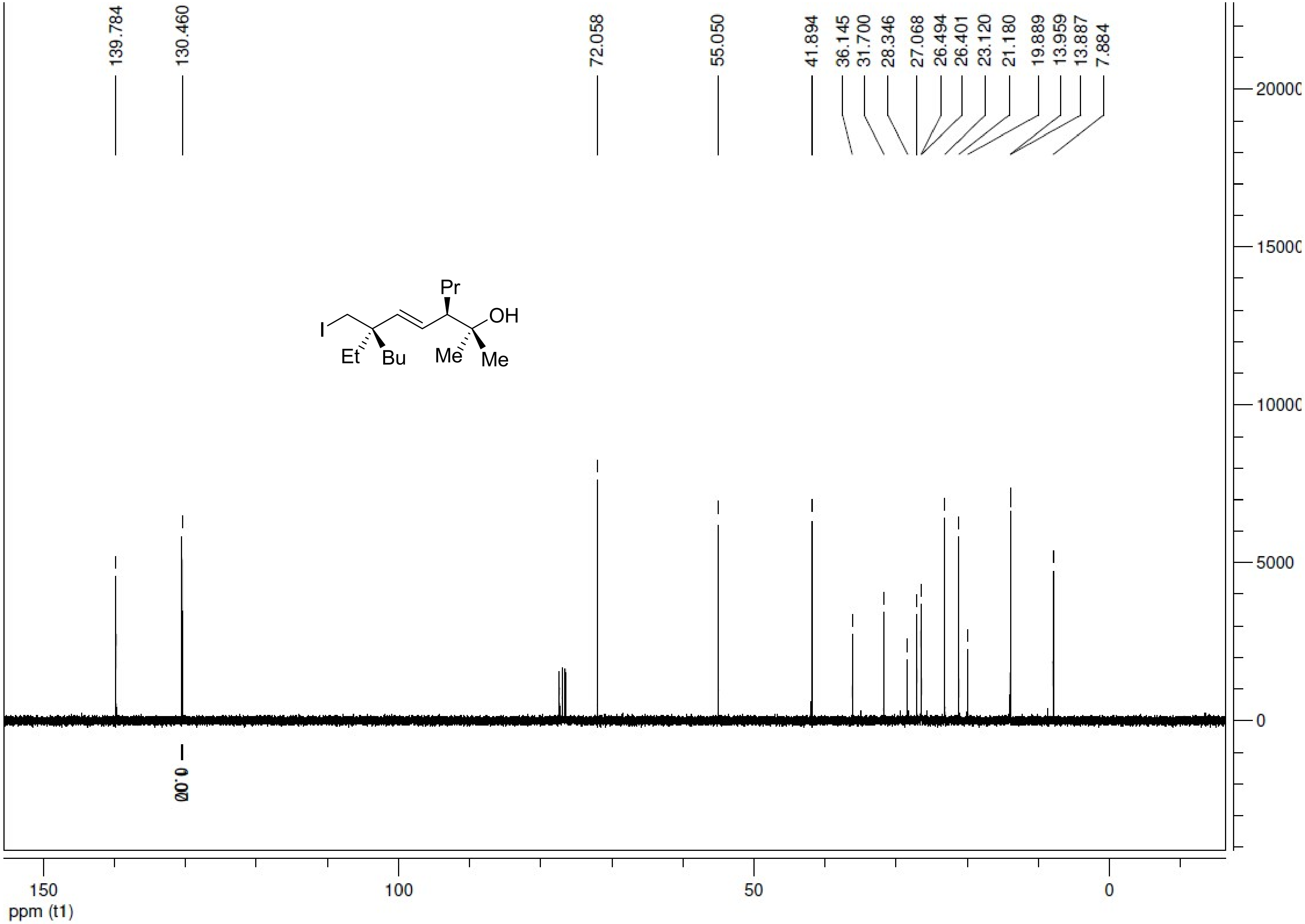
139.784
130.460
72.058
55.050
41.894
36.145
31.700
28.346
27.068
26.494
26.401
23.120
21.180
19.889
13.959
13.887
7.884
Pr
OH
I
Et
Bu
Me
Me
20000
15000
10000
5000
0
0.00
150
100
50
0
ppm (t1)

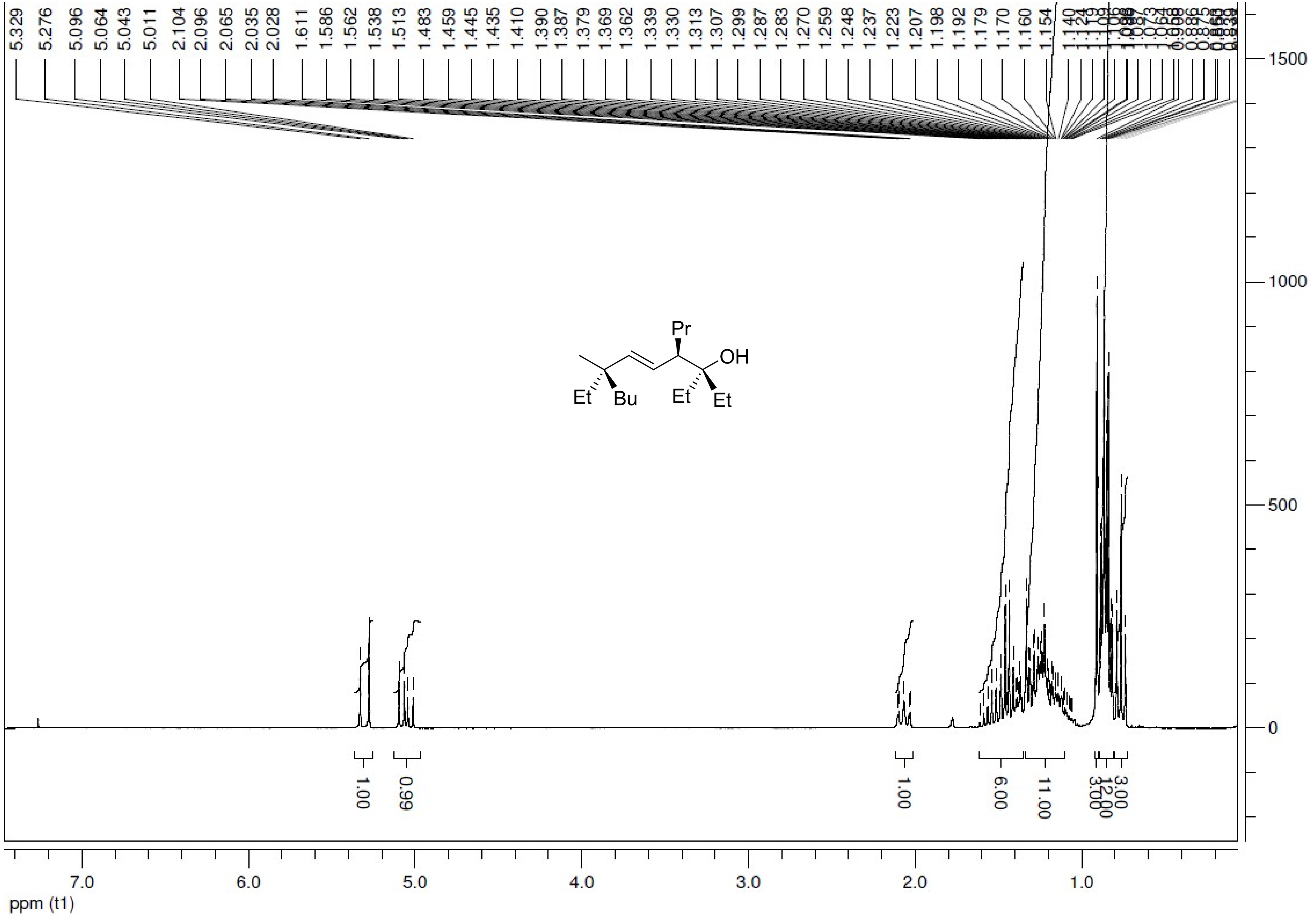
Pr
OH
Et
Bu
Et
Et
ppm (t1)
1.00
0.99
1.00
6.00
11.00
3.00
12.00
3.00

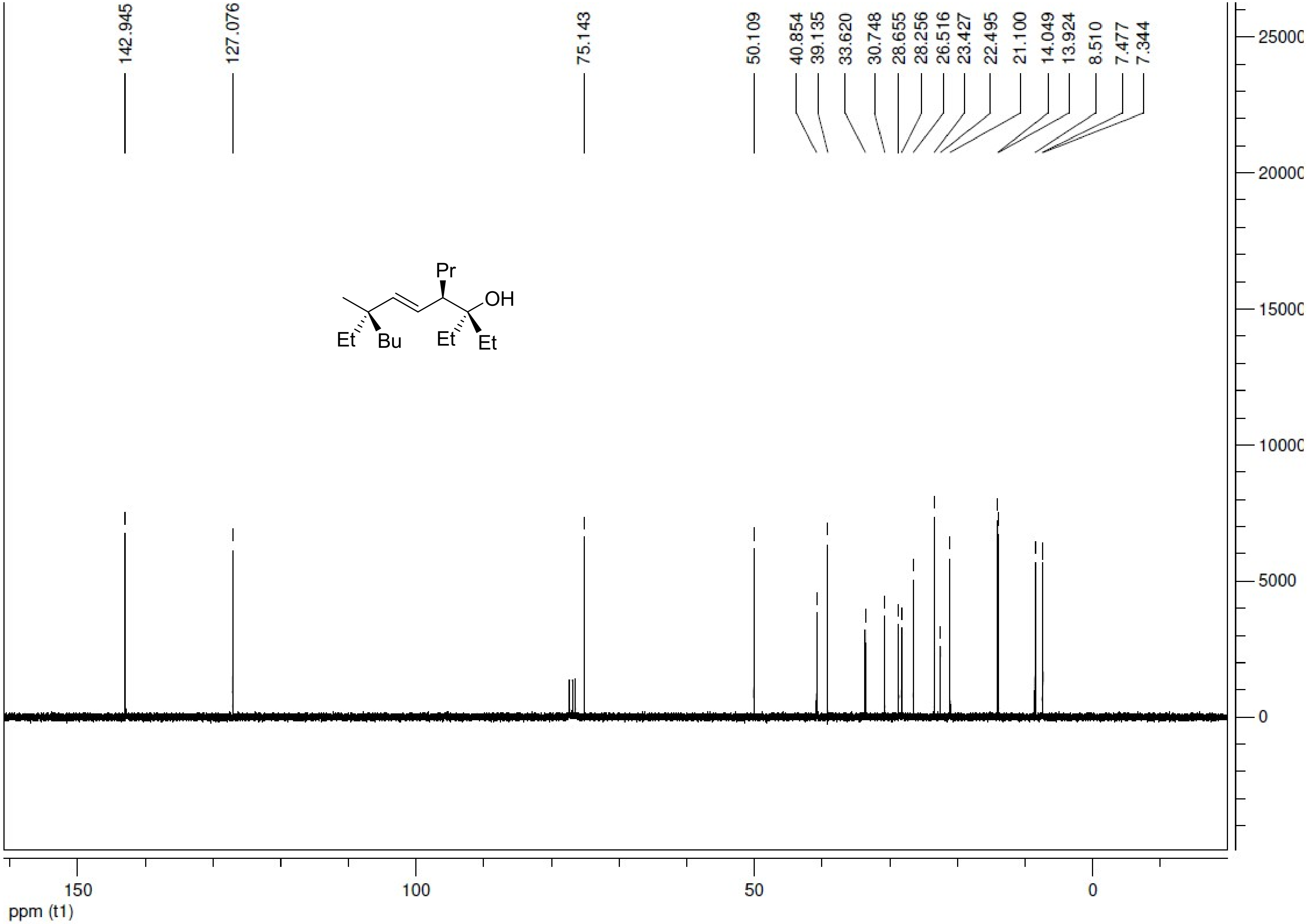
142.945
127.076
75.143
50.109
40.854
39.135
33.620
30.748
28.655
28.256
26.516
23.427
22.495
21.100
14.049
13.924
8.510
7.477
7.344
Pr
OH
Et
Bu
Et
Et
25000
20000
15000
10000
5000
0
150
100
50
0
ppm (t1)

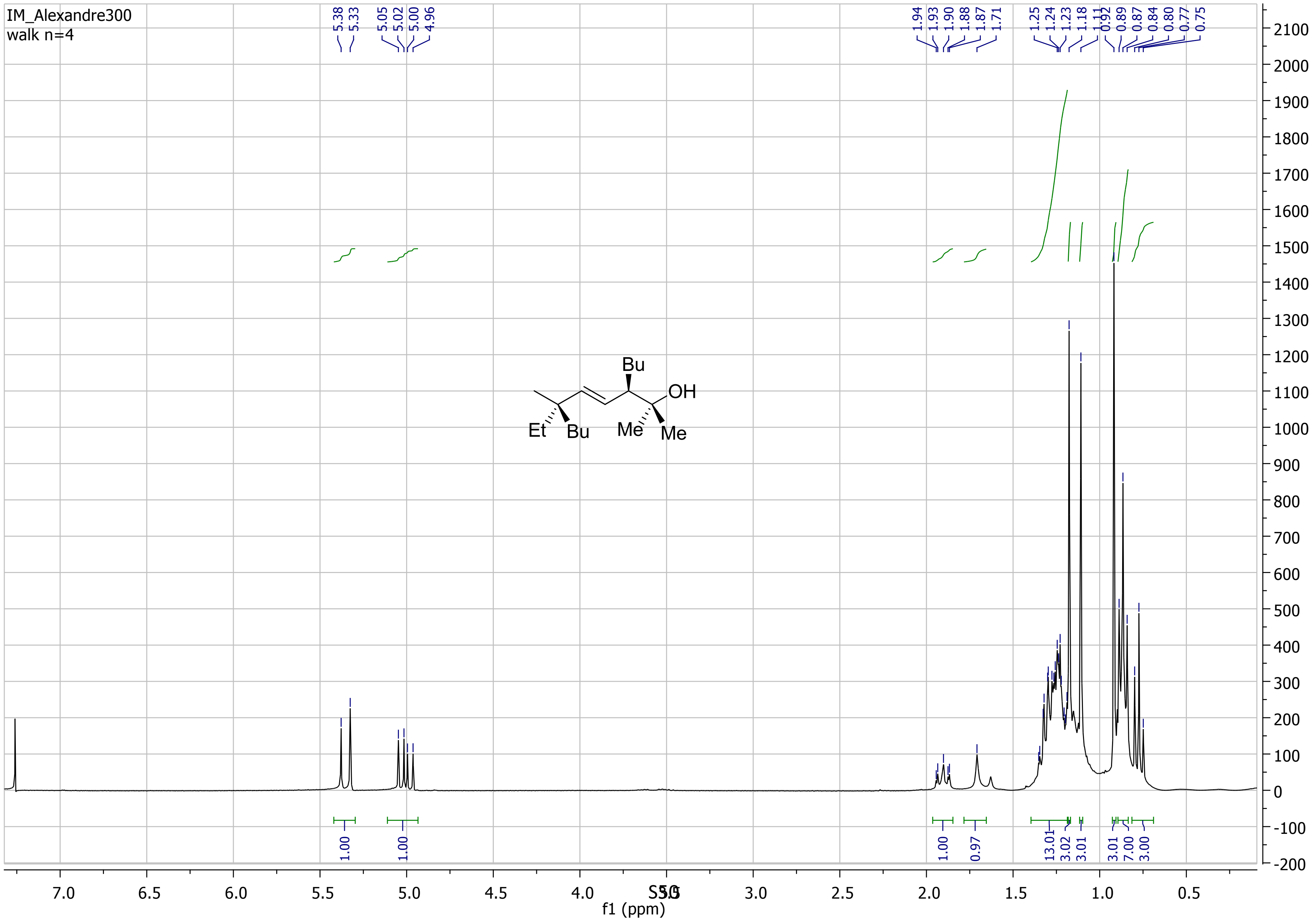
IM_Alexandre300
walk n=4
5.38
5.33
5.05
5.02
5.00
4.96
1.94
1.93
1.90
1.88
1.87
1.71
1.25
1.24
1.23
1.18
1.11
0.92
0.89
0.87
0.84
0.80
0.77
0.75
Bu
OH
Et
Bu
Me
Me
1.00
1.00
1.00
0.97
13.01
3.02
3.01
3.01
7.00
3.00
7.0
6.5
6.0
5.5
5.0
4.5
4.0
3.5
3.0
2.5
2.0
1.5
1.0
0.5
f1 (ppm)
2100
2000
1900
1800
1700
1600
1500
1400
1300
1200
1100
1000
900
800
700
600
500
400
300
200
100
0
-100
-200

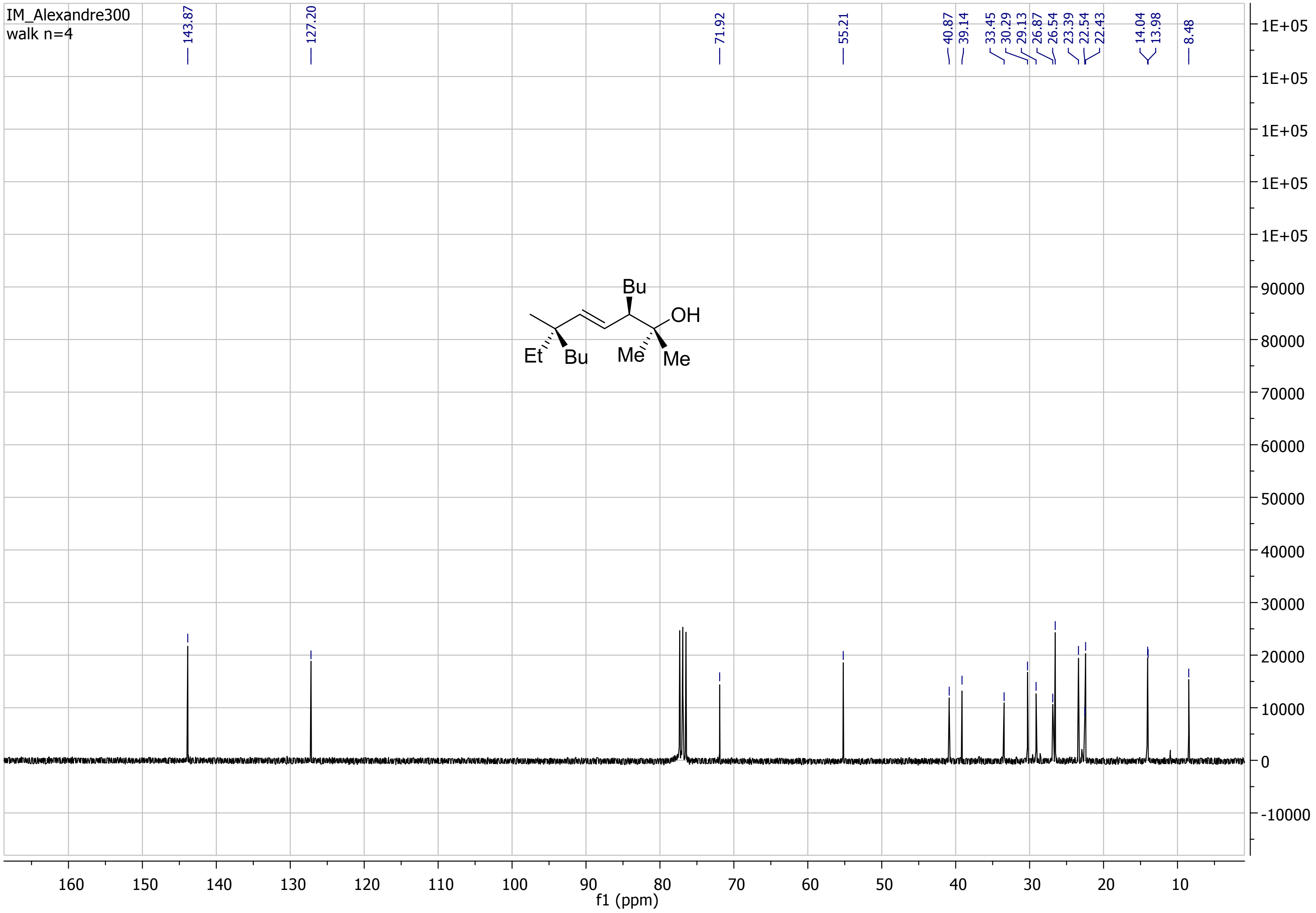
IM_Alexandre300
walk n=4
143.87
127.20
71.92
55.21
40.87
39.14
33.45
30.29
29.13
26.87
26.54
23.39
22.54
22.43
14.04
13.98
8.48
Bu
OH
Et
Bu
Me
Me
f1 (ppm)

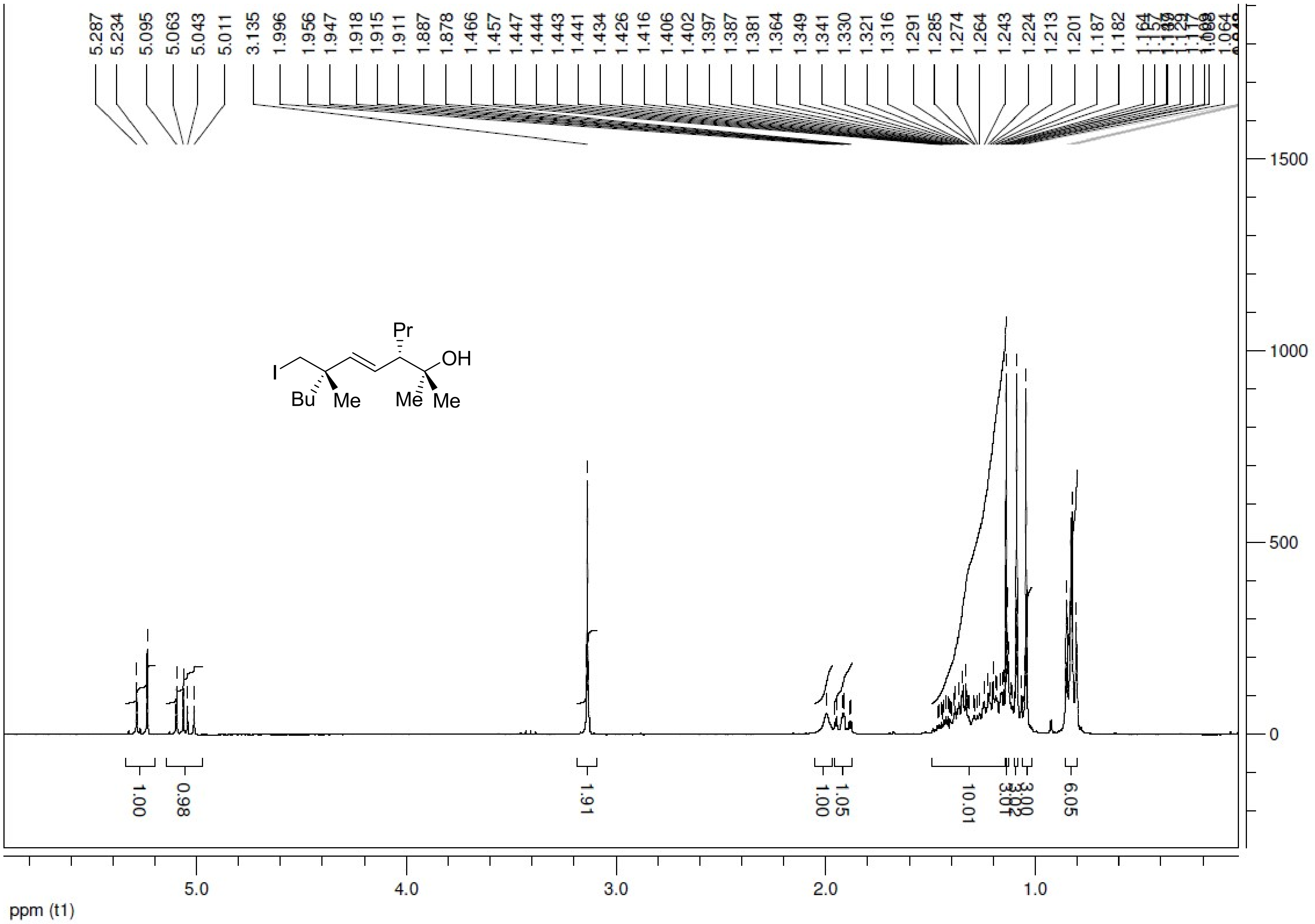

I
Bu
Me
Pr
OH
Me
Me
ppm (t1)
5.0
4.0
3.0
2.0
1.0
1500
1000
500
0
1.00
0.98
1.91
1.00
1.05
10.01
6.05

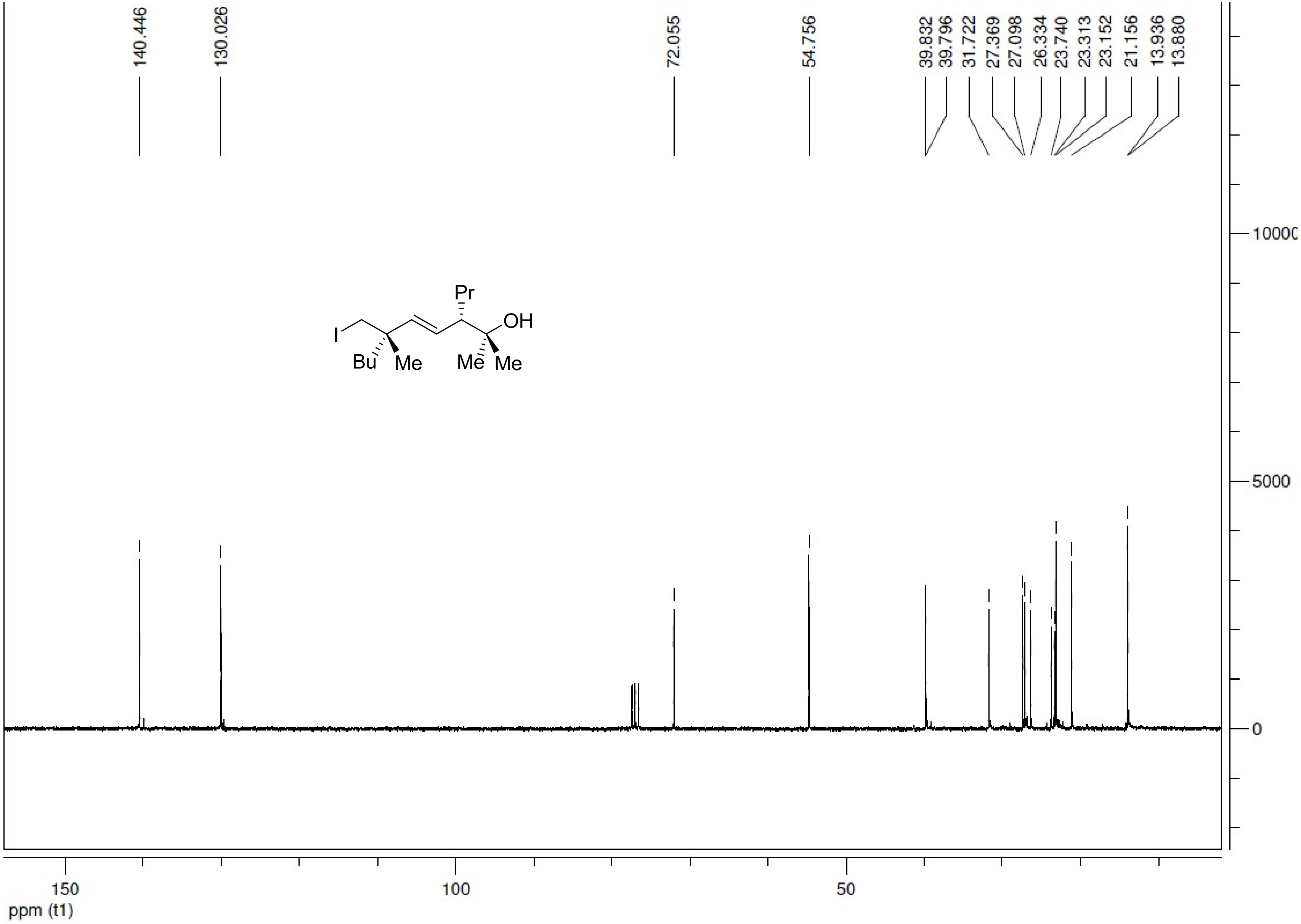

140.446
130.026
72.055
54.756
39.832
39.796
31.722
27.369
27.098
26.334
23.740
23.313
23.152
21.156
13.936
13.880
Pr
OH
I
Bu
Me
Me
Me
10000
5000
0
150
100
50
ppm (t1)

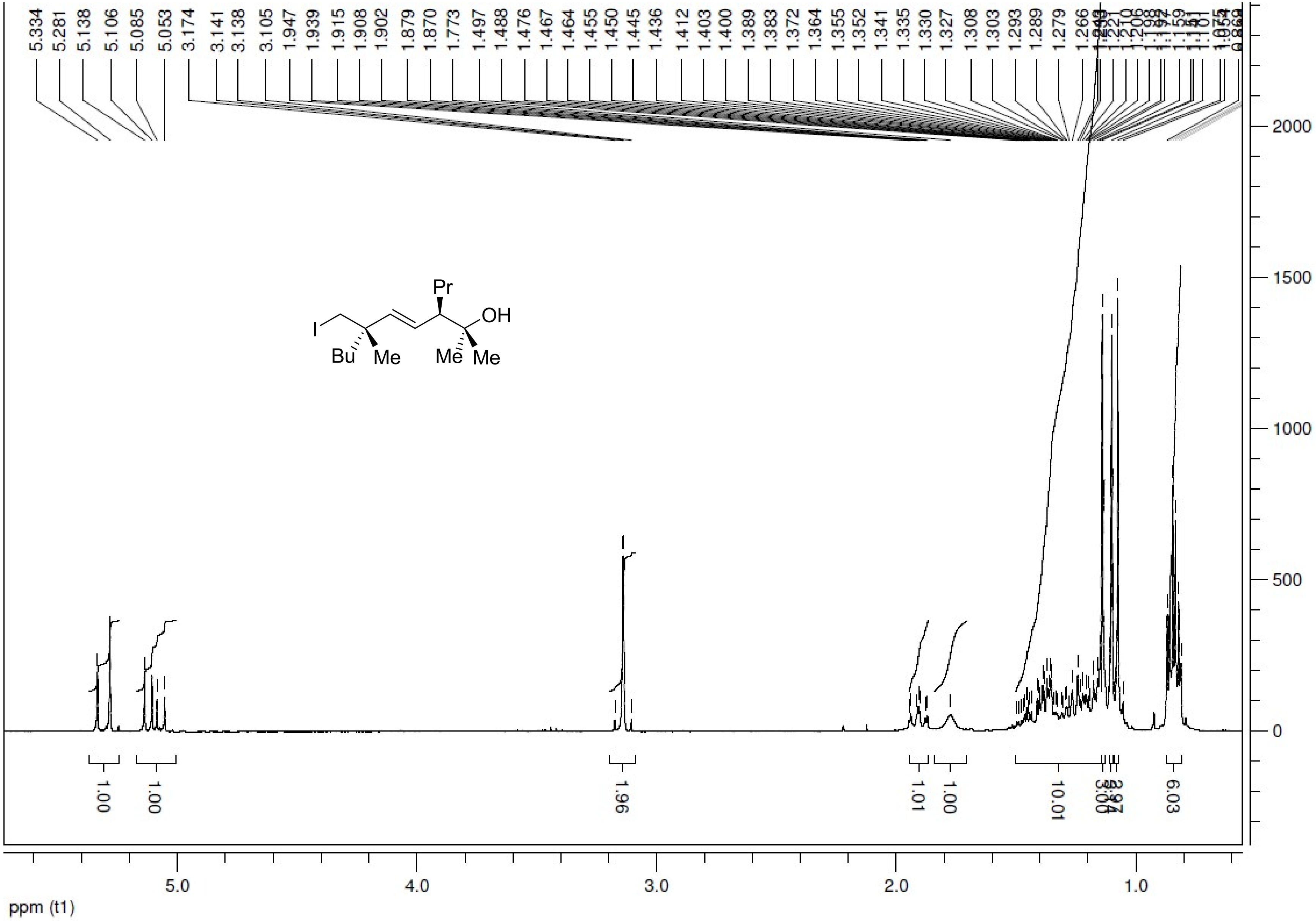

ppm (t1)
5.0
4.0
3.0
2.0
1.0
Pr
OH
I
Bu
Me
Me
Me

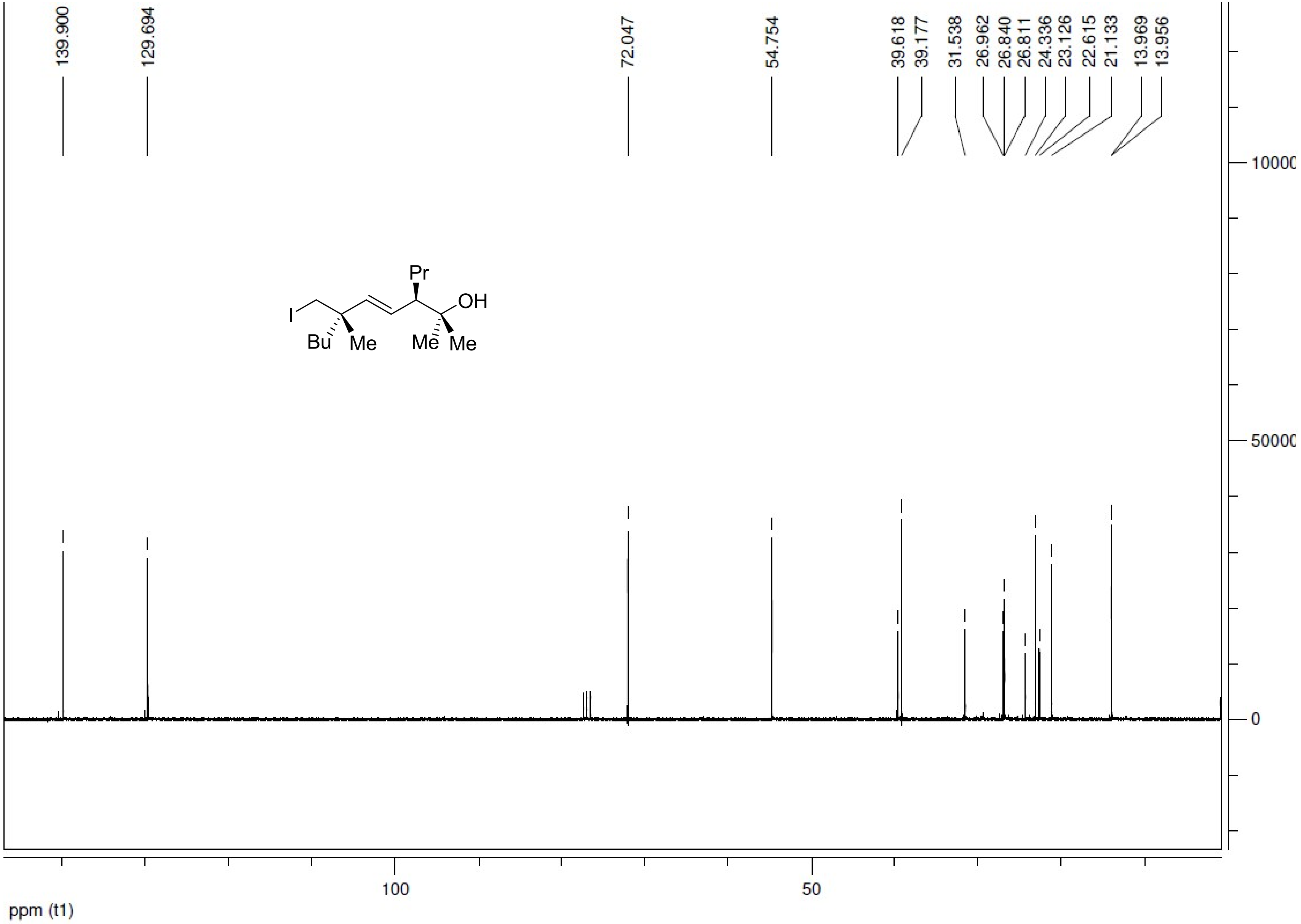
139.900
129.694
72.047
54.754
39.618
39.177
31.538
26.962
26.840
26.811
24.336
23.126
22.615
21.133
13.969
13.956
Pr
OH
I
Bu
Me
Me
Me
100
50
ppm (t1)
0

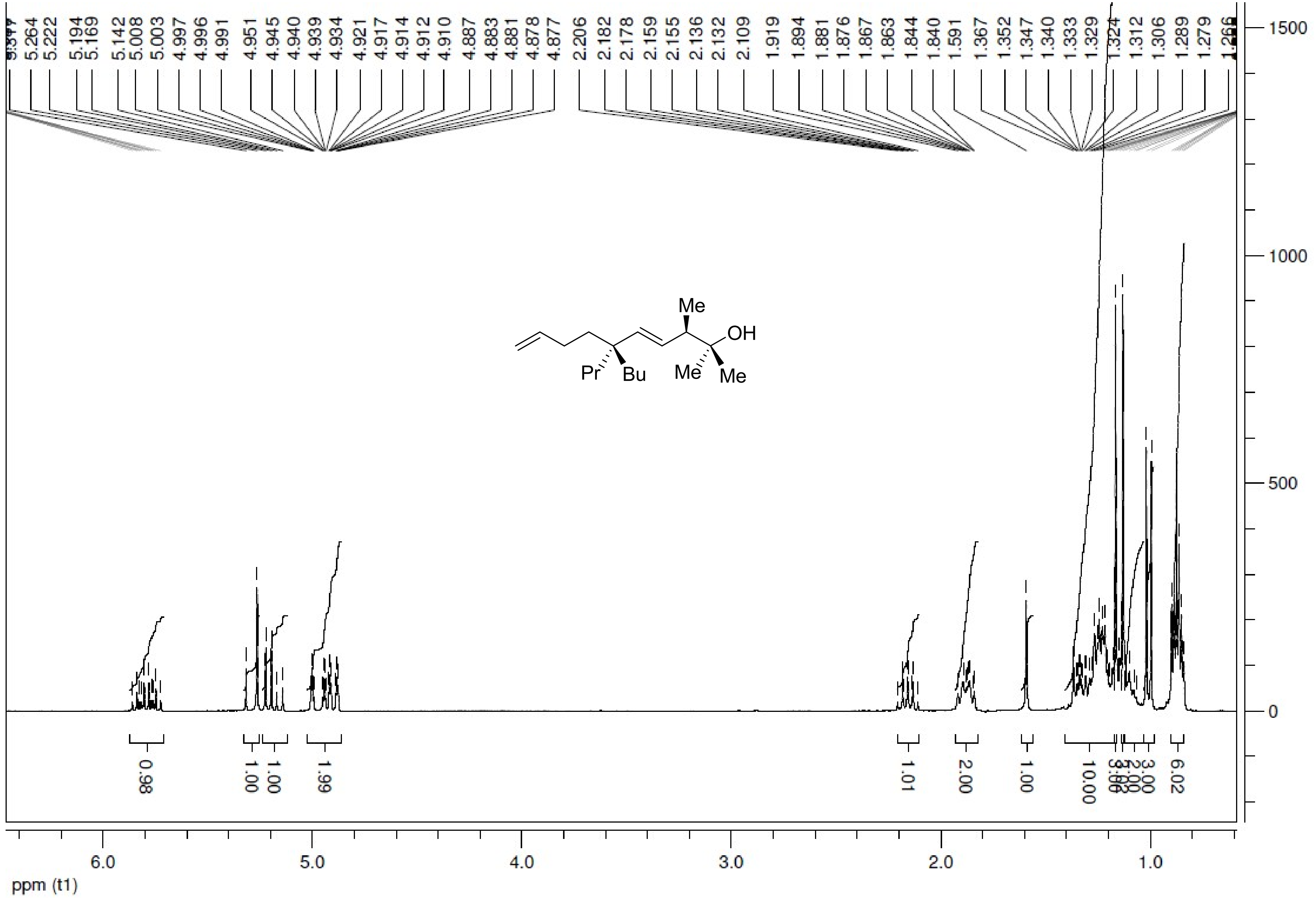

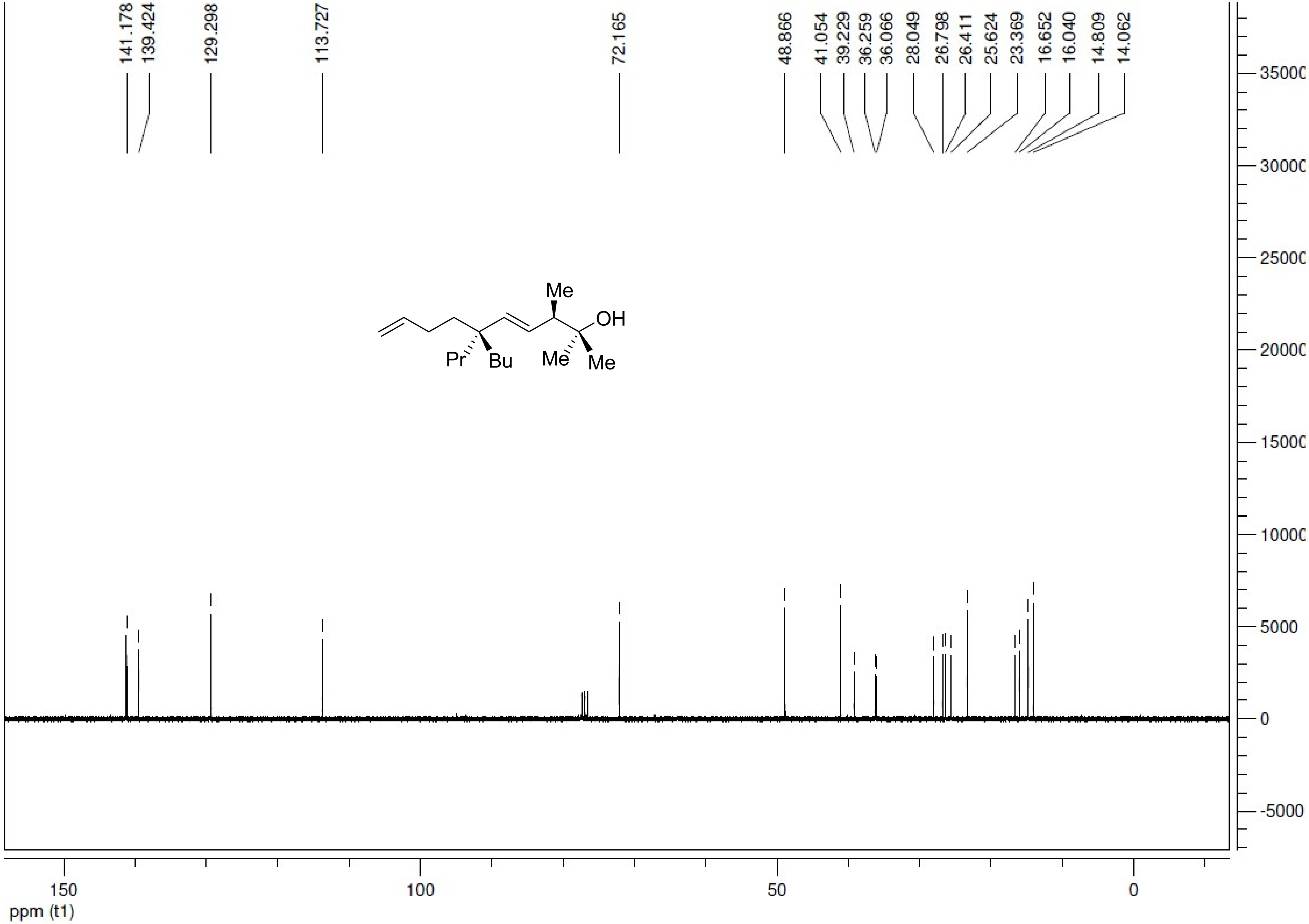

141.178
139.424
129.298
113.727
72.165
48.866
41.054
39.229
36.259
36.066
28.049
26.798
26.411
25.624
23.369
16.652
16.040
14.809
14.062
Me
OH
Pr
Bu
Me
Me
35000
30000
25000
20000
15000
10000
5000
0
-5000
150
100
50
0
ppm (t1)

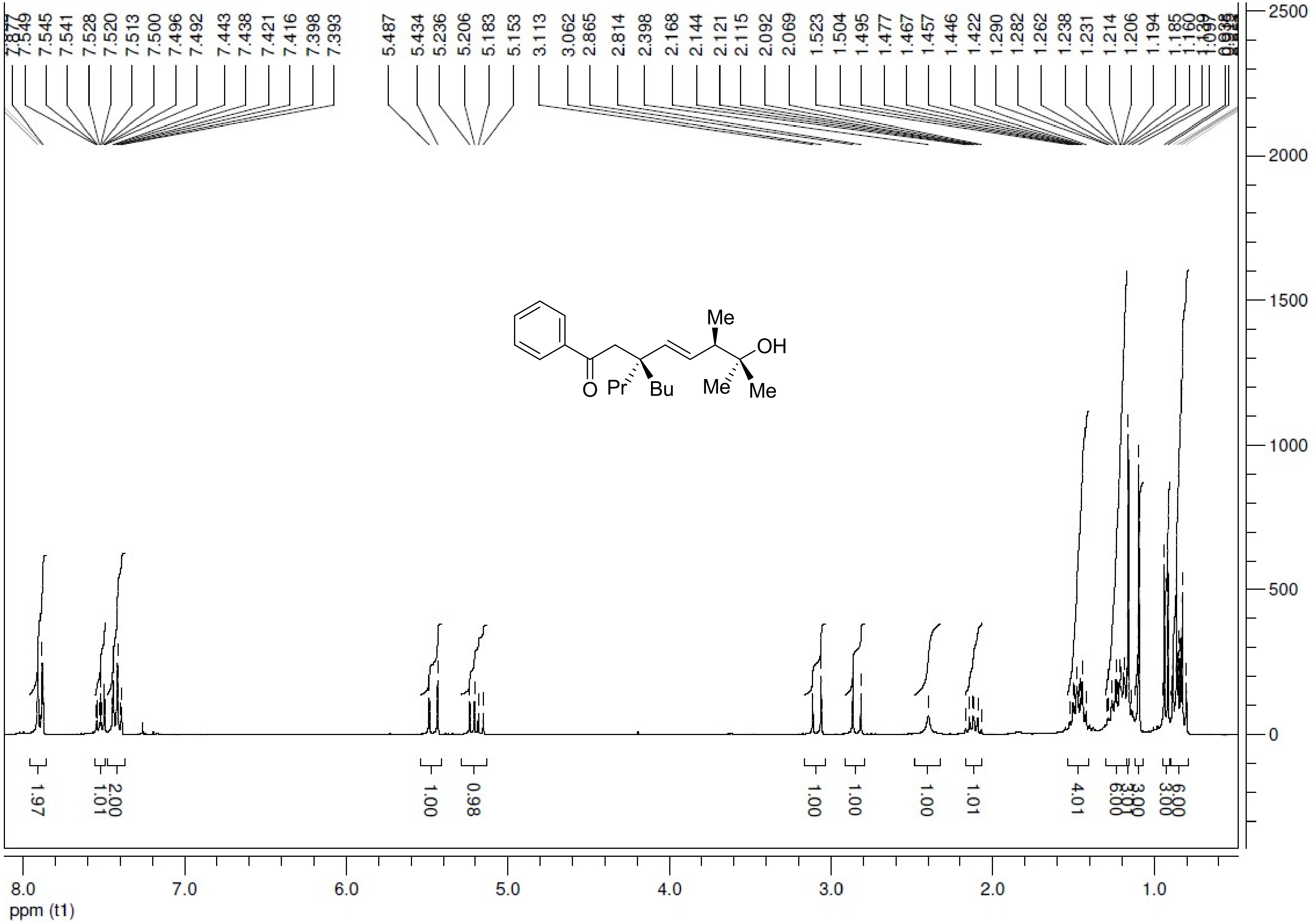

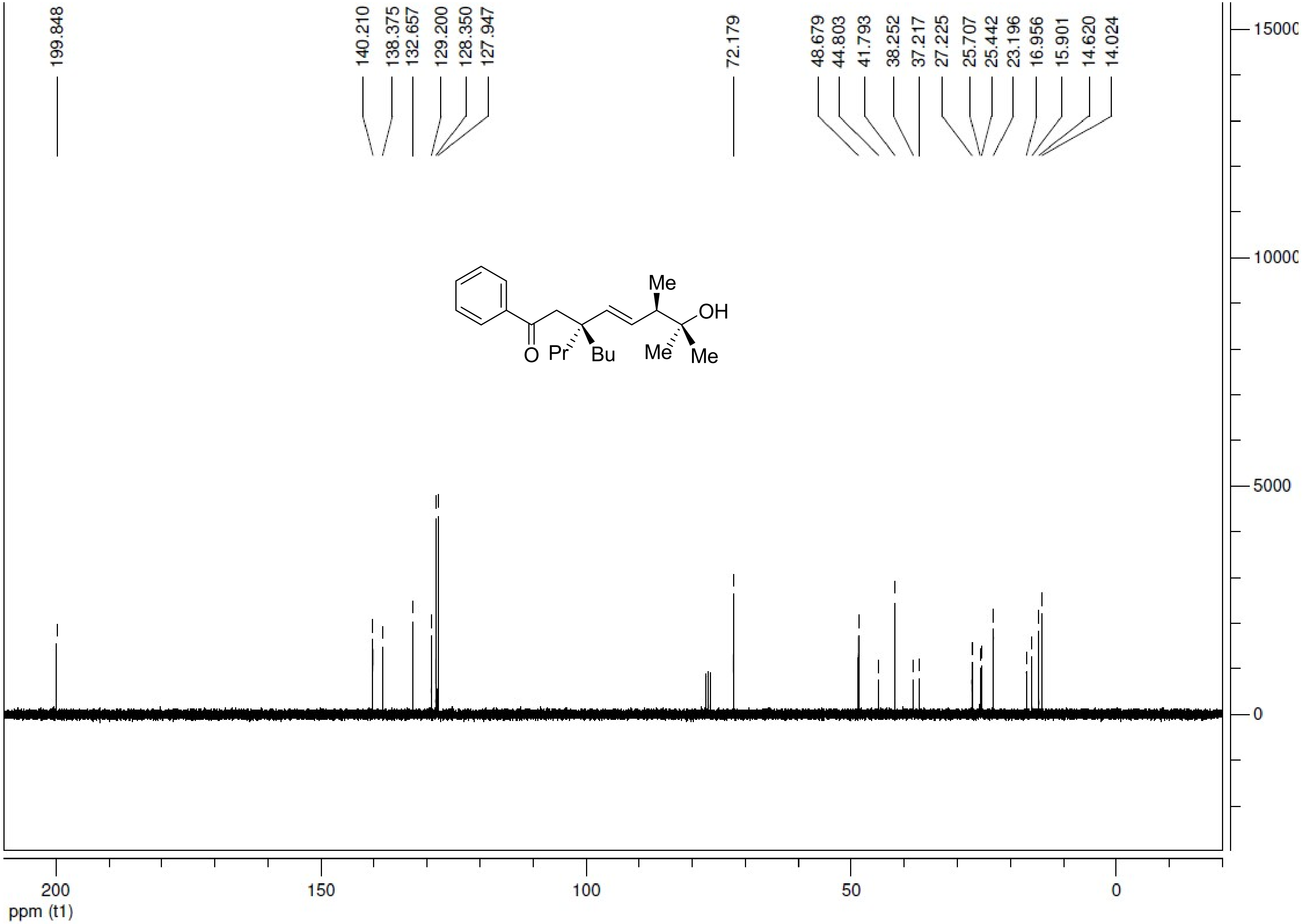
199.848
140.210
138.375
132.657
129.200
128.350
127.947
72.179
48.679
44.803
41.793
38.252
37.217
27.225
25.707
25.442
23.196
16.956
15.901
14.620
14.024
15000
10000
5000
0
Me
OH
O
Pr
Bu
Me
Me
200
150
100
50
0
ppm (t1)

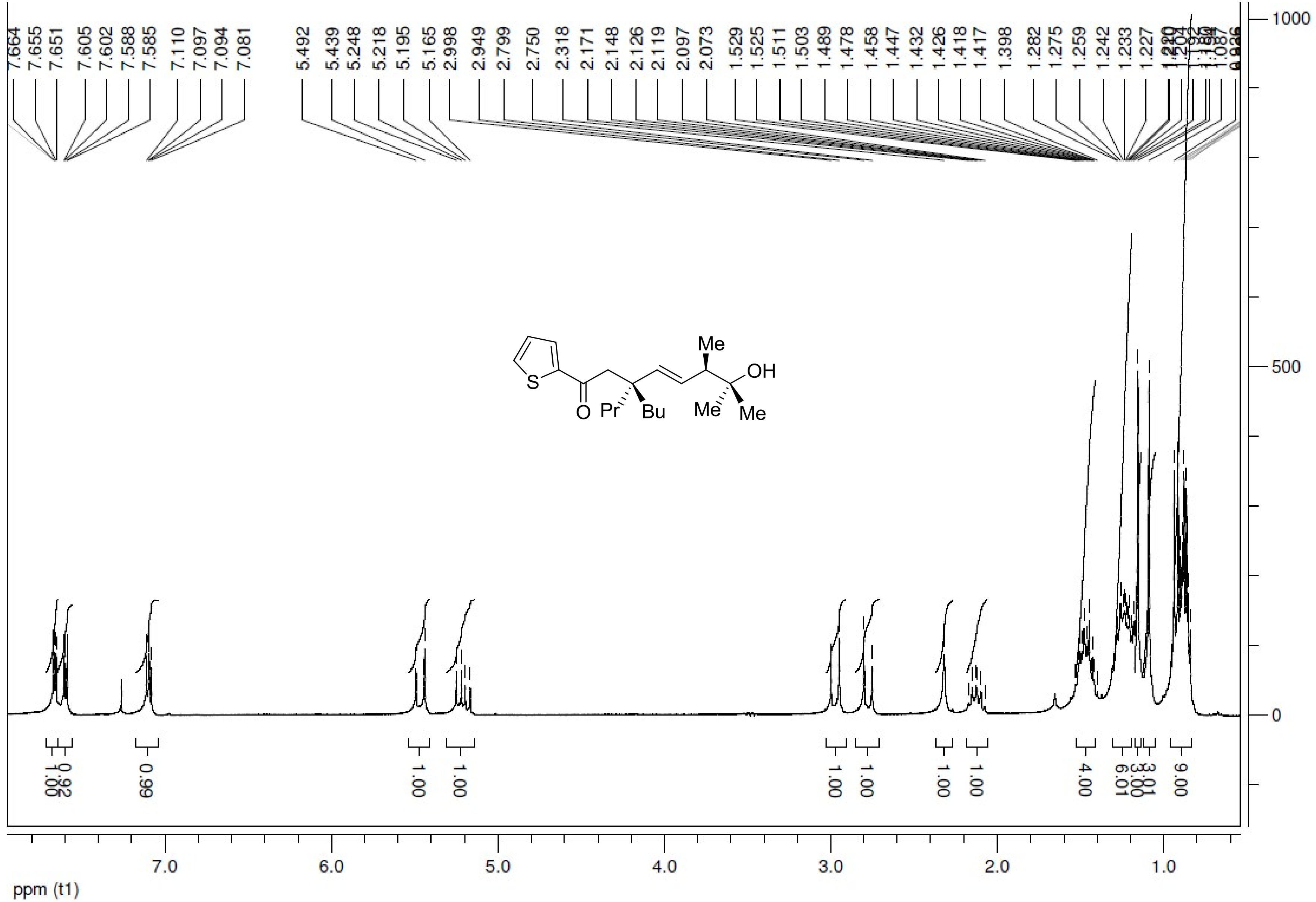

7.664
7.655
7.651
7.605
7.602
7.588
7.585
7.110
7.097
7.094
7.081
5.492
5.439
5.248
5.218
5.195
5.165
2.998
2.949
2.799
2.750
2.318
2.171
2.148
2.126
2.119
2.097
2.073
1.529
1.525
1.511
1.503
1.489
1.478
1.458
1.447
1.432
1.426
1.418
1.417
1.398
1.282
1.275
1.259
1.242
1.233
1.227
S
O
Pr
Bu
Me
OH
Me
Me
1.00
0.92
0.99
1.00
1.00
1.00
1.00
1.00
1.00
4.00
6.01
3.00
3.01
9.00
1000
500
0
7.0
6.0
5.0
4.0
3.0
2.0
1.0
ppm (t1)

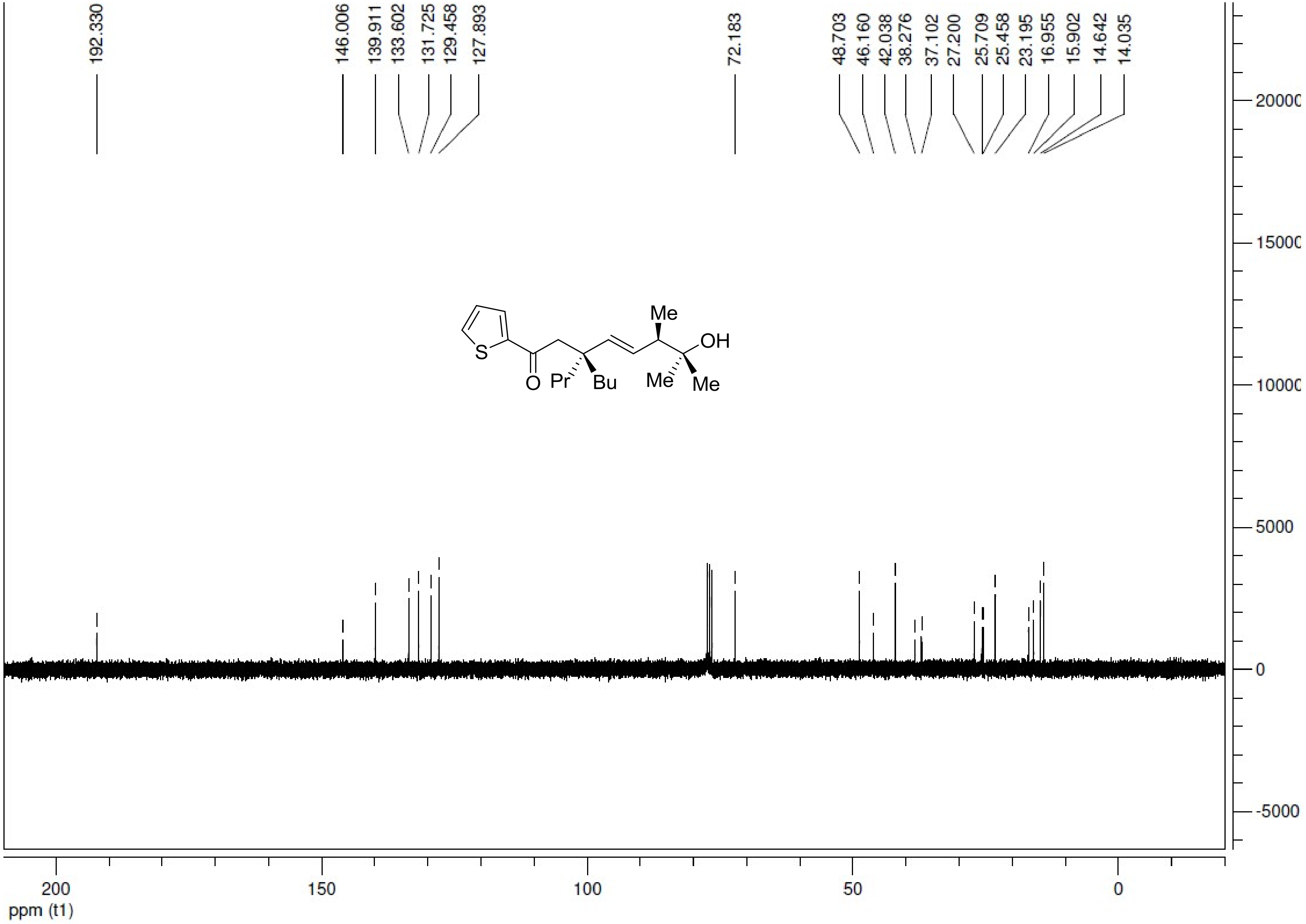
192.330
146.006
139.911
133.602
131.725
129.458
127.893
72.183
48.703
46.160
42.038
38.276
37.102
27.200
25.709
25.458
23.195
16.955
15.902
14.642
14.035
S
O
Pr
Bu
Me
OH
Me
Me
200
150
100
50
0
ppm (t1)
20000
15000
10000
5000
0
-5000

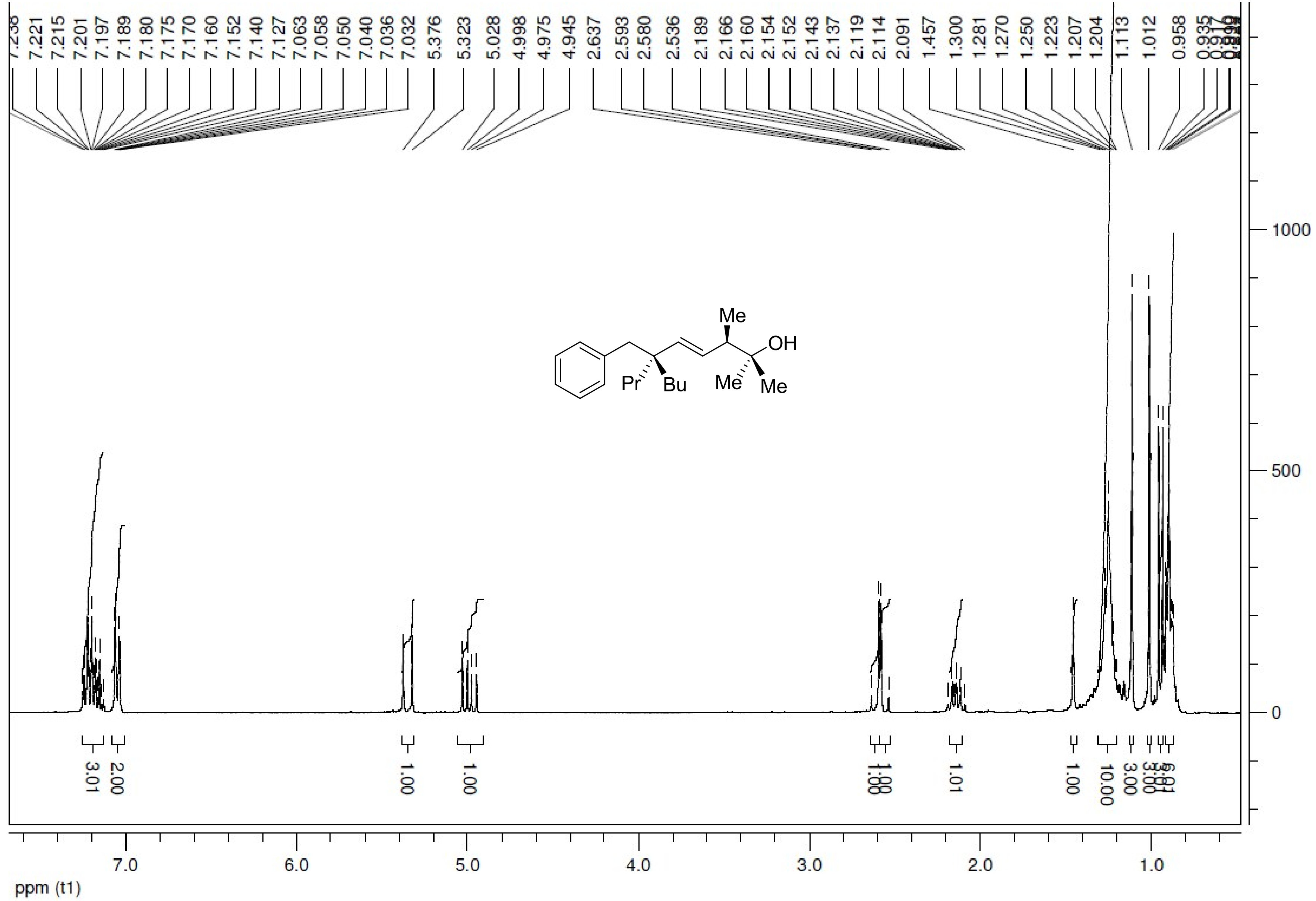

Me
OH
Pr
Bu
Me
Me
ppm (t1)

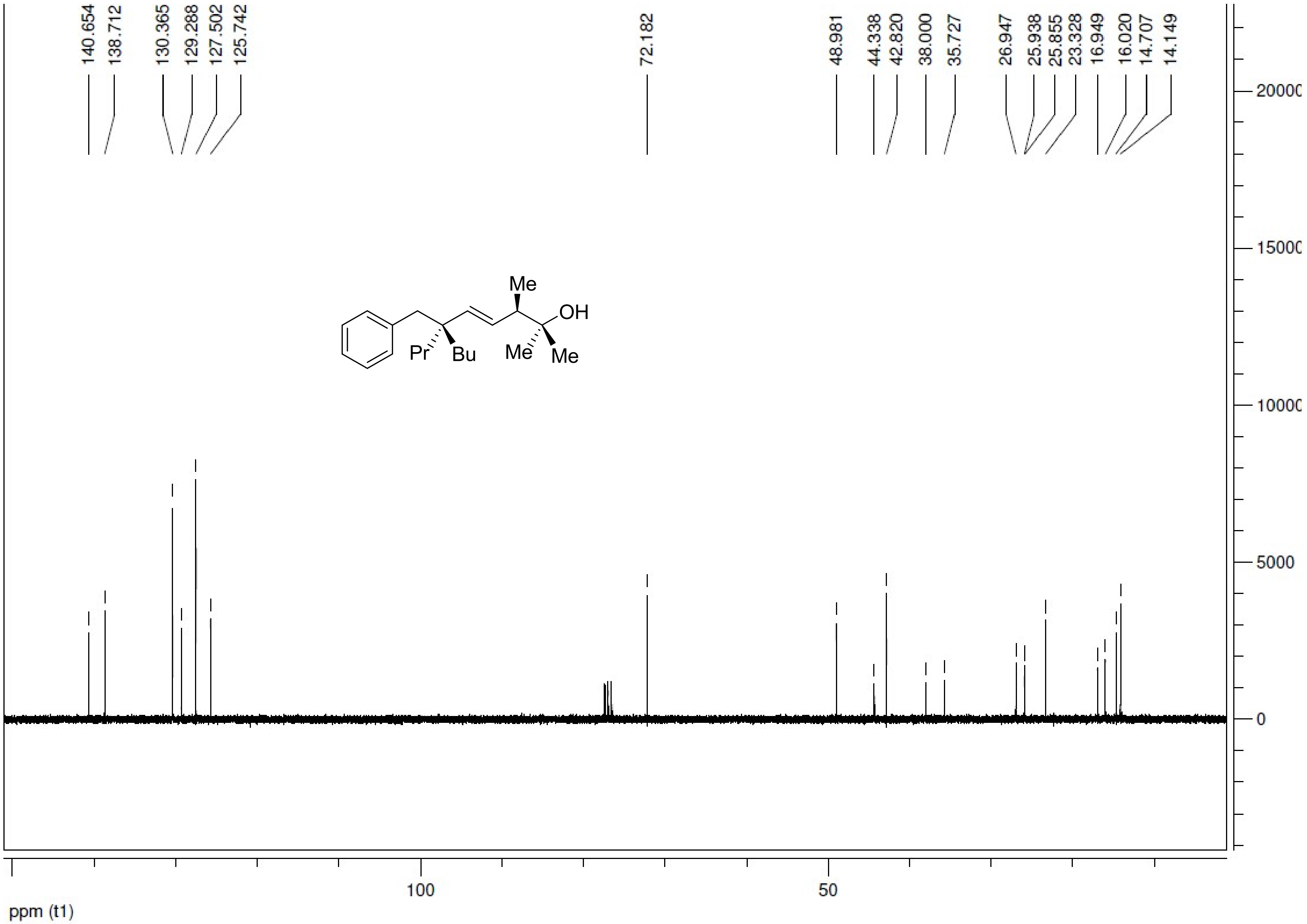
140.654
138.712
130.365
129.288
127.502
125.742
72.182
48.981
44.338
42.820
38.000
35.727
26.947
25.938
25.855
23.328
16.949
16.020
14.707
14.149
Me
OH
Pr
Bu
Me
Me
20000
15000
10000
5000
0
100
50
ppm (t1)
